# Functional Metal Complexes for Solar–Light‐Driven Energy Conversion

**DOI:** 10.1002/adma.202518393

**Published:** 2026-03-21

**Authors:** Yanyan Qin, Dou Luo, Miao Zhang, Wai‐Yeung Wong

**Affiliations:** ^1^ Department of Applied Biology and Chemical Technology and Research Institute for Smart Energy The Hong Kong Polytechnic University Hung Hom Hong Kong P. R. China; ^2^ The Hong Kong Polytechnic University Shenzhen Research Institute Shenzhen P. R. China

**Keywords:** metal complex, solar energy, solar–to–electrical energy conversion, solar–to–chemical energy conversion, solar–to–thermal energy conversion

## Abstract

Solar energy conversion offers the advantage of being more environmentally friendly compared to the use of nuclear or fossil fuels. In recent years, metal complexes as functional materials have received extensive attention, and great progress has been made in energy storage and conversion. They usually possess tunable photoelectrochemical properties due to the diversity of organic ligands and metal centers. Moreover, the photophysical properties of metal complexes, particularly their strong ability to capture visible light and achieve long triplet state lifetimes, are essential for the effective utilization of these complexes in their practical applications. In this review, we examine the latest advancements in the design of the state‐of‐the art metal complexes and highlight key breakthroughs of these complexes, particularly in solar‐to‐electrical, ‐chemical, and ‐thermal energy conversion applications. We also address the current challenges and outline future directions for the development of functional metal complexes in the field of solar‐light‐driven energy conversion.

## Graphical abstract

This review provides an exhaustive summary of the recent advancements of functional metal complexes in solar‐to‐electrical, ‐chemical, and ‐thermal energy conversion, comprising material categories, design strategies, underlying principles of operation, and identified improvement of performances. Furthermore, the current challenges and future directions of metal complexes in solar‐light‐driven energy conversion processes are also outlined.

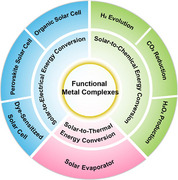

## 1 Introduction

Solar energy is a virtually limitless resource that can be harnessed to meet the growing demand of our society for electrical, chemical, thermal, and other forms of energy. The conversion of solar energy to other energy forms is crucial in advancing the shift toward a more sustainable energy system [1]. In this sense, many renewable solar energy conversion techniques, such as photovoltaics [2], photocatalysis [3] and photothermal processes [4] have been developed to achieve efficient photoelectrochemical energy storage and conversion. As a significant solar–electrical energy conversion technology, photovoltaic applications occupy an irreplaceable status indeed. Up to now, commercial photovoltaic technology has used silicon cells, which are divided into several types and subclasses according to their compositions. Though considerable power conversion efficiency (PCE) and decades of service life of silicon cells have been achieved, the high cost of production, heavy weight, and serious pollution problems are still challenging the field, and also go against the requirements of today's social development. The newly developed solar cells—including organic solar cells (OSCs), perovskite solar cells (PSCs), and dye‐sensitized solar cells (DSSCs)—that utilize organic semiconductor materials have garnered significant attention due to their potential benefits, such as low cost, mechanical flexibility, light weight, and compatibility with roll‐to‐roll printing techniques. Another significant application of solar energy conversion is photocatalytic technology, which can directly transform solar energy into chemical energy stored in chemical compounds [5, 6, 7, 8, 9, 10, 11, 12, 13, 14, 15, 16, 17, 18]. Furthermore, solar energy conversion‐based photothermal technology can be utilized to generate and harvest thermal energy through a controllable and sustainable method. As we know, organic semiconductor materials possess advantages such as tunable energy levels, extended absorption range, and controllable photoelectric properties, which will meet the solar energy conversion requirements.

At present, metal complexes as functional materials have received considerable attention, and great progress has been made in energy storage and conversion [19, 20, 21]. These materials have an extreme degree of flexibility in structural design due to the diversity of organic ligands and metal centers, making it possible to decorate the metal complex with desired functionality. For example, metal complexes with long‐lived triplet excitons would help contribute to the charge generation in the OSCs [22]. Then the interface layers with the coordination function of metal complexes can enhance the charge extraction and passivation used in the PSCs [23]. What's more, the metal complex dyes can act as light absorbers to absorb photons to produce electrons in DSSCs [24]. In photocatalysis, metal complexes are attractive as photosensitizers or catalysts because of their tunable structures, diverse redox properties, and easy mechanistic studies. In particular, their redox behaviors can be precisely tailored by strategically coordinating metal centers with various synthetically available ligands, aligning with the thermodynamic demands needed for catalytic reactions [25]. For photothermal applications, metal complexes are expected to demonstrate robust absorption capabilities, particularly in the near‐infrared region, due to the distinctive charge transfer pathways between the ligand and the metal center [26]. Consequently, the photo‐to‐thermal conversion efficiency and fabrication cost can be significantly improved through strategic molecular design and the use of cost‐effective metal atoms. The use of functional metal complexes for solar energy conversion applications presents numerous new opportunities and continues to be a key focus of both current and future research.

This review provides a concise overview of the field, emphasizing the unique fundamental and practical importance of metal complex‐based functional materials in photovoltaics (such as OSCs, PSCs, DSSCs), photocatalysis (such as H_2_ production, CO_2_ reduction, and H_2_O_2_ production), and photothermal (such as water treatment and seawater desalination) applications in the last decade. We will discuss the different material categories, underlying principles of operation, and identified performance improvement techniques, with the aim of providing a comprehensive view of this rapidly evolving field. Finally, we will put forward an outlook on the future potential of metal complexes in highly efficient solar energy conversion technologies. It should be pointed out that porous metallated organic semiconductors such as conjugated microporous polymers (CMPs), covalent‐organic frameworks (COFs) and metal‐organic frameworks (MOFs) have also been developed for solar energy conversion [27, 28, 29, 30, 31, 32, 33, 34, 35, 36, 37, 38, 39, 40, 41], but those results are not included in this review, and interested readers can refer to the recent literature references [27, 28, 35, 41].

## 2 Metal Complexes for Solar–to–Electrical Energy Conversion

Photovoltaic technology is a clean and pollution‐free method for converting sunlight into electrical energy. Currently, commercial photovoltaic technology still primarily uses silicon and inorganic semiconductor materials to fabricate photovoltaic modules, which have a lifespan of several decades. However, the high cost and the difficulty in producing flexible, lightweight, semi‐transparent, and roll‐to‐roll photovoltaic modules limit their applications. In view of this, new photovoltaic technologies have been developed rapidly. These new technologies not only involve lower cost but also possess many other advantages, such as being lightweight, flexible, and easy to process. Among these photovoltaic technologies, OSCs, PSCs, and DSSCs have been developed particularly quickly. For example, OSCs have now achieved efficiencies exceeding 20% for both single junction and tandem cells [42, 43, 44]; PSCs have surpassed certified 26% efficiency in single junction [45]; and DSSCs have exceeded 15% efficiency [46], demonstrating their significant potential. These photovoltaic technologies utilize organic semiconductor materials as active layers, interface layers, passivators, sensitizers, or other functional layers, taking full advantage of the various optoelectronic properties of organic semiconductors. Among these organic semiconductors, metal complex‐based semiconductor materials impart additional special properties. In this part, we will provide a survey and understanding of the application of metal complexes in photovoltaic conversion. Here, we will also pay particular attention to the functions of metals installed into the organic semiconductor materials.

### 2.1 Metal Complexes in Organic Solar Cells

Different from conjugated organic materials, metal complex materials offer several distinct advantages. (i) Metal atoms facilitate electron transfer, thereby enhancing the electronic characteristics, (ii) metal centers encourage intersystem crossing from the lowest singlet (S_1_) to triplet (T_1_) excited states, leading to prolonged exciton lifetimes and longer diffusion lengths, and (iii) metal‐metal and/or metal‐ligand interactions influence intermolecular interactions and enable the modulation of material morphology [47]. The various advantages of these materials position metal‐based organic donors or photosensitizing molecules as a highly promising category of compounds for OSCs. This part will summarize the common metal complex materials used in OSCs.

#### 2.1.1 Metalloporphyrin‐Based Small Molecules

Porphyrin and its derivatives, well‐known as photosensitizers, are crucial in various biological, catalytic, and artificial photosynthesis applications. Recently, metalloporphyrin analogs have garnered increasing interest as electron donor and acceptor materials in bulk heterojunction (BHJ) OSCs thanks to several inherent characteristics: (1) a wide absorption spectrum coupled with high molar extinction coefficients, (2) adjustable electronic properties through the modifications of substituents at peripheral positions (*meso*‐ and *β*‐) and by altering the metal ions in the porphyrin core, (3) outstanding thermal stability, and (4) a strong ability to promote electron transfer reactions effectively [48].

The simple metalloporphyrin used in OSCs exhibited a low PCE due to the poor crystallinity and low charge mobility [49]. Then researchers modified the structure by inserting ethynylene substituted donor or acceptor units to form a donor (D)–acceptor (A) conjugated skeleton. This modification made the porphyrin materials attractive as donor components, as the highest occupied molecular orbital (HOMO) and the lowest unoccupied molecular orbital (LUMO) energy levels, along with other optoelectronic properties, can be easily tuned. A typical A–D–A structure was implemented to improve the intramolecular charge transfer (ICT) in porphyrins. Among the acceptor units, the highly electron‐deficient diketopyrrolopyrrole (DPP) was selected to link to the metalloporphyrin core through ethynylene bridges. The inclusion of the DPP unit effectively shifts the absorption spectrum to the longer wavelength and enhances the photovoltaic performance. In the early stage, metalloporphyrin with an A–D–A configuration still exhibited very low performance due to the inferior crystallinity and disordered surface morphology [50]. To address this, Peng et al. synthesized a small molecule based on metalloporphyrin–DPP, denoted as donor **1**, which incorporated 2‐ethylhexylthienyl ring into the porphyrin core [51]. The introduction of alkylthienyl groups resulted in a deeper *E*_HOMO_ and facilitated a transition from *J*‐ to *H*‐aggregation upon treatment with pyridine additives and thermal annealing. Grazing‐incidence X‐ray diffraction (GIXD) analysis revealed that the addition of pyridine enhanced crystallinity in the (100) direction. Transmission electron microscopy (TEM) images showed that pyridine‐treated films exhibited improved phase separation, with characteristic domains of approximately 30 nm. Resonant soft X‐ray scattering (RSoXS) analysis revealed that CB+Py‐treated films displayed a wide peak at 0.0221 Å^−1^, indicating a phase separation distance of 28.4 nm. This led to the achievement of a high PCE of 8.08%, with a minimal energy loss of only 0.59 eV when paired with PC_71_BM. Furthermore, it was observed that incorporating a central Zn(II) ion into donor **1** enhanced the FF and *J*_sc_ of the devices, likely due to improvements in the morphology. Following this, the same group synthesized several other metalloporphyrin–DPP donors with varying alkyl chains on the thiophene. Notably, donors **2** and **3** demonstrated impressive PCEs of 9.06% and 8.24%, respectively, due to their well‐ordered D–A domain morphology, which increased crystallinity and improved both *J*_sc_ and FF values [52]. Subsequently, they synthesized another metalloporphyrin–DPP donor **4**, which demonstrated PCEs of over 19% in OPV/perovskite hybrid cells and 17% in all‐small‐molecule solar cells, representing the highest value at that time [53]. Peng et al. reported another metalloporphyrin–DPP donor **5** through introducing benzothiophene groups into the porphyrin core [54]. This resulted in a PCE of 9.08% and a *V*_oc_ of 0.8 V in **5**:PC_61_BM devices. Langa et al. developed A–π–D–π–A type small molecules **6**, which incorporate 3‐ethylrhodanine linked at the *meso* positions by ethynylene linkers and interconnected through two thienylenevinylene units [55]. When treated with pyridine/THF and a CuSCN hole transporting layer, these devices achieved a PCE of 7.24%. In order to investigate the impact of metal incorporation, a series of porphyrin donors were synthesized, featuring no metal, as well as palladium (Pd) or platinum (Pt) at the central core. So et al. found that **8** and **9**, incorporating Pd and Pt, exhibited deeper HOMO levels compared to **7**, which lacked metal cores [56]. Devices based on **8** and **9** demonstrated PCEs of 8.09 and 7.31%, respectively, higher than that of **7** (2.51%). Moreover, these devices delivered promising PCEs of 16.54 and 16.74% under a 300 lux LED source. Langa et al. synthesized a gold‐based porphyrin acceptor **10** [57]. This molecule exhibited strong electron transport properties due to the efficient ICT between Au‐Porphyrin and acceptor terminals, achieving a PCE of 9.24%, accompanied by a low energy loss of 0.56 eV after being processed by solvent vapor annealing (SVA).

**FIGURE 1:**
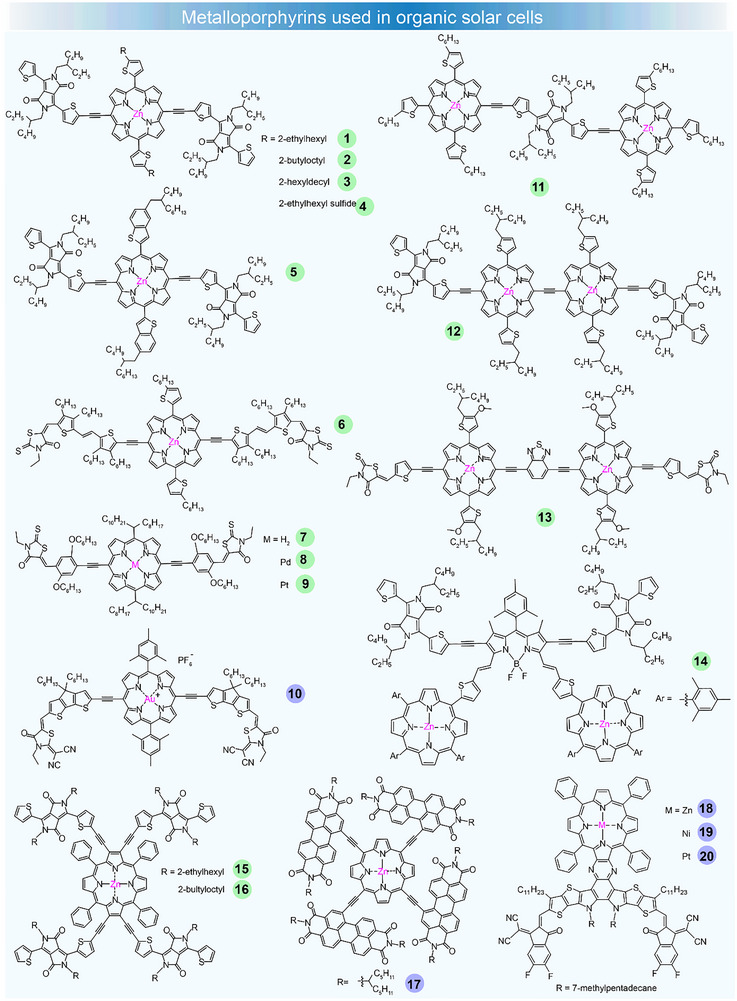
Chemical structures of metalloporphyrins used in OSCs. Green and purple dots represent metal complexes used as donor and acceptor materials in OSCs, respectively.

The limited absorption of porphyrin‐based donors below 850 nm has hindered their photon utilization and overall performance. To address this, Langa et al. designed and synthesized a porphyrin dimer **11** bridged with DPP‐ethynylene and terminated with thiophene groups [58]. Devices incorporating **11**:PC_71_BM demonstrated a good PCE of 8.03%, characterized by minimal energy loss and a high external quantum efficiency (EQE) exceeding 900 nm. Peng et al. also reported another porphyrin dimer **12**, where two porphyrin units were connected by a single ethynylene bridge, with two DPP units attached as arms [59]. The optimized device **12**:PC_61_BM showed an extended photoresponse up to 1000 nm, obtaining a remarkable PCE of 8.45% and a *J*_sc_ of 19.65 mA cm^−2^. After that, the same group modified the structure by incorporating benzothiadiazole electron‐withdrawing units at the center, resulting in a new dimer **13** [60]. Compound **13** exhibited an increased molar extinction coefficient at the NIR absorption peak and a reduced HOMO level. The **13**‐based device, processed with pyridine additive and SVA methods, reached an encouraging PCE of 10.02%, with improved *J*_sc_ and *V*_oc_. GIXD results showed that films treated with TA + SVA and SVA showed better crystallinity compared to those treated with other methods. Gros et al. reported **14**, which featured a BODIPY core encircled by two DPP and two porphyrin units (Figure 1) [61]. **14** showed a PCE of 8.98%, attributed to improved exciton dissociation and charge transport. Recently, Peng et al. introduced two porphyrin donors **15** and **16**, each with four DPP units attached to the *β*‐positions via ethynyl connections [62]. These donors exhibited an “X”‐shaped structure, similar energy levels, and absorption profiles. Optimized devices with **15** and **16** showed PCEs of 10.19 and 10.99%, respectively. Li et al. synthesized a star‐shaped electron acceptor **17** based on porphyrin [63], which demonstrated favorable energy alignment, high electron mobility, and complementary absorption with the donor polymer, achieving a PCE of 7.4%. Recently, Chen et al. reported a set of acceptors **18**–**20** with metalloporphyrin complexes as central units [64]. The alteration of the central metal leads to significant changes in the spatial arrangement of HOMOs and LUMOs, effectively influencing the energy levels, light‐harvesting efficiency, and dipole moments of SMAs. Importantly, **20**, which contains Pt, appears to effectively reduce the non‐radiative recombination by optimizing the luminescent characteristics of acceptors, offering a valuable example for linking up the energy loss in OSCs. Surprisingly, **20**‐based ternary OSCs achieved an excellent PCE exceeding 20%, highlighting the promising potential of metal complexes in developing photovoltaic materials. The detailed parameters are listed in Table 1.

**TABLE 1:** Summary of the photovoltaic parameters of OSCs using metal complex materials.

Material	*E*_g_^opt^ (eV)	HOMO/LUMO (eV]	*V*_oc_ (V)	*J*_sc_ (mA cm^−2^)	FF (%)	PCE (%)	Refs.
**1**	1.37	−5.14/−3.76	0.78	16.76	61.80	8.08	[51]
**2**	1.37	−5.23/−3.86	0.73	19.58	63.38	8.96	[52]
**3**	1.37	−5.24/−3.87	0.73	17.23	65.54	8.24	[52]
**4**	1.38	−5.20/−3.82	0.79	17.35	57.10	7.83	[53]
**5**	1.40	−5.17/−3.75	0.80	16.82	67.54	9.08	[54]
**6**	1.44	−5.30/−3.56	0.94	11.67	66.00	7.24	[55]
**7**	1.65	−5.55/−3.90	0.67	7.03	51.20	2.51	[56]
**8**	1.77	−5.65/−3.88	0.94	12.82	67.00	8.09	[56]
**9**	1.85	−5.71/−3.86	0.98	11.18	66.10	7.31	[56]
**10**	1.39	−5.80/−4.21	0.83	17.36	63.00	9.24	[57]
**11**	1.38	−5.44/−3.72	0.82	14.19	69.00	8.03	[58]
**12**	1.28	−5.13/−3.85	0.65	19.65	66.15	8.45	[59]
**13**	1.40	−5.17/−3.77	0.84	17.49	66.79	9.81	[60]
**14**	1.44	−5.36/−3.79	0.95	14.32	67.00	8.98	[61]
**15**	1.60	−5.40/−3.80	0.78	19.32	66.97	10.19	[62]
**16**	1.60	−5.40/−3.80	0.79	20.24	68.74	10.99	[62]
**17**	1.48	−5.46/−3.68	0.78	14.50	66.00	7.40	[63]
**20**	/	−5.58/−3.59	0.86	28.81	81.04	20.10	[64]
**22**	2.06	−5.10/−3.00	0.83	7.10	40.00	2.37	[65]
**23**	1.93	−5.03/−3.13	0.80	7.15	41.00	2.34	[65]
**28**	1.91	−5.27/−3.57	0.91	4.88	33.47	1.56	[66]
**29**	1.69	−5.37/−3.49	0.92	5.89	29.09	1.59	[66]
**30**	1.79	−5.00/−3.20	0.82	11.90	57.00	5.90	[67]
**31**	1.47	−5.55/−3.61	0.89	26.90	80.62	19.30	[68]
**32**	/	−5.67/−3.87	0.88	27.15	79.79	19.24	[69]
**33**	/	−5.42/−3.33	0.92	8.91	47.00	3.81	[70]
**34**	1.78	−5.45/−3.67	0.79	11.83	32.53	3.05	[71]
**35**	1.60	−5.23/−3.63	0.63	14.48	40.14	3.68	[72]
**36**	2.32	−5.24/−2.93	0.95	8.92	35.92	3.04	[73]
**37**	2.06	−5.31/−3.25	0.96	16.67	53.78	8.62	[73]
**38**	1.88	−5.34/−3.46	0.73	23.55	65.02	11.12	[74]
**39**	2.16	−5.42/−3.26	0.87	27.98	75.00	18.37	[75]
**45**	1.58	−5.34/−3.64	0.94	10.71	64.00	6.44	[76]
**46**	1.49	−5.43/−3.92	0.88	11.86	55.00	5.74	[77]
**47**	1.42	−5.39/−4.02	0.88	12.66	62.00	6.89	[77]
**48**	1.85	−5.37/−3.14	0.82	13.10	37.00	4.10	[78]
**49**	1.81	−5.14/−3.33	0.78	9.61	49.30	4.13	[79]
**52**	1.54	−528/−3.45	0.80	16.21	65.01	8.45	[80]
**56**	1.94	−5.53/−3.51	0.81	26.45	76.30	16.35	[81]
**58**	1.81	−5.60/−3.79	0.90	27.56	77.60	19.24	[82]
**62**	1.64	−5.22/−3.13	075	16.00	67.00	8.04	[83]
**66**	1.82	−5.55/−3.60	0.84	26.12	78.41	17.24	[84]
**71**	2.05	−5.69/−3.64	0.84	26.16	75.33	16.71	[85]
**79**	1.63	−5.30/−3.31	0.77	16.10	70.00	8.60	[86]
**80**	2.37	−5.02/−2.65	0.83	27.77	79.67	18.54	[87]
**81**	/	−5.65/−2.31	0.97	25.17	74.70	18.24	[88]
**82**	/	−5.28/−2.56	0.96	24.79	76.53	18.29	[89]
**83**	/	−5.31/−2.65	0.96	24.69	74.39	17.63	[90]
**84**	/	−5.31/−2.59	0.97	25.18	75.88	18.45	[90]
**85**	/	/	0.931	26.81	80.60	20.12	[90]

In this part, the metalloporphyrin‐based small molecules showed great progress in OSCs. However, the traditional zinc porphyrin donors still presented modest efficiency. To overcome these challenges, substituting zinc with platinum or other transition metals could potentially improve the performance by facilitating the formation of triplet excited states with longer lifetimes, thus enhancing charge separation and transport [91]. Overall, the incorporation of metal atoms into porphyrin structures holds considerable promise, offering versatile optoelectronic properties that merit further exploration.

#### 2.1.2 Other Small‐Molecular Metal (Pt, Ir, Ru, Fe) Complexes

Except for metalloporphyrin small molecular materials, there are other metal (Pt, Ir, Ru, Fe) complex‐based molecular materials used as donors or acceptors in OPV.

##### 2.1.2.1 Pt(II) Complexes

**FIGURE 2:**
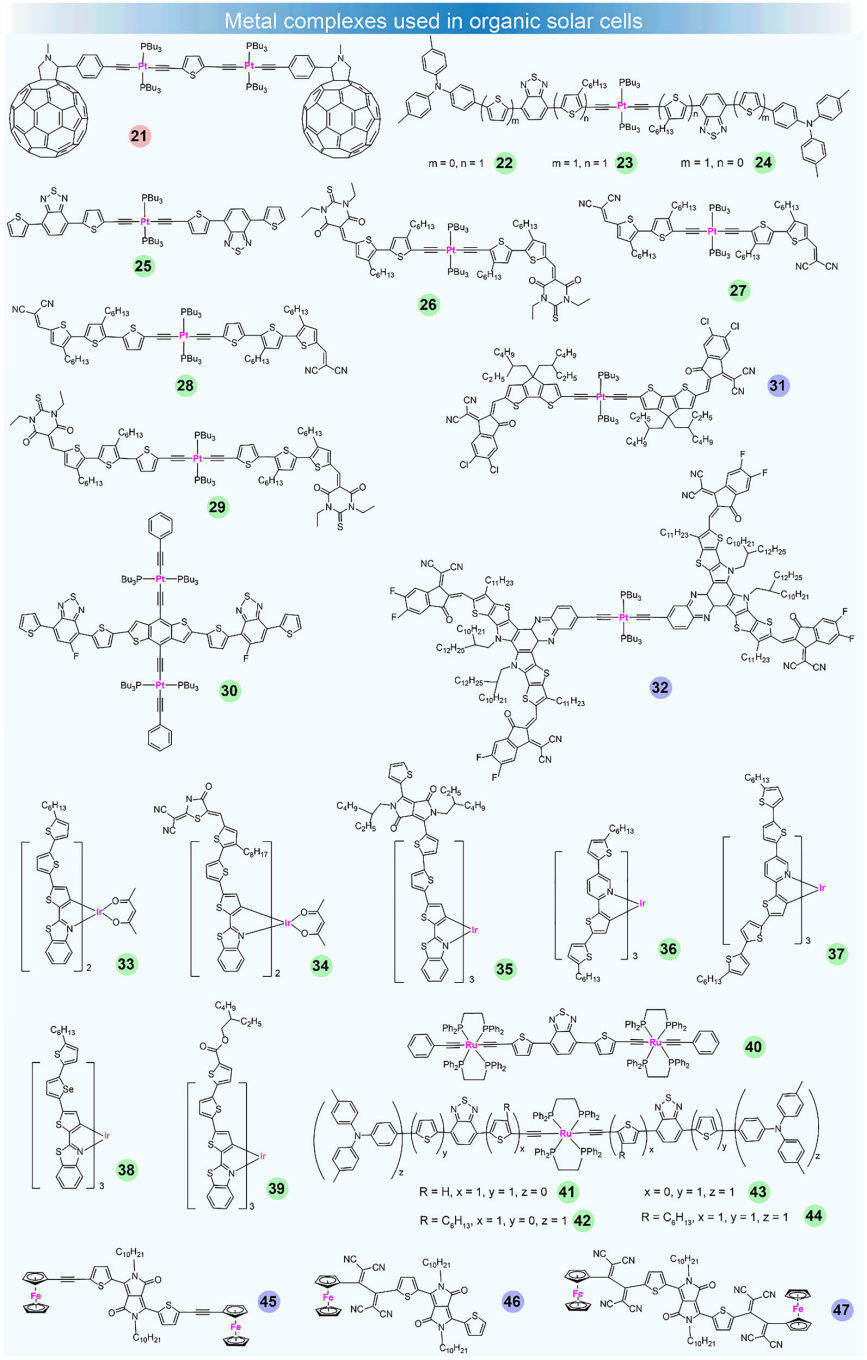
Chemical structures of metal complexes used in OSCs. Pink, green, and purple dots represent metal complexes used as active layer, donor, and acceptor materials in OSCs, respectively.

In 2007, Schanze et al. synthesized a D–A triad (**21**) by connecting a Pt‐acetylide oligomer to two C_60_‐based materials (Figure 2) [92]. Interestingly, four reversible redox waves appeared in the electrochemical scans, suggesting the enhanced photoinduced electron transfer (PET) efficiency and a reduced spatial separation between the D and A phases. They found that the triplet state in the triad is efficiently quenched through an intramolecular PET to C_60_. They fabricated OPV devices based on **21,** which achieved a very low PCE of 0.05%. Their results demonstrated the connection between the PET mechanism in the triad and the relatively high photovoltaic response exhibited by the material. After that, Wong et al. reported several Pt(II)–bis(aryleneethynylene) complexes **22**–**25** [65]. Among these complexes, **25** exhibited coplanarity of the di(thienyl)benzothiadiazole unit, which can extend the absorption to 600 nm. Their absorption data showed that triphenylamino groups with strong electron‐donating property can broaden and enhance the ICT transition. OPVs based on **22** and **23** achieved the highest performance, with PCE values ranging from 2.34 to 2.37%. Then they also reported another four metallated conjugated oligothiophenes **26**–**29** with two different accepting end groups [66]. These materials possessed fairly low‐lying HOMO energy levels, which align well with the LUMO energy level of the electron acceptor PC_70_BM. Moderate PCEs of 1.56% and 1.59% were achieved based on **28** and **29**, respectively, probably due to their better morphology and efficient charge transfer. Furthermore, Qin et al. synthesized a new Pt‐bis(acetylide) small molecule, **30**, characterized by a “roller‐wheel” geometry [67]. Compared to conventional Pt‐containing polymers with “dumbbell” shaped structures, **30** exhibited enhanced crystallinity and stronger intermolecular π–π interactions. OSCs based on **30** demonstrated a PCE up to 5.9%. Recently, Wong's group synthesized a novel metal complex acceptor (MCA), **31**, and selectively incorporated it into the upper Y6 host layer of pseudo‐planar heterojunction (PPHJ) to regulate the morphology and fill the trap states [68]. Experiments revealed a strong chemical interaction between **31** and Y6, which can promote electron transfer. **31** can regulate the self‐aggregation and extend the exciton diffusion length of Y6, resulting in enhanced charge transport and reduced energetic disorder. OSCs based on PM6/L8‐BO:**31** achieved a champion PCE of 19.30% with high thermal stability and photostability. Their work introduces the concept of MCA and provides a practical and efficient method to enhance the efficiency and stability of OPVs. After that, Li et al. reported a novel Pt complex **32**, which features a platinum core connected to Y‐acceptor arms [69]. **32** exhibits distinct singlet and triplet states with microsecond lifetimes of 3.88 µs, much longer than the nanosecond lifetimes of Y‐acceptors without the metal. Incorporating **32** as a third component in ternary OSCs has resulted in a remarkable efficiency of 19.24%.

##### 2.1.2.2 Ir(III) Complexes

Unlike the four‐coordinate structure of Pt, due to the outer electronic configuration of the Ir atom, it generally forms six‐coordinated complex. As a result, its corresponding complexes have different spatial configurations and packing arrangements, which have a significant impact on the morphology of the active layer in OSCs. Tao et al. reported a cyclometallated Ir complex **33** used as a small molecular donor in OSCs for the first time [70]. They found that the complex **33** led to a notably extended exciton lifetime, enhanced optical absorption, and improved film morphology. OSCs utilizing the **33**:PC_71_BM blend achieved a maximum PCE of 3.81%. Based on this result, they further prepared devices based on PDCBT/**33**:PC_71_BM to push the PCE to 6.17% [93]. They also studied the effect of electron‐accepting end groups of the main ligands on the triplet state character. The results showed that the enlarged ligand conjugation length and amplified intraligand D–A interactions could induce less spin‐orbit coupling, resulting in noticeably weaker triplet emission and shorter triplet state lifetimes. For example, complexes **34** and **35** only achieved PCEs of 3.05% [71] and 3.41% [72], respectively, significantly lower than **33** without the electron‐accepting end groups (PCE of 5.36%). They concluded that a stronger triplet feature would aid in exciton dissociation, reduce charge recombination, and improve photovoltaic performance. Furthermore, they reported another octahedral homoleptic tris‐Ir(III) complexes **36** and **37** [73]. The small‐sized backbone of **36** exhibits a stronger triplet characteristic, with enhanced phosphorescence and a longer exciton lifetime, in comparison to **37**. OSCs based on **36** and **37** achieved PCEs of 3.04% and 8.62%, respectively. Recently, they reported two Ir(III) complex **38** [74] and **39** [75] by selenylation of the cyclometallated ligand and substitution with ethylhexyl carboxylic ester, respectively. Upon Se‐substitution, **38** demonstrated a bathochromic shift in absorbance, improved light‐harvesting ability, along with redshifted and reduced phosphorescence emission. OSCs based on **38**:Y6 exhibited a PCE of 11.12%. While in the PM6:L8‐BO:**39** device, a champion PCE of 18.37% was achieved due to the better miscibility between **39** and L8‐BO.

##### 2.1.2.3 Ru(II) and Fe(II) Complexes

Apart from the Pt(II) and Ir(III) complex, Ru(II) and Fe(II) complex can also be used in OSCs. Colombo et al. reported dinuclear acetylide donor complex **40**, where two Ru atoms are bridged by a 2,1,3‐benzothiadiazole (BTD) unit, with 2,5‐thienyl groups attached on both sides [94]. However, owing to the strong phase segregation between **40** and PCBM, a very low PCE of 0.1% was achieved in the OSCs. Wong et al. reported a set of multichromophoric, Ru(II)‐bis(aryleneethynylene) complexes **41**–**44**, incorporating triphenylamine and/or thiophene as the donor units and BTD as the acceptor units [95]. The highest PCE of 0.66% was obtained for **41**, marking the best performance for Ru(II)‐containing OSCs. Sharma et al. reported a Fc‐containing donor **45**, which featured a thiophene‐flanked DPP core and a ferrocene unit capped at the ends, connected via an ethynyl bridge [76]. The electron‐withdrawing characteristics of DPP can induce ICT from ferrocene to DPP, while the ethynyl bridge lowers the HOMO energy level of **45**. Additionally, the ethynyl bridge can promote a more planar conformation in **45** due to its cylindrical‐like π‐electron density, which in turn enhances intermolecular π–π stacking and supports more efficient charge transport between molecules. The best PCE of 6.44% was achieved after optimizing the devices by using 3 vol% DIO/THF processed solvent and thermally annealing. After that, the same group synthesized another ferrocenyl tetracyanobutadiene derivative of diketopyrrolopyrroles **46** and **47** as efficient acceptors [77]. After the optimization of the OSCs, the thermally annealed PSCs based on **46** and **47** showed an overall PCE of 6.44% and 6.89%, respectively.

#### 2.1.3 Metal‐Containing Polymer Materials

Conjugated polymers with metal atoms also show distinctive properties, as the metal atoms can enhance spin–orbit coupling and promote intersystem crossing between singlet and triplet states. This, in turn, promotes the formation of triplet excitons, which have extended the exciton lifetimes and allow for greater exciton diffusion lengths [96]. Besides, the incorporation of metal complexes into the conjugated polymers can also regulate the aggregation and crystallinity, which will affect the film formation and morphology of the polymer [84]. Among various metals, Pt and Ir are most widely used in polymer donors. When Pt ions form bonds with alkyne units to create a linear polymer chain, the interaction between *d*‐orbitals of Pt and the *p*‐orbitals of the alkyne promotes the delocalization of π‐electrons, thereby improving charge transport within the polymer chain. Research has also demonstrated that charge transport occurs within the Pt‐acetylide complexes [97, 98].

**FIGURE 3:**
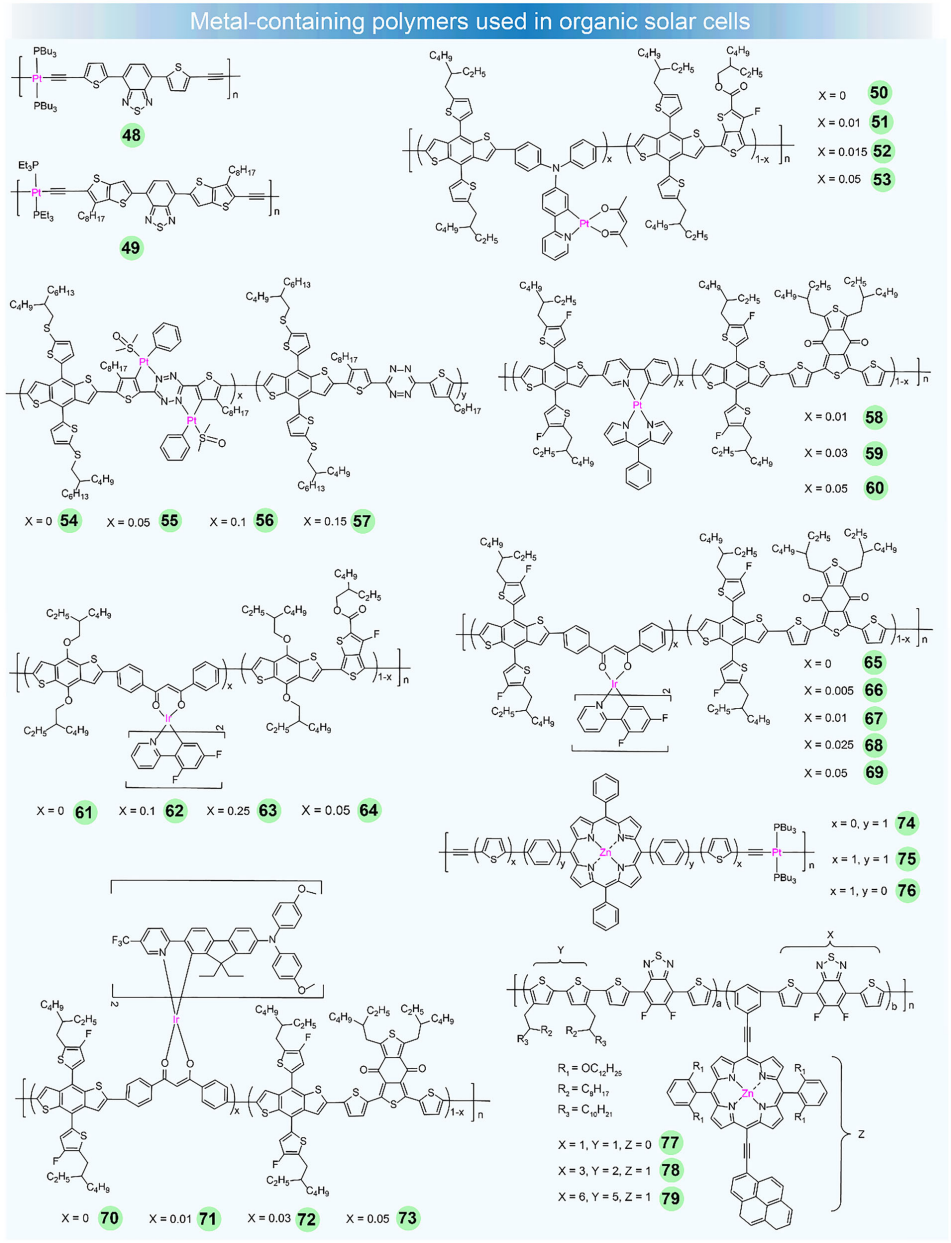
Chemical structures of metal‐containing polymers as donors used in OSCs.

**FIGURE 4:**
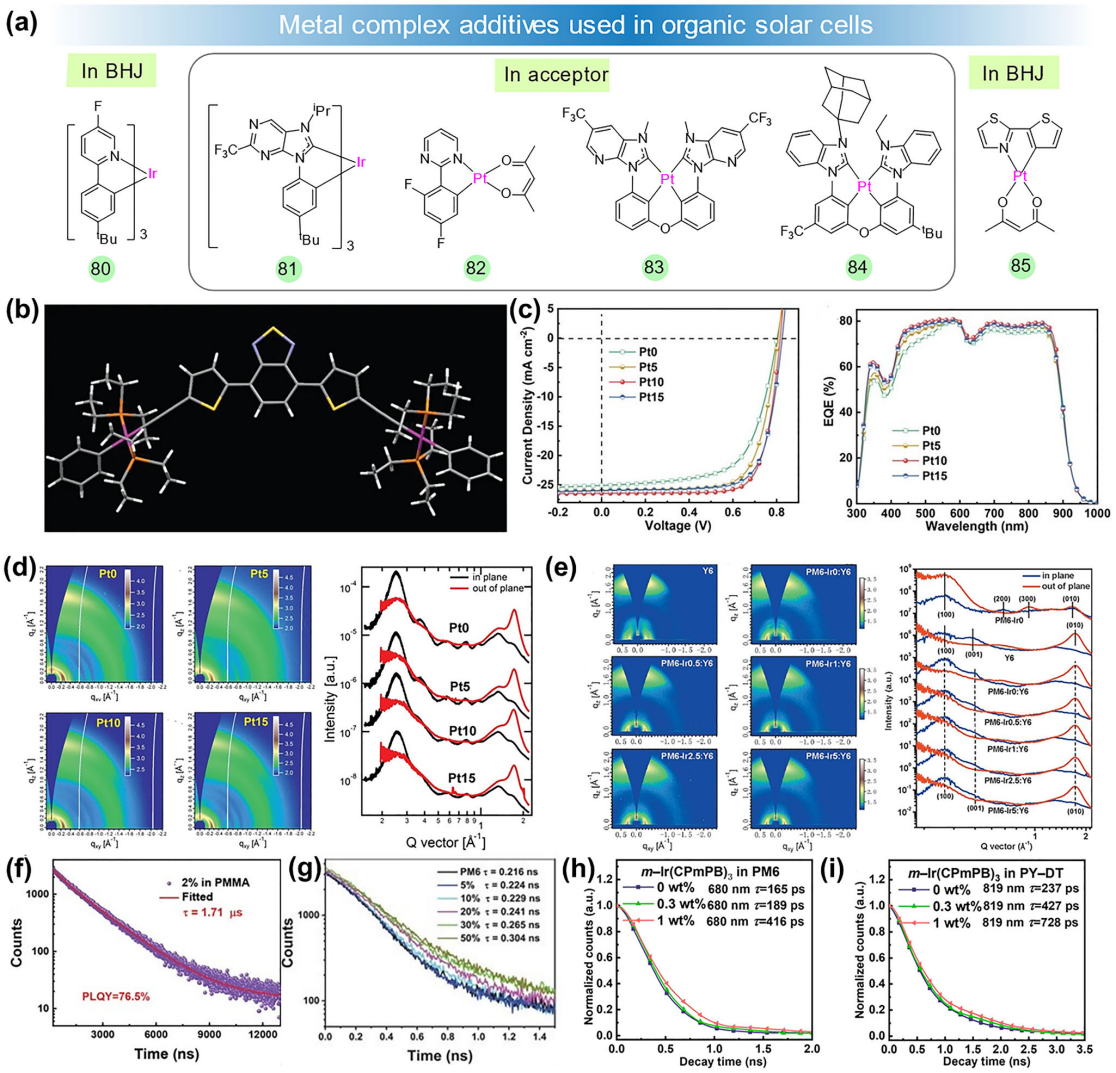
(a) Chemical structures of metal complex additives **80**–**85** used in OSCs. (b) A 3D representation of the crystal structure of model complex **48**. (c) *J*–*V* and EQE curves of the Pt‐based polymer donors **54**–**57**. (d) 2D‐GIWXAS patterns and IP and OOP line‐cut profiles of **54**–**57**. (e) 2D‐GIWAXS profiles and IP/OOP line‐cuts for the pure and blend films. (f) Transient decay of **80** in PMMA film. (g) TRPL spectra of blend films at varying ratios of **80**. TRPL spectra of (h) PM6 and (i) PY‐DT films with different ratios of **81** under 375 nm light excitation. (b) Reproduced with permission [78]. Copyright 2007, Springer Nature. (d) Reproduced with permission [81]. Copyright 2019, Wiley‐VCH. (e) Reproduced with permission [84]. Copyright 2020, Wiley‐VCH. (f) Reproduced with permission [87]. Copyright 2024, Wiley‐VCH. (h,i) Reproduced with permission [88]. Copyright 2024, Elsevier Science Publishers.

In the early stage, triplet states play a role in charge separation, contributing to charge transfer. However, the wide bandgap of polymers limits their ability to absorb additional photons, which in turn restricts the photocurrent quantum yield and hinders the PCE of devices [99]. In a pioneering work reported by Wong et al., they synthesized a narrow‐bandgap D–A polymer **48**, featuring 4,7‐di‐20‐thienyl‐2,1,3‐benzothiadiazole as its core structure (Figure 3) [78]. They meticulously characterized the structure of **48,** using X‐ray crystal structure analysis of its monomer (Figure 4b). The polymer displayed an *E*_g_ of 1.85 eV, due to the strong electron‐deficient nature of benzothiadiazole group. Unlike previous Pt polymer donors, charge transfer excited state, rather than triplet state, was primarily responsible for the efficient photoinduced charge separation for **48**. After optimizing the devices, BHJ solar cells based on **48**:PCBM achieved an impressive PCE of 4.1%, with a *V*_oc_ of 0.82 V, *J*_sc_ of 13.1 mA cm^−2^, and FF of 0.37. Their work highlighted the potential of metallated conjugated polymers in enhancing the efficiency of photovoltaic devices. Jean et al. reported amorphous Pt(II) polymers incorporating a rigid thieno[3,2‐b]thiophene‐2,1,3‐benzothiodiazole spacer, **49** [79]. Owing to the ICT between D and A units, combined with the steric hindrance from the bulky Pt(Et_3_)_2_ group, **49** exhibited a field effect hole mobility up to 1.0 × 10^−2^ cm^2^ V^−1^ s^−1^. The PCE of **49** reached 4.13%, with a *V*_oc_ of 0.787 V, *J*_sc_ of 9.61 mA cm^−2^, and FF of 0.52, resulting from large phase separation.

Subsequently, Huang's group introduced Pt(II) complexes into the widely known PTB7‐Th polymer, creating metallopolymers **50**–**53** [80]. The polymer **52** showed an improved PCE of 8.45%, surpassing the control copolymer's PCE (7.92%). The enhancement was ascribed to the increased hole mobility, reduced bimolecular recombination, and more efficient charge separation process. In 2019, Peng et al. reported a novel strategy involving platinum(II) complexation to regulate the crystallinity and molecular packing order of polymer donors **54**–**57** [81]. The introduction of Pt(Ph)_2_(DMSO)_2_ to the polymer reduced the aggregation by increasing the steric hindrance. This strategy enabled preferable morphology with more ideal phase separation, which contributed to the enhanced exciton diffusion and dissociation. Devices based on **56** achieved the champion PCE of 16.35%, with a *J*_sc_ of 26.45 mA cm^−2^, an FF of 76.3%, and a *V*_oc_ of 0.81 V (Figure 4c). The external EQE of **56‐**based devices across the entire absorption range was improved, reflecting better exciton dissociation and charge collection. The GIWAXS data showed a gradual reduction in the coherent length (*L*_C_) from 131 to 95 Å, as well as a decrease in diffraction intensity at (200) and (300) peaks, with increasing the content of Pt complex. From **54** to **57**, the π–π stacking distance *L*_C_ reduced from 36 to 26 Å (Figure 4d), suggesting that Pt‐complexation enhanced the steric hindrance, which in turn decreased the crystallinity and aggregation strength, leading to a more ordered packing of the resulting polymers. Very recently, Wong et al. synthesized three Pt‐complex‐derived terpolymer donors **58**–**60** [82]. By adding a trace amount of Pt complex, the metallated terpolymers exhibit high triplet energy (*E*_T1_) and small singlet–triplet energy gap (Δ*E*_ST_). As a result, enhanced exciton lifetime and diffusion length were obtained as well as suppressed non‐radiative recombination. The **58**‐based devices achieved champion PCEs of 18.54% (certified as 18.32%) and 19.24% in the binary and ternary devices, the highest values reported to date.

Iridium metal‐based polymers have also attracted wide attention as the polymer donors. In 2005, Huang et al. reported a set of Ir‐containing terpolymers **61**–**64** by incorporating a low concentration of Ir(III) complex into the polymer backbone [83]. Increasing the Ir complex concentration led to a significant enhancement in PCE due to the participation of triplet state effects, although excessive amounts of Ir complexes negatively affected the PCE. The OSC based on **62** gave the best PCE of 8.04%, with *V*_oc_ = 0.75 V, *J*_sc_ = 16.0 mA cm^−2^, and FF = 0.67. Their work demonstrated that incorporating the triplet content into existing high‐efficiency materials can afford an extra approach to lead to a breakthrough in the PCE of OSCs. Inspired by this approach, Min et al. reported a series of terpolymers **65**–**69** by incorporating different concentrations of Ir complexes into the polymer backbone of PM6 [84]. GIWXS analysis showed a weakening of the (001) peak at q_xy_ = 0.44 Å^−1^ in the IP direction (for Y6) for PM6‐Ir*_x_*‐based blend films, indicating reduced molecular stacking order upon Ir incorporation (Figure 4e). What's more, a distinct (010) diffraction peak at q_z_ = 1.736 Å^−1^ appeared in the **65**:Y6 blend, with diminished intensity in the Ir‐containing films. Their work suggested that enhancing the degree of Ir complexation not only modified the molecular aggregation of polymer donors, but also slightly reduced the crystallinity and aggregation strength of Y6 molecules in the respective blends. Consequently, the **67**:Y6 blend exhibited the highest PCE of 17.24%, with a certified PCE of 16.70%, outperforming the control device based on **65**:Y6 blend (PCE of 15.39%). Recently, Wong et al. also reported a series of metallated terpolymer donors **70**–**73** by incorporating a novel Ir complex into the PM6 backbone [85]. The OSCs based on **71**:Y6 exhibited the best PCE of 16.71%, with *J*_sc_ of 26.16 mA cm^−2^, *V*_oc_ of 0.848 V, and FF of 75.33%. They demonstrated that adding even a small quantity of Ir complex could improve the photon absorption, triplet exciton generation, charge mobility, and also optimize the morphology of the active layer. Three platinum(II) polyyne polymers containing zinc(II) porphyrinate **74**–**76** were synthesized to study the photoelectric properties [100]. Among them, polymer **76** exhibited more accentuated conjugation in the π‐system, which can be ascribed to the more favorable dihedral angles between the thiophene and porphyrin units. The metallopolymer **76** showed the highest PCE of 1.04%. Hsu et al. demonstrated a complementary light‐harvesting unit by adding a porphyrin‐pyrene pendant to the polymer backbone **77**–**79**, enabling it to effectively capture a broader range of the solar light spectrum [86]. Besides, the presence of two 2,6‐bis(dodecyloxy)phenyl substituents on the porphyrin unit effectively supressed the extent of aggregation, leading to a PCE of 8.6% under 5 vol% of 1‐chloronaphathalene additive.

#### 2.1.4 Metal Complexes as Additives

Apart from the metallopolymers, metal complex small molecules can also function as solid additives or third components in the ternary devices. Different from the above metal complex small molecules used in the active layers (donors or acceptors), these types of metal complex molecules can function as morphology regulators. What's more, these metal‐containing small molecules, with their triplet‐state characteristics, can enhance the exciton lifetime and diffusion length, thereby improving the charge transport. Wong et al. reported a spatially stereostructured complex **80**, and demonstrated its effectiveness as an additive in OSCs (Figure 4a) [87]. In a PMMA film, the lifetime of **80** was measured at 1.71 µs, accompanied by a high photoluminescent quantum yield (PLQY) of 76.54%, showcasing its strong and efficient phosphorescent emission (Figure 4f). The exciton lifetime in PM6:**80** blends was found to increase with increasing the ratio of **80** compared to pure PM6, suggesting that **80**, as a photosensitizer, can extend the exciton lifetime in PM6 (Figure 4g). When 10% of **80** was introduced into the device processed with non‐halogenated solvent o‐xylene, the PCE reached an impressive value of 18.54%, surpassing the control devices (17.41%). Their work demonstrated a successful approach for enhancing exciton lifetime and modifying the morphology of the active layer by incorporating metal complex additives. Inspired by this, the same group synthesized another triplet material **81,** which was used as an additive to fabricate layer–by–layer (LbL) OSCs [88]. The exciton lifetimes in PM6 and PY–DT films were enhanced, increasing from 165 to 416 ps and from 237 to 728 ps, respectively, upon the addition of **81**, suggesting efficient energy transfer from **81** to PM6 or PY–DT (Figure 4h,i). The LBL devices achieved an increased PCE of 18.24% from 17.32% with the incorporation of 0.3 wt% **81** into the PY–DT layer, ascribed to the efficient energy transfer, extended exciton lifetime, and enhanced exciton diffusion distance in the active layer. The same group also reported the use of other metal complex in OSCs. For example, they reported a Pt complex **82** as an energy donor additive for LBL OSCs [89]. The PBQ_x_‐TCl/PY‐DT devices achieved a high PCE of 18.29% when **82** was incorporated into the PY‐DT layer, benefiting from energy transfer between **82** and both donor and acceptor. Another two new tetradentate platinum(II) bis‐carbene complexes **83** and **84,** were synthesized as triplet additives for LbL all‐polymer solar cells [90]. Compared to **83**, **84,** which featured a bulky adamantyl group, supressed aggragation in the active layers. PBQ_x_‐TCl/PY‐DT devices with 0.2 wt% of **84** in the blend exhibited a PCE of 18.45%, attributed to the increased exciton yield and lifetime in the active layers. Very recently, Gao et al. reported an organometallic platinum complex **85** [101]. The addition of **85** into the D18‐Cl:L8‐BO system reduced the energy disorder, trap density, and non‐radiative recombination and enhanced the exciton binding energy, which is conducive to prolonging the exciton diffusion length. In the OSCs, high PCEs of 20.12% and 18.84% were obtained for the active layer thickness of 100 and 300 nm, respectively. The detailed parameters are listed in Table 1.

In conclusion, the benefits of metal complex‐based organic conjugated materials in OSCs include: 1) triplet‐state characteristics that can extend the diffusion length and exciton lifetime; 2) the ability of metal complex to regulate polymer aggregation, promoting suitable phase separation in the morphology; and 3) their potential as additives or photosensitizers to enhance light absorption or facilitate energy transfer to the host materials. However, according to the structure–activity relationships in the application of OSCs, several pertinent suggestions can be concluded as follows: first, trace amount of Pt or Ir complexes in the copolymerization help enhance the molecular crystallinity and reduce the aggregation. Second, specific luminescent metal complexes present not only in the backbone or as additives can also improve the light‐emitting efficiency of the active layer, thus reducing the non‐radiative recombination losses. Third, some metal complexes as the building units with high dipole moment and dielectric constant can be used to construct efficient acceptors, which may regulate the exciton dynamics process. Though some progresses have been made in using different metal complexes in OSCs, further in‐depth studies are needed to fully understand the underlying mechanisms, such as up‐conversion strategies and the triplet exciton transfer process.

### 2.2 Metal Complexes in Perovskite Solar Cells

Metal complex materials can also be utilized in the PSCs as multifunctional interface layers. Though the function of these materials was mainly focused on the functional groups attached to the metal, the metal atoms themselves still play a crucial role in electron transfer, facilitated by the electron‐rich and electron‐delocalizable nature of the metal units. Among the metal complexes, derivatives of ferrocene and copper phthalocyanine are particularly valuable due to their strong electron transfer ability and stability. This section will explore the use of metal complex materials based on ferrocene and copper phthalocyanine, highlighting their application in the PSCs.

#### 2.2.1 Ferrocene Derivatives

**FIGURE 5:**
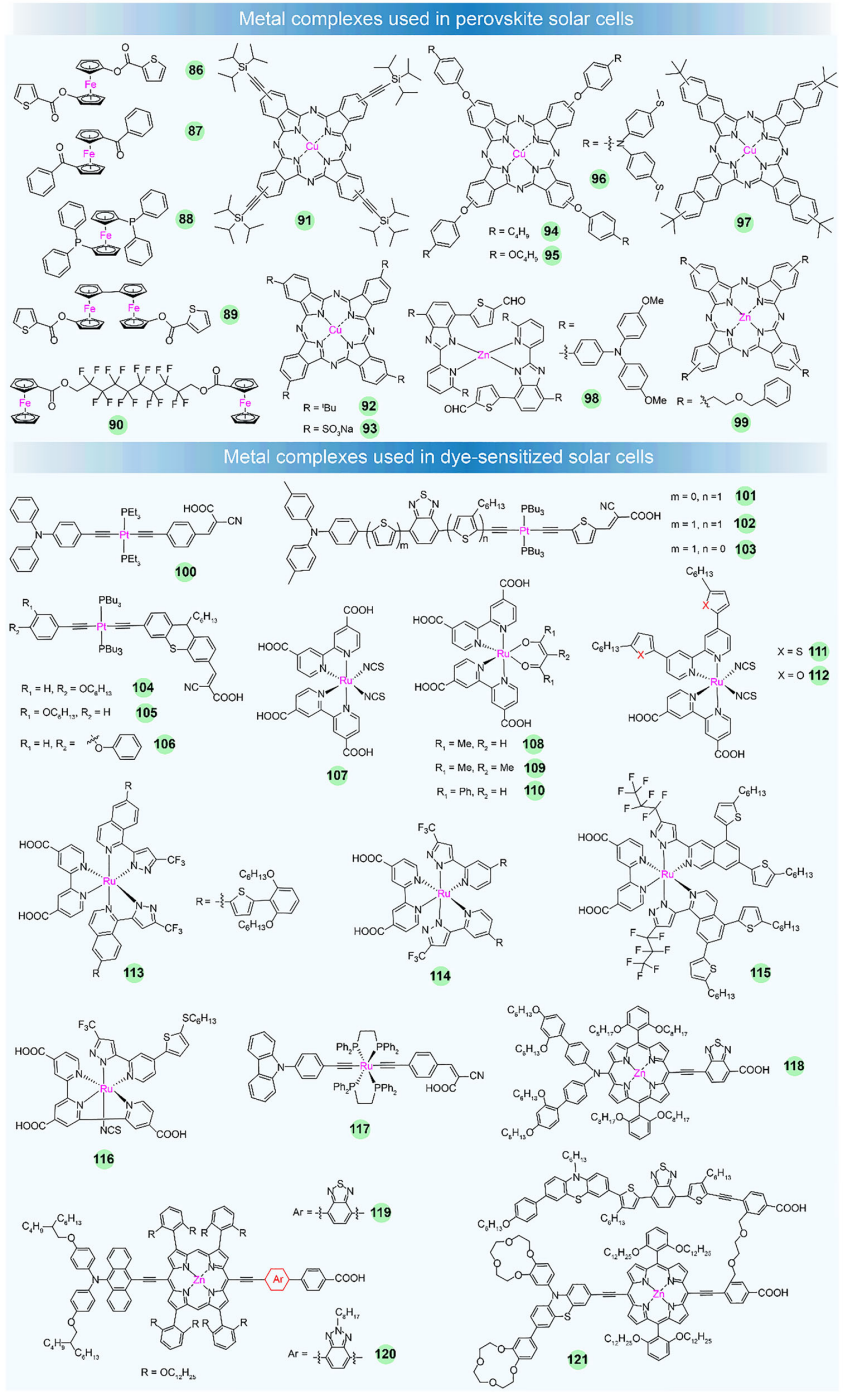
Chemical structures of metal complexes used in PSCs and DSSCs.

Recent studies have shown that inverted PSC devices exhibited improved stability and extended lifetimes, primarily owing to the application of undoped hole transport layers and the development of highly crystalline perovskite films [102, 103, 104]. Despite these advancements, the operational lifespan of inverted PSCs remains significantly shorter than that of commercially available silicon solar cells, which typically last for over 25 years [105, 106]. In 2022, Zhu et al. introduced a novel organometallic interface layer, ferrocenyl‐bis‐thiophene‐2‐carboxylate **86** (Figure 5), which enhances the stability of PSCs by providing strong chemical Pb‐O binding and improving interfacial electron transfer via its ferrocene units [107]. Using X‐ray photoelectron spectroscopy (XPS), they studied that the binding energies of Pb 4*f*, I 3*d*, and N 1*s* core levels shifted slightly to higher values in **86**‐treated perovskite films compared to untreated samples, suggesting that the compound strengthens the interaction between anions and cations at the perovskite surface (Figure 4). Other experiments demonstrated that **86** can inhibit the migration of surface ions, resulting in a more consistent and stable surface composition. Stability test showed that **86**‐functionalized device preserved its initial PCE for the first 200 h, with a decline of less than 2% after over 1500 h. Furthermore, under the damp heat test (85°C and 85% RH), the **86**‐modified devices exhibited a T_95_ (time to 95% efficiency) of over 1000 h. Even under cycling thermal stress between −40 °C and 85 °C, the **86**‐modified devices retained over 85% of their PCE after 200 cycles. These devices demonstrated a record PCE of 25.0% (certified 24.3%) while maintaining excellent stability. Hayase et al. reported the use of a ferrocene derivative, dibenzoylferrocene (**87**), in methylammonium‐free and bromine‐free PSCs [108]. **87** not only passivated defects in the films, but also suppressed ion migration, resulting in a high PCE of 23.53%. Yuan et al. explored a chemical bridging approach using bis‐(diphenylphosphino)ferrocene (**88**) to reconstruct the perovskite interface [109]. Compound **88** forms strong Pb‐P bonds, passivates both grain boundaries and surfaces, and reduces nonradiative recombination, thus enhancing the carrier transport. Devices based on **88** achieved nearly 25% in PCE and retained 95% of their initial efficiency after 1000 h of continuous operation. Qin et al. explored that ferrocene can form a 1D perovskite structure that recovers elemental defects while regenerating ferrocene itself, leading to PSCs that maintain over 10 000 h lifetime in inert condition and over 1000 h of durability under extreme conditions [110]. Then Zhu et al. reported a poly‐ferrocenyl molecule **89** that can modulate the perovskite surface [111]. Their findings indicated that the interaction between perovskite and ferrocenyl group creates a hybrid complex with enhanced surface coordination, which reduces nonradiative recombination and improves charge transport. The distorted geometry of the [PbI_5_]^3–^ pyramid at the perovskite surface, caused by the strong Pb–O interactions, further enhances the device performance. Devices incorporating **89** achieved PCEs of 26.08% and 24.51% for small‐area and large‐area devices, respectively. Fan et al. reported a novel approach employing bis‐ferrocenyl‐carboxylate‐hexadecafluorodecyl (**90)** as an interlayer to improve the interface between the perovskite and the hole transport layer [112]. The interlayer containing **90** blocks ion migration, improves charge extraction, and stabilizes the surface by inhibiting defect‐induced by‐product (I_2_). The resulting devices achieved an outstanding PCE of 23.02% with excellent humidity and long‐term stability.

#### 2.2.2 Copper Phthalocyanine Derivatives

For a long time, the most widely used hole transport materials (HTMs) in PSCs were 2,2′,7,7′‐tetrakis(N,N‐di‐*p*‐methoxyphenylamine)‐9,9′‐spirobifluorene and poly(triarylamine), which played a crucial role in extracting and transferring photo‐generated holes from the perovskite to the cathode, thereby minimizing undesired recombination losses at the interfaces [113, 114]. However, to enhance conductivity and improve efficiency, *p*‐type dopants or additives, such as Li‐TFSI, were commonly incorporated [115]. This created a demand for alternative HTMs for PSCs. Among them, copper(II) phthalocyanine (CuPc) has drawn significant interest due to its high hole mobility, stability, and other advantageous properties. The solubility of phthalocyanine (Pc) structure can be enhanced by incorporating substituents in either the peripheral or non‐peripheral positions. For example, Sun et al. reported a HTM **91** by introducing triisopropylsilylethynyl groups to the periphery of the Pc ring, enhancing its solubility and hydrophobicity properties [116]. Their optimized PSCs based on pristine **91** with no additives or dopants achieved a decent PCE of 14.0% and demonstrated excellent long‐term stability under ambient conditions. This work highlighted the potential of solution‐processed CuPc derivatives as effective and stable HTMs in PSCs. After that, Seo et al. used **92** as HTM to prepare thermally stable PSCs [117]. They found that a strong interfacial and conformal coating exists between HTM **92** and the perovskite layer, leading to PSCs with a high PCE of 18% and remarkable thermal stability, retaining 97% of their initial efficiency after 1000 h of thermal annealing at 85 °C. In another study, Liao et al. developed a method by incorporating F4‐TCNQ into **93** to fabricate planar structure PSCs [118]. They found that the aqueous solution of **93,** doped with 2.5 wt% F4‐TCNQ, had a near neutral pH (7.4), which was favorable for long‐term device stability. Furthermore, the doping improved the film conductivity, hole mobility and HOMO level of **93**, resulting in a PSC PCE of 20.16% in an n–i–p structure. Sun et al. synthesized two CuPc derivatives **94** and **95** and showed that modifying the substituents of the Pc rings by replacing butyl groups with butoxy groups significantly improved molecular ordering, hole mobility, and solar cell performance [119]. Both PCE and stability were enhanced using **95** as the HTM. Xu et al. reported another CuPc‐based HTM (**96**), which demonstrated strong coordination with the perovskite [120]. The annealing process revealed that **96** can infiltrate the bulk of the perovskite film, efficiently passivating both the bulk and interface defects within the perovskite. This can be attributed to the strong interaction between the methylthio group and undercoordinated lead atoms. High‐resolution transmission electron microscopy (HRTEM) further confirmed that **96** was positioned on the surface of the perovskite crystallites and bridged adjacent crystallites. This arrangement contributed to improving the bulk passivation, leading to enhanced performance of the device. The best devices achieved a PCE of 21.51%, which increased to 23.0% upon the incorporation of QAPyBF_4_ additive. Yu et al. introduced a copper naphthalocyanine derivative (**97)** as a HTM in PSCs [121]. The extended π‐conjugation from naphthalocyanine core enhanced both hole‐carrier mobility and the glass transition temperature (*T*_g_) to 252°C. The optimized PSCs utilizing **97** achieved an excellent PCE of 24.03%, with a T_80_ lifetime exceeding 1000 h.

Though great progress has been achieved for CuPc derivatives, several challenges remain. The synthesis of CuPc and its derivatives is complex and costly, and further modifications are needed to enhance the hole mobility. Additionally, the PCEs achieved with CuPc are still lower than those of state‐of‐the‐art HTMs in PSCs. Therefore, continued research effort is necessary to address these limitations and further develop CuPc‐based HTMs. Apart from CuPc, the zinc complex derivatives have also been used as HTM or passivant. Tingare et al. synthesized a zinc‐based coordination complex **98** as HTM for PSC applications [122]. Their results showed that **98** would contribute to the mixed‐halide perovskites with large crystalline grains. **98** exhibited 63.31% higher hole mobility than its nonmetal component HTM. As a result, the BTZ30‐based solar cell exhibited a *V*_oc_ of 1.081 V and a champion PCE close to 20%. Xu et al. introduced a phthalocyanine derivative, **99**, into the perovskite precursor solution to modulate the crystallization process of the perovskite and to passivate the defects [123]. Further research revealed that **99** with electron‐donating properties can engage with the undercoordinated Pb^2+^ ions, diminish the defect density and enhance the overall film quality. A high PCE of 26% was achieved based on **99**, together with a T_90_ over 550 h.

In summary, metal complexes in the PSCs can be used as HTMs and passivants. In the HTMs, CuPc‐based transport layers have been widely reported due to their good charge transport capability and aligned energy levels. From the structure–activity relationships, its robust and planar macrocyclic structure forms a dense hydrophobic film that effectively protects the underlying perovskite layer from environmental degradation. CuPc can function effectively as an HTL without requiring hygroscopic dopants to achieve sufficient conductivity. In the passivants, ferrocene derivatives can function as dual‐functional passivation layer via redox activity and steric hindrance. The functional groups on these molecules can passivate the perovskite, and also modulate the energy alignment with perovskite. Their unique redox activity and structural design go beyond simple defect passivation, offering a comprehensive strategy to enhance both the efficiency and the long‐term stability of perovskite photovoltaics while proactively mitigating lead toxicity issues.

### 2.3 Metal Complexes in Dye‐Sensitized Solar Cells

DSSCs have once represented a hopeful category of solar cells. In these cells, dye molecules function as light absorbers, capturing photons to excite electrons that are then transferred to the semiconductor. Transition‐metal‐based dyes have gained significant attention as effective sensitizers in DSSCs. For instance, ruthenium (Ru)‐complex dyes, such as the well‐known black dye, have demonstrated solar‐energy‐to‐electricity conversion efficiency of 11.18% [24]. Among transition‐metal dyes, Pt(II)‐based bis(aryleneethynylene) complexes have shown potential as photosensitizer for DSSC. Although early reports on Pt complexes as dye sensitizers indicated lower efficiencies, more recent advancements have improved their performance. Tian et al. reported organic sensitizers with a D–π–M–π–A structure, specifically a platinum‐acetylide complex (**100**), and achieved a PCE of 3.28% with a *J*_sc_ of 6.77 mA cm^−2^ and *V*_oc_ of 0.70 V [124]. Wong et al. synthesized three unsymmetric Pt(II)‐bis(aryleneethynylene) derivatives (**101**–**103**), which were comprehensively analyzed for their absorption, electrochemical, impedance, and photovoltaic properties, with computational support from time‐dependent density functional theory (TD‐DFT). These complexes achieved PCEs of 1.56%, 1.42%, and 1.57%, respectively [125]. Later, the same group reported three Pt‐bis(acetylide) dyes (**104**–**106**) based on phenothiazine, each featuring different donor units [126]. They discovered that while modifying the position of the alkoxy chain had a minimal effect on the absorption and energy levels, it significantly influenced the electron lifetime and charge‐transfer resistance. DSSC utilizing **104** achieved the best PCE of 5.78%, along with a notably high *V*_oc_ of 0.74 V.

As early as 1993, Grätzel et al. reported a Ru complex dye **107** which was coated with TiO_2_ films to harvest visible light [127]. The absorption range was broadened to 800 nm, and the conversion of incoming photons to electric current was almost fully efficient. Ultimately, the device utilizing a straightforward molecular light absorber achieves a PCE of approximately 10%. After that, several similar molecules were synthesized consecutively to study the photovoltaic properties [128, 129, 130]. For instance, Arakawa et al. reported a series of mono‐cationic polypyridyl‐ruthenium complexes **108**–**110** [131]. The results showed that the diketonate complexes exhibited excellent performance in photoelectrochemical cells using I^–^:I_3_^–^ electrolyte. Among them, **108** achieved the highest PCE of 6%. Then Grätzel et al. presented two novel heteroleptic polypyridyl ruthenium complexes, designated as **111** and **112**, which featured high molar extinction coefficients [24]. By extending the *π*‐conjugation of spectator ligands, the absorption of the mesoporous titania film was enhanced to improve the charge collection efficiency in a DSSC. With the use of an acetonitrile‐based electrolyte, the sensitizer **111** has demonstrated an impressive efficiency of 11.3%. Then similar structures of **113** [132], **114** [133], **115** [134] and **116** [135] were reported and the PCEs ranged from 8.37% to 11.3%. Olivier et al. reported a Ru(II)‐diacetylide organometallic complex (**117**), which incorporated a push–pull design with an anchoring carboxylic acid group, enabling it to adsorb onto a semiconducting metal oxide thin film, making it suitable as a photosensitizer in hybrid solar cells. Complex **117** achieved a PCE of 7.3% [136].

Besides, several D–π–A structured Zn(II) porphyrins have been examined in DSSCs. Grätzel et al. synthesized a porphyrin dye (**118)** with a typical D–π–A structure that maximized electrolyte compatibility and improved light‐harvesting properties. DSSCs based on **118** exhibited a high *V*_oc_ of 0.91 V, *J*_sc_ of 18.1 mA cm^−2^, FF of 0.78 and a PCE of 13% [137]. Yeh et al. reported two new double‐fence porphyrin dyes (**119** and **120**), which featured long alkoxyl chains attached to *β*‐phenyl groups. These dyes reduced dye aggregation and decreased the charge‐recombination rate, leading to PCEs of 10.69% and 10.62%, respectively [138]. This work demonstrated that the potential of double‐fence structures as an effective strategy for achieving porphyrin sensitizers in high‐performance DSSCs. Recently, Xie et al. reported a porphyrin dye (**121**) featuring a benzo‐12‐crown‐4 (BCE) unit attached to the nitrogen atom of the phenothiazine donor [139]. This modification helped prevent the downward shift of the TiO_2_ conduction band and minimized interfacial charge recombination by binding Li^+^ ions. Moreover, the smaller size of the BCE unit allowed higher dye adsorption, which contributed to an enhanced *J*_sc_ value. **121** afforded an enhanced PCE of 12.1%. This approach demonstrates the effectiveness of introducing crown ether units to improve the performance of porphyrin‐based DSSCs.

Based on the above discussions, from the perspective of structure–activity relationships, metal complexes offer superior tunability of electronic properties, intrinsically long‐lived and charge‐separated excited states, robust stability, and optimal interfacial properties in the DSSCs. Researchers may design and synthesize the metal complex dyes according to the following proposals. First, we can adopt *π*‐conjugation extension, ligand field and MLCT tuning strategies to broaden and intensify light harvesting. Second, we can implement steric and electronic blocking to prevent *π*‐*π* aggregation of dye molecules, hence minimizing self‐quenching. Subsequently, the development of anchoring techniques, such as direct *π*‐conjugation of the chromophoric core and the use of multidentate anchors, can facilitate ultrafast electron injection and enhance electron delocalization within TiO_2_, respectively. The ideal complexes are no longer just a chromophore but a sophisticated interfacial architect, orchestrating each step of the photovoltaic process for maximum efficiency and durability.

In summary, in the photovoltaic fields, metal complexes have established themselves as a versatile and impactful class of materials, serving distinct yet evolving roles across several solar cell technologies. However, the reliance of these materials on scarce precious metals raises serious concerns about material cost, scalability, and environmental impact for large‐scale deployment. Then some high‐performance complexes often require multi‐step synthesis with challenging purification and increasing production cost compared to simpler organic molecules. While tunable, some complexes have weaker absorption in key spectral regions compared to high‐performing organic dyes or perovskite materials themselves. Despite their elegance, metal complexes face substantial hurdles that question their role in terawatt‐scale, cost‐competitive photovoltaic cells. The most effective complexes have historically relied on scarce and expensive precious metals like Pt and Ir. This creates an insurmountable conflict between high performance and low cost/scalability. The material cost and geopolitical supply chain issues alone render these materials impractical for mass‐produced, utility‐scale photovoltaics. High‐performance complexes are rarely simple molecules. The synthesis of some metal complexes often involves multiple steps, inert atmospheres, and challenging purification protocols. This translates to high embodied energy and cost, negating the low‐energy‐production promise of solar cells and complicating large‐scale manufacturing. Metal complexes often win on stability but lose on cost and efficiency ceilings. Developing cheap metal complexes materials and balancing the cost and efficiency will open up new research directions.

## 3 Metal Complexes for Solar–to–Chemical Energy Conversion

**FIGURE 6:**
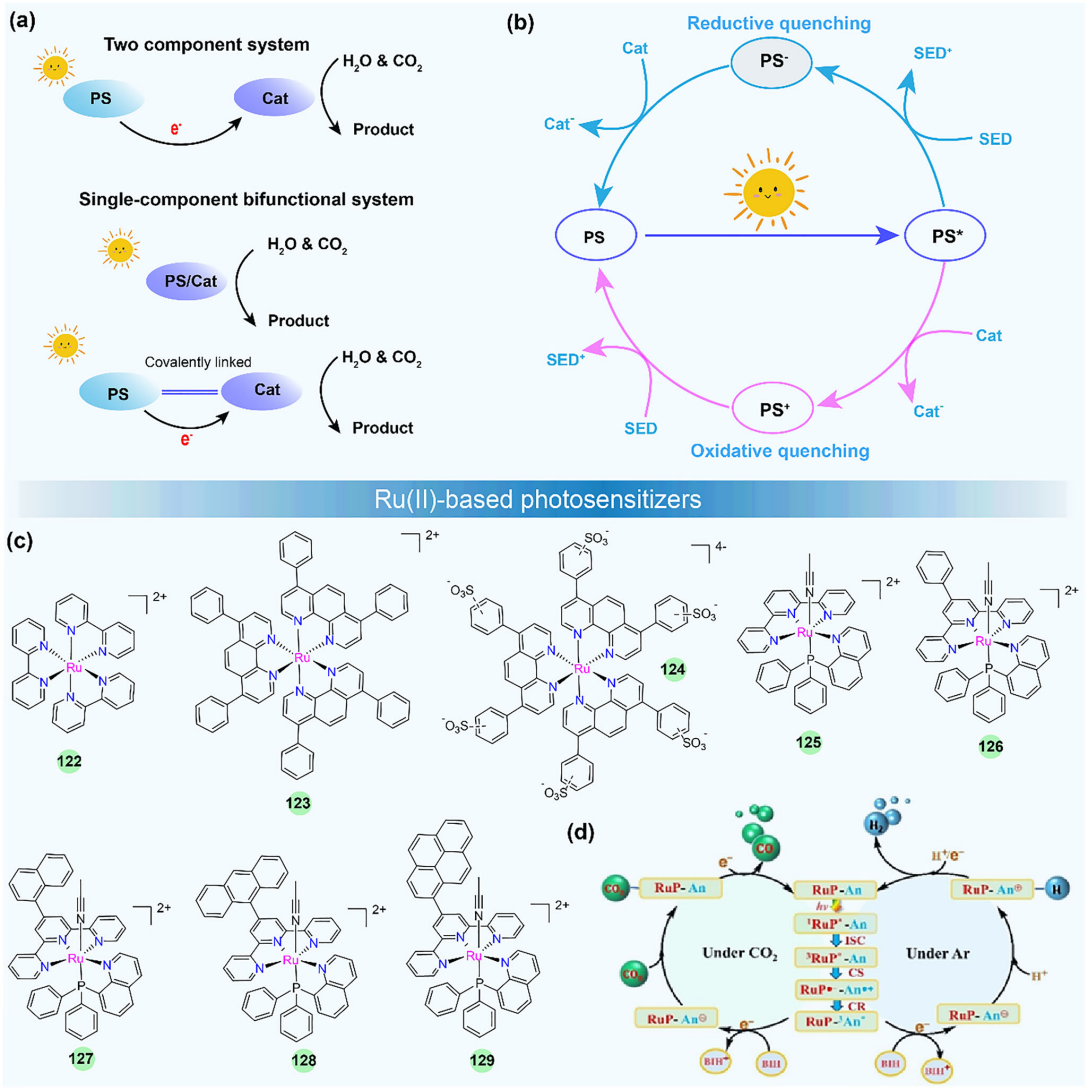
(a) The reported two‐component and single‐component photocatalytic systems. (b) The typical reaction mechanisms of photocatalytic systems consisting of a photosensitizer (PS), a catalyst (Cat), and a sacrificial electron donor (SED). (c) Chemical structures of Ru(II)‐based photosensitizers. (d) The photochemical process of **128** for H_2_ production and CO_2_ photoproduction. (d) Reproduced with permission [155]. Copyright 2024, Wiley‐VCH.

Metal complexes have attracted much attention in photocatalysis in recent years, and they can serve as photosensitizers (PS), catalysts (Cat), electron relays, or structural units in photocatalytic systems [140, 141, 142, 143, 144, 145]. By using solar light, the photocatalytic H_2_ production (PHP), CO_2_ photoreduction, and H_2_O_2_ production are of particular interest. The well‐established photocatalytic systems generally consist of three crucial components: a PS, a Cat, and a sacrificial electron donor (SED). Additionally, the PS and Cat can also be integrated into a single‐component bifunctional molecule capable of functioning as both photosensitive and catalytic centers (Figure 6a). A key step in photocatalysis is light absorption by the PS, which creates a relatively long‐lived triplet excited state through ISC. The excited PS (PS*) can undergo either reductive quenching or oxidative quenching, resulting in the formation of the reduced (PS^−^) or oxidized (PS^+^) forms, respectively. Subsequently, PS^−^ or PS^+^ can return to the ground state by re‐oxidation or re‐reduction. Through these processes, the catalyst can drive the catalytic reaction for water splitting or CO_2_ reduction, achieving the conversion of solar energy into chemical fuels. These processes are schematically represented in Figure 6b. Therefore, the PS is responsible for initiating photo‐induced charge separation within the photocatalytic system and is crucial to the photocatalytic efficiency. Here, we focus specifically on the recent advances of metal complexes used as PSs in photocatalysis by using solar light, including noble‐metal and noble‐metal‐free compounds. However, those efforts with metal complexes by using monochromatic light will not be considered [146, 147, 148, 149, 150]. In addition, in terms of photocatalytic water oxidation, recent studies have overwhelmingly focused on metal complexes as water oxidation catalysts, while the design of new metal complex PSs remains scarce [151, 152, 153, 154]. Therefore, we will not conduct an in‐depth survey of photocatalytic water oxidation, particularly O_2_ evolution, in this review.

For rational design and construction of efficient metallated PSs, several general requirements should be considered: (i) Broad and strong absorption across a significant range of the solar spectrum, particularly in the visible light region, to ensure effective solar energy utilization; (ii) a long excited‐state lifetime (ns∼µs) to facilitate efficient charge transfer; (iii) appropriate redox potentials to enable electron transfer between the SED and the catalyst; (iv) high photochemical stability in solution to ensure a long‐term operation in photocatalysis; and (v) good solubility in water and/or common organic solvents to promote molecular‐level interactions and homogeneous photocatalytic reactions. By adhering to these criteria, the development of metal‐complex‐based PSs can be optimized for enhanced performance in photocatalytic applications.

The production rate, apparent quantum yield (AQY), turnover number (TON), and turnover frequency (TOF) are the primary metrics for the evaluation of photocatalytic systems. In this section, we will present a discussion and summary of the design principles and photochemical characteristics of new metallated PSs, including small molecules, polymers, and assembled supramolecules, and their improved attributes in photocatalytic performance will also be highlighted.

### 3.1 Noble‐Metal‐Based Small‐Molecule Photosensitizers

Noble‐metal‐based small‐molecule PSs, mainly including Ru(II) [156], Ir(III) [157], Pt(II) [158], and Re(I) [159], have garnered wide attention due to their potent driving forces and long‐lived excited states, which make them highly effective in photocatalytic applications.

#### 3.1.1 Ru(II)‐Based Photosensitizers

The Ru(II)‐polypyridine complex represents one of the earliest developed classes of PSs, and their fundamental properties and photosensitization mechanisms have been well investigated [160]. For example, [Ru(bpy)_3_]^2+^ (**122**) (bpy = 2,2′‐bipyridine), as the most well‐studied PS, exhibits intense absorption in the visible light spectrum (λ_max_ = 452 nm in aqueous solution with a high molar extinction coefficient *ε* = 1.5 × 10^4^ M^−1^ cm^−1^) and long excited‐state lifetime (*τ* = 1.1 ms) which determines its reactivity for the electron transfer reaction, as well as remarkably reversible redox potentials, enabling the reduction or oxidation reaction to occur [161]. Such excellent photochemical properties have made **122** commercially available and widely utilized in photocatalysis. There are huge bodies of work and several review articles on Ru(II)‐based PSs in artificial photosynthesis in the past [140, 143, 162]. In contrast, researchers have paid more attention to other metallated PSs for photocatalytic applications. Therefore, this subject will not be surveyed in detail here. In recent years, the development of Ru(II) PSs focuses on the stability improvement and the manipulation of excited states or the exploration of other photocatalytic applications, like CO_2_ photoreduction.

For example, to get a better stable photocatalytic system, the groups of Castellano [163] and Collomb [156] have designed tris‐diphenylphenanthroline Ru(II) PSs **123**–**124** (Figure 6c), which exhibit higher absorption coefficients and much longer excited state lifetimes than regular **122**. The lifetimes of **123** and **124** are 5.86 and 3.8 µs, respectively. **123** exhibits greater stability than **122**, enabling enhanced H_2_ production in a homogeneous photocatalytic system with a catalyst and a SED under visible‐light irradiation. The improved stability and efficiency of Ru(II) PS for H_2_ production in water stem from the large sulfonate‐functionalized phenanthroline ligand, which reduces its susceptibility to dissociation in an aqueous environment upon reduction.

Recently, researchers found that Ru(II)‐polypyridine complexes with phosphine‐substituted ligands have potential applications in energy conversion and catalysis due to their novel and adjustable structures [164, 165]. Masaoka et al. first demonstrated the photocatalytic activity for directly producing CO from CO_2_ using function‐integrated Ru(II) phosphine complex (**125**), with a TON_max_ of 353 over 24 h and 97% selectivity under visible‐light irradiation [165]. In 2024, Wei et al. developed a strategy to significantly extend the triplet excited‐state lifetime by functionalizing **125** with pendant polyaromatic hydrocarbons (PAHs) [155]. Systematic investigations revealed that in anthracene‐decorated **128** (RuP‐An), sub‐picosecond electron transfer from the anthracene moiety to the triplet metal‐to‐ligand charge transfer (^3^MLCT) excited state generates a charge‐separated state, which facilitates the formation of the intra‐ligand triplet state (^3^IL) of anthracene, resulting in an exceptionally long excited‐state with a lifetime of 3.43 ms. The prolonged ^3^IL excited state of **128** involves the spin‐orbital charge‐transfer intersystem crossing (SOCT‐ISC) mechanism, which can significantly enhance the reactivity and robustness of the catalyst. The multi‐functional mononuclear photocatalyst **128** showed impressive performance for CO_2_ photoreduction to CO with 100% selectivity, a quantum yield of 43% at 450 nm, and a TON of 404 without the need for any additional PS or catalyst. The photochemical process for CO_2_ photoproduction is shown in Figure 6d. Under visible‐light irradiation, **128** was excited to its singlet state (^1^RuP*‐An), then to triplet state (^3^RuP*‐An) through ISC. This process generated effective charge separation states (RuP*‐An*^+^) that further underwent recombination to produce long‐lived anthracene triplets (RuP‐^3^An*). Finally, the RuP‐^3^An* was quenched by the SED 1,3‐dimethyl‐2‐phenyl‐2,3‐dihydro‐1H‐benzo[d]imidazole (BIH), yielding the one‐electron reduction species RuP‐An^−^, which subsequently participated in the CO_2_ reduction reaction. The authors further demonstrated the feasibility of complexes **125**–**129** for PHP. Under an Ar atmosphere, the protonated **128** (RuP‐An^+^) could transfer H^+^ to Ru(II)–hydride intermediate during the catalytic cycle for H_2_ generation. This study provides a valuable approach for controlling the excited‐state charge/energy flow dynamics, paving the way for the design of long‐lived ^3^IL multi‐functional mononuclear photocatalysts for converting solar energy into fuel.

#### 3.1.2 Ir(III)‐Based Photosensitizers

Like **122**, Ir(III) complexes display distinctive photophysical properties, such as intense luminescence, millisecond‐scale excited‐state lifetime, and adjustable emission wavelength, which are desirable in photocatalysis [166]. The widely studied cyclometallated Ir(III)‐PSs for sunlight‐driven energy conversion are the type of [Ir(C^N)_2_(N^N)]^+^ (i.e., [Ir(ppy)_2_(bpy)]^+^ **130**), which offers two sites for ligand modification and allows control of the excited‐state energy and dynamics, thereby tuning their photophysical and redox properties.

**FIGURE 7:**
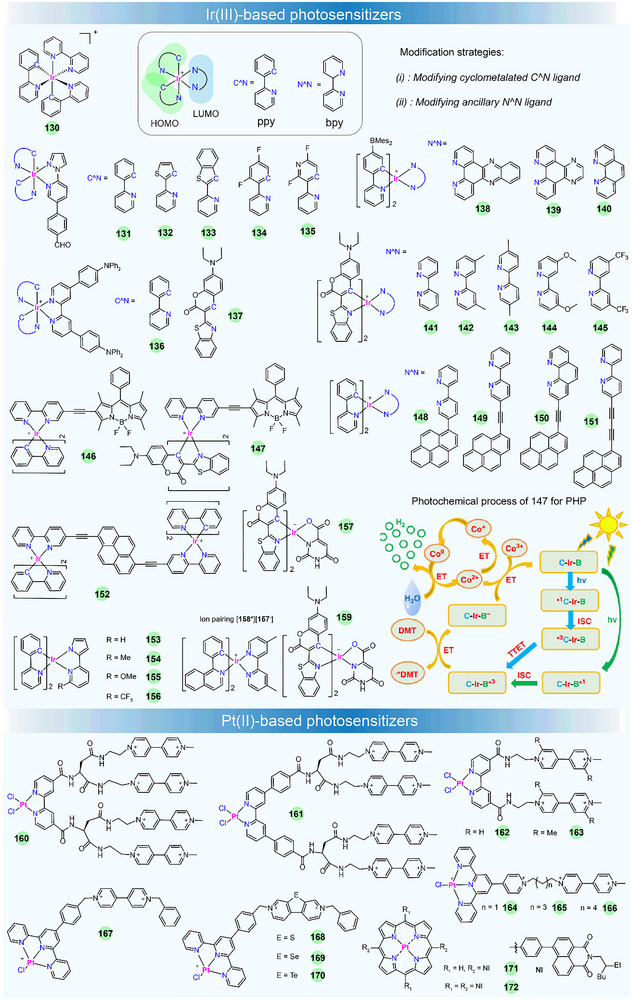
Chemical structures of Ir(III)‐ and Pt(II)‐based photosensitizers. The photochemical process of **147** for PHP is reproduced under the terms of the CC‐BY Creative Commons Attribution 4.0 International license [169]. Copyright 2019, Wang et al., published by Springer Nature.

In such complexes, their luminescence is generally governed by the interaction between the ^3^MLCT state and the strongly spin–orbit‐coupled triplet levels of the cyclometallated ligand. Increasing the electron density on the ancillary N^N ligand or reducing the electron density on the cyclometallated C^N ligand could increase the excited‐state redox reactivity [167]. These distinctive luminescent and redox features have spurred extensive research, spanning from materials design to optoelectronic/photocatalytic applications [168]. In this part, we will focus on the structural design of Ir(III)‐based PSs to control their photosensitizing properties in photocatalysis, such as visible‐light absorption capacity, excited‐state lifetime, electron transfer rate, photocatalytic activity, etc. Some of the recently developed Ir(III)‐based PSs for sunlight‐promoted PHP and CO_2_ reduction are listed in Figure 7 and Table 2.

**TABLE 2:** Representative metal complex‐based photocatalytic systems.

	Photophysical properties					Photocatalytic properties							Refs.
	PS	λ_abs_ (nm) (*ε* [M^−1^ cm^−1^])	λ_em_ (nm)	*τ*_em_	*Φ*_em_ (%)	Catalyst	SED	Solvent	Light source	Product	Rate	TON	
Ir	**137**	361 (3.7 × 10^4^), 456 (1.1 × 10^5^), 480 (1.2 × 10^5^)		41.14 ns	1.3	K_2_PtCl_4_	AA	DMF/H_2_O	300 W Xe lamp λ > 420 nm	H_2_		198363	[170]
	**138**	300, 340, 440	631	0.66 µs		Rh(dtbbpy)_3_(PF_6_)_3_	TEA	CH_3_CN/H_2_O	300 W Xe lamp λ > 420 nm	H_2_		1158	[171]
	**144**	482 (131 000)	586, 629,	21 µs	27.1	Co(dmgH)_2_pyCl	HAscNa	CH_3_CN/AA buffer	300 W Xe lamp, λ > 440 nm	H_2_		97	[172]
	**146**	475, 530	506, 557	89 µs		Co(dmgH)_2_pyCl	DMT	CH_3_CN/H_2_O	175 W Xe lamp λ > 420 nm	H_2_		115840 (12 h)	[169]
	**153**		506	85 ns	0.99	Co(bpy)_3_(PF_6_)_2_	TEOA	DMSO/H_2_O	10 W white LED lamp (λ: 400–750)	H_2_		1768	[173]
	**158**		575, 618	15 µs	50	Co(dmgH)_2_pyCl	HAscNa	CH_3_CN/AA buffer		H_2_		410	[174]
Pt	**169**	310 (18400), 407 (12000)					EDTA	NaCl/acetate buffer	Xe lamp, λ *>*400 nm, 100 mW cm^−2^	H_2_	0.312 mmol g^−1^ h^−1^	58.9	[175]
	**171**	399 (4.6× 10^4^)	634, 703	45.6 µs	79		TEA	THF/H_2_O	white LED lamp (148.5 mW cm^−2^)	H_2_	750 µmol g^−1^ h^−1^		[176]
Cu	**186**	378 (3.1× 10^3^)	564	64.0 ns		Fe_3_(CO)_12_	TEA	THF/H_2_O	Xe lamp (output 1.5 W)	H_2_		305	[177]
	**191**	389 (1628.6)	446		13		TEA	THF/H_2_O	Xe lamp (output 1.5 W)	H_2_		1388	[178]
	**196**	397 (34700)		88 µs	10	K_2_PtCl_4_	TEA	THF/H_2_O	300 W Xe lamp *λ >* 400 nm	H_2_		19000	[179]
	**198**	382 (6.4 × 10^3^)		628 ns	2.1	Fe_3_(CO)_12_	TEA	THF/H_2_O	Xe lamp (output 1.5 W)	H_2_		613	[180]
Zn	**212**	445 (31.44 × 10^4^)	652	2.6 ns	19	Pt	TEOA	H_2_O	white LED lamp (148.5 mW cm^−2^)	H_2_	4.28 mmol g^−1^ h^−1^	67.6 (50 h)	[181]
	**213**	426 (35.88 × 10^4^)	611, 653	3.8 ns	18	CoPyCl	AA	THF/phosphate buffer	white LED lamp (148.5 mW cm^−2^)	H_2_	35.70 mmol g^−1^ h^−1^	5958 (50 h)	[182]
	**216**	455 (11.20 × 10^4^), 493 (15.38 × 10^4^) 547 (21.94 × 10^4^), 730 (16.46 × 10^4^)	778, 824	1.51 ns	8	Pt	AA	THF/H_2_O	white LED lamp (148.5 mW cm^−2^)	H_2_	1.80 mmol g^−1^ h^−1^		[183]
	**219**	242 (11.4 × 10^4^), 320 (7.6 × 10^4^), 422 (23.2 × 10^4^), 552 (2.6 × 10^4^), 589 (0.4 × 10^4^)	595, 647	1.70 ns	12	Pt	TEA	THF/H_2_O	white LED lamp (148.5 mW cm^−2^)	H_2_	56.20 mmol g^−1^ h^−1^		[184]
Bi‐metal	**236**	296, 343, 513	670	69 ns		Pt	TEA	DMSO/H_2_O	300 W Xe lamp λ > 420 nm	H_2_		1088 (80 h)	[185]
	**238**	258 (4.89 × 10^4^), 318 (2.81 × 10^4^), 426 (14.5 × 10^4^), 563 (1.31 × 10^4^), 603 (0.62 × 10^4^)	622, 660	2.5 ns			TEOA	CH_3_CN/H_2_O	white LED lamp (148.5 mW cm^−2^)	H_2_	1.42 mmol g^−1^ h^−1^		[186]
	**239**	260 (1.17 × 10^4^), 445 (1.25 × 10^4^), 571 (1.17 × 10^4^), 615 (0.63 × 10^4^)	535, 626, 670	1.1 µs	< 1	CoPyCl	TEA	CH_3_CN/H_2_O	white LED lamp (148.5 mW cm^−2^)	H_2_	16.12 mmol g^−1^ h^−1^	246 (5 h)	[187]
	**241**	412, 547, 585	587, 634				TEOA	THF/H_2_O	500 W Xe lamp (AM 1.5) (1 Sun)	H_2_	50 mmol g^−1^ h^−1^	640	[188]
	**251**		520	1.94 ns	5.6		BIH	MeCN/CH_3_COOH	300 W Xe lamp, (100 mW cm^−2^)	H_2_	1707 µmol g^−1^ h^−1^	157 (12 h)	[189]
	**PS**	**λ_abs_ [nm] (*ε* [M^−1^ cm^−1^])**	***λ*_em_ [nm]**	***τ*_em_**	***Φ*_em_ [%]**	**catalyst**	**SED**	**solvent**	**Light source**	**Product**	**Selectivity**	**TON**	**Refs**.
Ru	**128**	11035	700	3.43 ms			BIH	DMA	Xe lamp, λ > 400 nm	CO	100%	404	[155]
Ir	**152**	420 (57300)	683	56.7 µs		[Fe(qpy)(H_2_O)_2_]^2+^	BIH	CH_3_CN/H_2_O	Xe lamp (λ>420 nm, 75 mW cm^−2^)	CO	98.4%	28160	[190]
Re	**176**	390					TEOA	DMF	500 W Xe lamp λ > 400 nm	CO	>99%	44	[191]
Cu	**192**					Fe‐salophen	BIH	CH_3_CN/TEOA/LiClO_4_	Xe lamp, λ > 400 nm	CO	96%	1600	[179]
	**199**					Fe catalyst	BIH	NMP/TEOA	1.5 W Hg lamp λ: 400–700 nm	CO	99%	487	[192]
	**204**	390 (3.7 × 10^3^)	565	1.1 µs	2	CoTMPyP	AscHNa	NaHCO_3_ buffer	Xe lamp (λ > 400 nm, 179 mW cm^−2^)	CO/H_2_	77%	2680 (4 h)	[193]
	**207**	388 (5000)	585	10 µs	16	Re(bpy)(CO)_3_Br	BIH	DMA/TEOA	Hg‐lamp λ > 370 nm	CO	99%	580 (30 min)	[194]
	**208**	566 (3.85 × 10^4^)	693	0.98 ns	0.82	Fe‐porphyrin	BIH	DMF	white LED lamp λ > 400 nm	CO/H_2_	95%	16109 (23 h)	[195]
Zn	**225**	488 (127461)	517	164.5 µs	54	[Fe(qpy)(OH_2_)_2_]^2+^	BIH	CH_3_CN/H_2_O	300 W Xe lamp λ > 400 nm (80 mW cm^−2^)	CO	100%	20000	[196]
Fe	**232**						BIH	CH_3_CN/TFE	300 W Xe lamp λ ≥ 400 nm	CO/HCOO^−^	92.5%	46 (4 h)	[197]
Bi‐metal	**245**	459, 365	656	649 ns	9		BNAH	DMF/TEOA	500 W Hg lamp λ > 500 nm	CO		204 (20 h)	[198]
	**247**	343 (26 × 10^3^), 444 (8.0 × 10^3^)	595, 635	3.0 µs	35		BIH	DMA/TEOA	500 W Hg lamp λ > 500 nm	CO	99.9%	1700	[199]
	**248**	305 (50× 10^3^) 418 (418.9 × 10^3^) 544 (22.4 × 10^3^)	588, 638	2.23 ns	2.2		TEOA/BIH	DMF	200 W Xe lamp, λ > 450 nm	CO		195 (24 h)	[200]
	**249**	327 (26.4 × 10^3^) 365 (15.5 × 10^3^)	597	33 ns	0.82		BIH	DMF	200 W Hg lamp λ > 400 nm	CO		300	[201]
	*trans*‐**250**	258 (4.70 × 10^3^), 296 (4.27 × 10^3^), 376 (3.95 × 10^3^)	598, 698, 772	15 µs			BIH	DMF/TEA	AM 1.5 G, 100 mW cm^−2^	CO		40000	[202]

By modifying the C^N ligand with different electron‐withdrawing/donating substituents, Wang and coworkers designed a family of cationic Ir(III) complexes (**131–135**) and employed them as PSs in PHP systems with K_2_PtCl_4_ as catalyst and triethylamine (TEA) as SED [203]. The –CHO group aimed to enhance the interactions between the PSs and catalysts, significantly reducing the degradation of PSs [204]. Among them, **133** showed the highest *ε* of 7.8 × 10^4^ M^−1^ cm^−1^. Upon photoexcitation, these Ir(III) complexes exhibited distinct luminescence spanning the visible spectrum from blue‐green (474 nm) to red (645 nm), arising from the ^3^MLCT and triplet ligand‐to‐ligand charge transfer (^3^LLCT) transitions. The excited‐state lifetimes varied from **131**–**133** (104, 265, and 459 ns) to **134** and **135** (1408 and 2051 ns). The HOMO energy levels of such complexes decreased from –5.58 to –6.76 eV with increasing the strength of electron‐withdrawing substituents. The excited‐state reduction potentials (E*_red_ vs NHE) of **131**–**135** were in the order of **135** (1.40 V) > **134** (1.30 V) > **131** (1.15 V) > *E*(TEA^+^/TEA) (0.93 V) > **132** (0.89 V) > **133** (0.74 V), indicating that **131, 134** and **135** possessed sufficient thermodynamic driving force for photoinduced electron transfer from TEA. The PHP followed the order of **135** (312 µmol) > **134** (241 µmol) > **131** (125 µmol) > **132** (60 µmol) > **133** (28 µmol).

Recently, Wang et al. developed two novel Ir(III) PSs **136** and **137** by introducing strong visible‐light‐absorbing coumarin to the C^N ligands and triphenylamine (TPA) to the ancillary N^N ligand, respectively [170]. Compared to **130,** both **136** and **137** showed increased absorption intensity and range in the visible region, but decreased excited‐state lifetime and PLQY. This reduction was attributed to the enhanced nonradiative decay promoted by the intramolecular motions of benzene rings in TPA and vibrations of ester and diethylene groups in coumarin [205]. The HOMO and LUMO energy levels of **137** were lower than those of **136**, likely due to the coumarin that reduced the charge density of Ir center. The PHP experiments were measured in the presence of PS, catalyst K_2_PtCl_4_, and SED ascorbic acid (AA) in DMF/H_2_O (v/v = 3/1). The TONs were 542 for **130**, 167 for **136,** and 2418 for **137**, respectively. The photocatalytic durations were 98 h for **130**, 97 h for **136,** and 247 h for **137**, respectively. Under optimized conditions, **137** achieved superior photocatalytic activity with a TON of 18496 within 10 h, and further reached up to 198363 over 214 h, setting a benchmark of transition metal complexes for PHP by achieving dual improvements in both PS activity and durability.

Yang et al. prepared three dimesitylboron (BMes_2_)‐containing Ir(III) PSs **138**–**140** [171]. The functionalization of the C^N ligand with BMes_2_ endowed these complexes with strong absorptivity and high PLQY because of the empty *p*‐orbital on the electron‐deficient boron center, which facilitated MLCT transitions [206]. Increasing the conjugation of the chromophore's aromatic system promotes enhanced electron delocalization upon photoexcitation, thereby stabilizing the photoexcited state. The E*_red_ of these complexes ranges from 1.31 to 1.72 V, implying that **138**–**140** may provide sufficient thermodynamic driving force for the electron transfer. After 20 h of light irradiation, the TON values were 690, 188, and 303 for **138**, **139,** and **140**, respectively. Under optimized conditions, the maximum TON of **138** was approximately 1158.

By a simple chemical modification of the bpy ligand with Me, OMe, and CF_3_ groups, Takizawa et al. synthesized a series of Ir complexes **141**–**145**, and investigated the substituent effects on PHP activity [172]. **141**–**145** displayed strong absorption in the visible region (*ε* = 126 000–132 000 M^−1^ cm^−1^) and incredibly long phosphorescence lifetimes (21–23 µs) dominated by the coumarin ligands. In the presence of [Co(dmgH)_2_pyCl] as catalyst and sodium ascorbate (HAscNa) as SED, complexes **142**–**144**, bearing electron‐donating substituents, exhibited the same initial rates of H_2_ generation. The unsubstituted complex **141** was markedly less active, whereas the CF_3_‐substituted **145** yielded negligible H_2_ after 40 min of irradiation. The TON_cat_ was 97 in 3 h for complex **144**. Unfortunately, the poor water solubility of these complexes forced the use of large volumes of CH_3_CN, precluding the development of green and scalable systems. To address this challenge and expand the utility of the Ir(III) PSs in pure aqueous solutions, the use of vesicles as scaffolds was adopted. By incorporating **142** into the 1,2‐dipalmitoyl‐*sn‐glycero*‐3‐phosphocholine (DPPC) vesicles, the system achieved a better performance with a TON_cat_ of 150 [207].

As demonstrated above, implanting conjugated groups or strong absorbing chromophores (such as Bodipy and coumarin) into Ir complexes is an effective strategy for fine‐tuning the electronic structures of PSs to improve solar energy utilization and achieve efficient water splitting. In this regard, Lu et al. developed a novel Ir PS **147** comprised of cyclometallated coumarin ligands and ancillary bpy ligand modified with Bodipy moiety [169]. This complex displayed strong visible‐light absorption spanning 400–575 nm, covering approximately 50% of the visible spectrum. For comparison, three additional complexes **130**, **141** and **146** were also synthesized. The PHP activity was evaluated in CH_3_CN/H_2_O with [Co(dmgH)_2_(py)Cl] as catalyst and dimethyl paratoluidine (DMT) as SED. The TONs in 12 h were in the order of **147** (115840) > **141** (22560) > **146** (8270) > **130** (361). Notably, the photocatalytic activity of **147** was almost the sum of **141** and **146**, indicating efficient intramolecular energy transfer from coumarin to Bodipy in **147**. Both **146** and **147** showed long‐lived triplet states with lifetimes of 101 and 89 µs, respectively, supplying enough time for efficient electron transfer. In contrast, the triplet lifetimes of **130** and **141** were merely 307 and 85 ns, respectively, which was less favorable for electron transfer. Additionally, four complexes showed long‐lived reduced states, with lifetimes of 63.0, 25.0, 113.0, and 88.7 µs for **130**, **141**, **146** and **147**, respectively. The second‐order rate constants (*k*_r_) for electron transfer from the reduced PSs to the catalyst were 2.0 × 10^10^ for **130**, 1.7 × 10^11^ for **141**, 2.8 × 10^10^ for **146**, and 8.1 × 10^10^ M^−1^ s^−1^ for **147**, respectively. These values are close to the diffusion‐controlled limits. The reduction quenching pathway dominated the catalytic process for **147**‐containing system. Consequently, the broadband and strong visible‐light‐absorbing ability, long‐lived triplet state, and long‐lived reduced state rendered **147** with dramatically improved PHP performance. DFT calculations also verified the efficient energy transfer from coumarin to Bodipy and the long‐lived Bodipy‐localized ^3^IL state that effectively triggered the electron transfer for redox reactions. The photocatalytic cycle process of **147** (C‐Ir‐B) for hydrogen evolution is shown in Figure 7. Under excitation, **147** with multichromophores displayed dual excitation channels. By excitation of the coumarin unit, the coumarin in **147** absorbed light and converted excitation energy into Bodipy‐localized triplet state ^3^[C‐Ir‐B*] by intramolecular triplet‐triplet energy transfer (TTET) via the pathway of [C‐Ir‐B] → ^1^[*C‐Ir‐B*] → ^3^[*C‐Ir‐B] → ^3^[C‐Ir‐B*]. By exciting the Bodipy part, **147** underwent a photochemical process of [C‐Ir‐B] → ^1^[C‐Ir‐B*] → ^3^[C‐Ir‐B*] by ISC. The resulting ^3^[C‐Ir‐B*] accepted an electron from DMT to form reduced [C‐Ir‐B]^−^, which efficiently delivered electrons to the catalyst to produce Co(I) and drove hydrogen evolution reaction. This work provides a foundation for developing multicomponent arrays for efficient solar light conversion.

The modification of conjugated groups enables the transition from ^3^MLCT state to ^3^IL state, simultaneously boosting visible absorption, prolonged excited‐state lifetimes, and fine‐tuning the excited‐state redox potentials [208]. Recently, Wang et al. developed a family of Ir(III) PSs (**148**–**152**) to explore the structure–activity correlation of PSs [190]. Their photocatalytic activities for CO_2_ reduction were examined under Xe‐light irradiation with Fe(qpy)(H_2_O)_2_]^2+^ (qpy = 2,2′:6′,2″:6″,2″′‐quaterpyridine) as catalyst and 1,3‐dimethyl‐2‐phenyl‐2,3‐dihydro‐1H‐benzo[d]imidazole (BIH) as SED. Among them, **152** achieved the highest TONC_‐1_ up to 9150 after 30 h illumination, representing a 54‐fold enhancement compared to the typical Ir PSs **130**, and the optimized TON_C‐1_ can reach up to 28160. **148**–**151** also exhibited considerable CO_2_ photoreduction abilities, with TONs in the order of **152** (9150) > **149** (6010) > **151** (4530) > **150** (3350) > **148** (2350) > **130** (170).

All these PSs exhibited increased visible‐light absorption compared to **130** owing to the expanded conjugated system and increased heavy atom content [209]. The *ɛ* value was in the order of **152** (57 300 M^−1^ cm^−1^) > **151** (41500 M^−1^ cm^−1^) > **149** (33000 M^−1^ cm^−1^) > **150** (30 200 M^−1^ cm^−1^) > **148** (9600 M^−1^ cm^−1^) > **130** (3720 M^−1^ cm^−1^). Under an Ar atmosphere, **130** exhibited characteristic ^3^MLCT emission centered at 585 nm. In contrast, the other PSs displayed redshifted emissions relative to **130**. Notably, **148**–**152** showed distinct fine‐structured emissions with prominent shoulder peaks from 710 to 780 nm, demonstrating the presence of ^3^IL emissive state. Nanosecond transient absorption spectra indicated that these PSs had long excited‐state lifetimes, in the order of **150** (92.4 µs) > **151** (60.5 µs) > **149** (60.1 µs) > **152** (56.7 µs) > **148** (53.3 µs) > **130** (0.3 µs), which contributed to intermolecular electron transfer. Combined with PL quenching experiments, the reductive mechanism primarily governed the electron transfer process in these PS‐containing systems. The *k*_r_ followed the order: **149** (4.6 × 10^9^ M^−1^ s^−1^) > **152** (3.7 × 10^9^ M^−1^ s^−1^) > **150** (3.0 × 10^9^ M^−1^ s^−1^) > **148** (1.8 × 10^9^ M^−1^ s^−1^) > **151** (5.8 × 10^8^ M^−1^ s^−1^). Although the *k*_r_ of **149** was ∼10 times higher than that of **151**, the CO yield of **149** was only slightly higher than that of **151**, as the strong visible‐light absorption of **151** compensated for other factors. These results indicate that once PSs possess sufficient excited‐state lifetimes, comparable thermodynamic driving forces, and electron transfer rates, their visible‐light absorption capacity becomes the dominant factor for enhancing photochemical CO_2_‐to‐CO conversion.

In 2021, Zhou et al. designed a family of neutral Ir complexes **153**–**156** featuring a novel anionic N^N ligand (pyridylpyrrole) for improving the efficiency and stability of the PHP systems [173]. The catalytic activities of **153**–**156** were evaluated in DMSO‐water system containing [Co(bpy)_3_](PF_6_)_2_], triethanolamine (TEOA) and LiCl salt. The PHP capability followed the order of **153** (TON of 1768) > **155** (TON of 722) > **130** (TON of 651) > **154** (TON of 592) > **156** (TON of 120). Complex **153** demonstrated better photostability than **154**–**156** and maintained PHP activity after long‐term irradiation for 144 h. This can be attributed to the significant steric hindrance imposed by the substituent at the *α*‐position of the pyridine ring in the N^N ligand, which substantially weakened the Ir–N bond strength, allowing for the solvent to coordinate to the metal center. Complexes **153**–**156** displayed identical and unstructured emission at ∼510 nm with lifetimes in the range of 85–365 ns. Theoretical calculations revealed that complexes **153**–**156** with LLCT and LC characters were distinguished from the typical cationic **130** with MLCT transition, owing to the *π* donor character of pyridylpyrrole. The excited‐state reductive potentials *E*(PS^+^/PS*) of complexes **153**–**156** ranged from −1.56 to −1.70 V, significantly lower than that of catalyst [Co(bpy)_3_](PF_6_)_2_ (−0.95 V vs NHE), providing sufficient driving force for electron transfer from the PS* to the catalyst. However, the excited‐state oxidative potentials *E*(PS*/PS^−^) of **153**–**156** were in a range of 0.57–0.74 V, lower than that of TEOA (0.84 V vs NHE). Consequently, the oxidative quenching pathway dominated the catalytic process for these complexes. The quenching constant for **153** was 2.04 × 10^11^ M^−1^ s^−1^, significantly higher than that of the other complexes (5.14 × 10^6^ to 5.39 × 10^10^ M^−1^ s^−1^), indicating that complex **153** was more efficient in transferring electrons to the catalyst than others.

Anionic Ir(III) complexes are rarely reported as PSs in PHP. To explore the possibility, Takizawa et al. synthesized an anionic complex **157** featuring coumarin as the C^N ligand and a dianionic orotate ligand [210]. In CH_3_CN/H_2_O, **157** exhibited intense visible‐light absorption (*ε* = 78 800 M^−1^ cm^−1^), a high PLQY (50%), and a long‐lived excited state (15 µs) due to the ligand‐centered (coumarin‐centered) electronic transition. Subsequently, the PHP experiment in CH_3_CN/acetate/acetic acid buffer solution with **157** as PS, HAscNa as the SED, and [Co(dmgH)_2_pyCl] as catalyst was explored. **157** achieved TONs of 410 relative to the PS, indicating the feasibility of using an anionic Ir PS for PHP, although the TONs were lower than the previously reported cationic PSs [169, 172]. The results have motivated researchers to explore new ion‐paired photocatalysts or iridium soft salts based on **157**.

In 2023, the author further designed an ion pair (**159**) comprising cationic and anionic Ir PSs for photocatalytic CO_2_ reduction at vesicle membranes. The cationic component provided good stability, while the anionic component ensured effective visible‐light absorption [174]. This durable cationic complex [**158** ^+^] showed much lower visible light absorptivity (*ε* ≈ 8000 M^−1^ cm^−1^ at 444 nm) compared to the anionic *n*‐Bu_4_N^+^[**157** ^−^] featuring the coumarin‐centered electronic transition. Therefore, the ion pair **159** exhibited a characteristic absorption band within 400–500 nm dominated by [**157** ^−^] with a minor contribution from [**158** ^+^]. Its absorption spectrum nearly matched the sum of the molar absorptivities of the reference complexes [**158** ^+^]Cl^−^ and *n*‐Bu_4_N^+^[**157** ^−^], indicating no ground‐state electronic interaction between [**158** ^+^] and [**157** ^−^]. Moreover, **159** exhibited [**157** ^−^]‐like phosphorescence, which enabled efficient triplet energy transfer from the visible‐light‐harvesting anionic [**157** ^−^] to the more stable redox‐active cationic [**158** ^+^]. Since ion pairs [211] typically undergo solvation‐induced dissociation in the polar solvents (e.g. DMF), the lipid vesicle membranes [212] as scaffolds for the proposed system were constructed, which could also improve the usability of water‐insoluble ion pairs in aqueous environments. The photosensitizing performance of ion pair at the vesicular membrane surface was evaluated using Re(dtb)(CO)_3_Cl (dtb = 4,4′‐ditridecyl‐2,2′‐bipyridyl) and ascorbate (Asc−) as the catalyst and SED, respectively. Notably, **159** produced the highest amount of CO after 3 h of photoirradiation with the TON_Re_ of 50, accompanied by a high selectivity of 94. This result suggests that the ion pair Ir PS has a significant potential for developing new photocatalytic systems.

#### 3.1.3 Pt(II)‐Based Photosensitizers

Pt(II) complexes are particularly fascinating as catalysts for promoting PHP due to their relatively low applied driving force [213]. Additionally, the long‐lived emissive ^3^MLCT excited state of the Pt(II) complexes makes them efficient PSs, capable of driving the subsequent proton and electron transfer processes. In this regard, besides the catalyst itself, researchers are more likely to design PS‐catalyst dyads where the photo‐induced electron transfer process can be cooperatively coupled with the catalytic process within a single‐molecular architecture. For a long time, Pt(II) complexes represent the sole family of molecular catalysts capable of promoting the methyl viologen (MV^+•^)‐driven PHP (i.e., 2MV^+•^ + 2H^+^ → 2MV^2+^ + H_2_). For example, Sakai et al. prepared two [Pt(bpy)Cl_2_] derivatives (**160** and **161**, in Figure 7) with 4,4′‐diphenyl‐2,2′‐bipyridine (dpbpy) and bpy tethering four pendant viologen acceptors at the end of the ligand [214]. These complexes showed improved photocatalytic activity, corresponding to such series without multi‐viologen units [215]. The enhancement was attributed to the multi‐viologen moieties serving as a light‐harvesting and H_2_‐evolving center. Complex **161** (TON = 35, 12 h) demonstrated twice higher photocatalytic efficiency than **160** (TON = 18, 12 h), consistent with the higher absorptivity of **161** at the MLCT band because of the increased π‐conjugation in dpbpy relative to bpy. Multiple MV^2+^ units enabled rapid regeneration of the one‐electron‐reduced MV^+•^ species via intramolecular electron transfer (ET) from the PtCl_2_(bpy^−•^) unit to one of the MV^2+^ units, driving the PHP process. The PHP activity of this single‐component Pt(II)‐based photocatalyst, tethered to redox‐tuned viologen acceptors, could be controlled by finely tuning the viologen units with substituents. For instance, photocatalyst **163** (TON = 4.2, 5 h) exhibited much higher HER than **162** (TON = 1.8, 5 h) [216], attributed to the multiple HER pathways driven by doubly and triply reduced species generated through consecutive photo‐driven steps.

To improve the PHP activity, three chloro(4′‐(*N*‐methylpyridinium)‐2,2′:6′,2″‐terpyridine)platinum(II) (PtL^2+^) derivatives **164**–**166** tethered to a single alkyl viologen unit were synthesized and thoroughly investigated [217]. These complexes exhibited similar ligand‐centered and MLCT transitions with a contribution of the halide‐to‐ligand charge transfer transition in their absorption spectra. The TONs for **164**–**166** (TON = 21.5–25.2) were significantly higher than that of PtTpy^2+^ (TON = 4.1) [218], attributed to the extra electron reservoir (MV^2+^ unit). However, the PHP activity of **164**–**166** did not exhibit dependence on the length of the alkyl linker between PtL^2+^ and MV^2+^ units.

Compared with conventional viologens, chalcogenoviologens (EV^2+^) exhibit more redox centers and narrower bandgaps due to the effective σ*–π* hyperconjugation. Consequently, EV^2+^ can act as PS and electron transfer media, promoting the rapid electron transfer in photocatalytic system. He et al. designed a series of new Pt(II)‐based photocatalysts **167**–**170**, in which EV^2+^ was covalently linked with a terpyridyl Pt complex [175]. Compared to **165**, the molar absorptivities of **168**–**170** at 370–425 nm were significantly enhanced while preserving their electron–accepting properties. The electron‐transfer constants (*k*_ET_) followed the order of **169** (1.16 × 10^−3^ cm s^−1^) > **168** (1.14 × 10^−3^ cm s^−1^) > **170** (9.56 × 10^−4^ cm s^−1^) > **167** (9.23 × 10^−4^ cm s^−1^), indicating the faster electron transfer property of **169** than others. The PHP tests were conducted in NaCl and acetate buffer solution containing **168**–**170** as catalyst and ethylenediaminetetraacetic acid disodium salt (EDTANa) as SED. Within 72 h, **169** showed the highest H_2_ production (HER of 0.312 mmol g^−1^ h^−1^, AQY of 5.09 × 10^−4^, and TON of 58.9), while **168** and **170** showed a TON of 58.9 and 15.8, respectively. Under visible light irradiation, the efficient intramolecular ET process between PtL^+^ and EV^2+^ induced the generation of radical cation state of EV^+•^, which drove the H_2_ production.

Recently, we have prepared two new Pt(II) complexes **171** and **172** [176]. In the structure, an efficient Förster resonance energy transfer (FRET) occurred between the naphthalimide (NI) energy donor and the porphyrin energy acceptor, which not only enhanced the light‐harvesting ability of porphyrins in the visible light region but also facilitated photoinduced charge separation. Especially, **171** containing two NI units showed stronger phosphorescence emission (QY = 79%) and longer triplet excited‐state lifetime (45.6 µs) than **172** with four NI units. Additionally, the higher excited‐state reduction potential of **171** (1.77 eV) than **172** (1.59 eV) suggested a higher thermodynamic driving force of **171**. As a result, **171** exhibited a higher HER of 750 µmol g^−1^ h^−1^ than **172,** with a HER of 300 µmol g^−1^ h^−1^.

#### 3.1.4 Re(I)‐Based Photosensitizers

**FIGURE 8:**
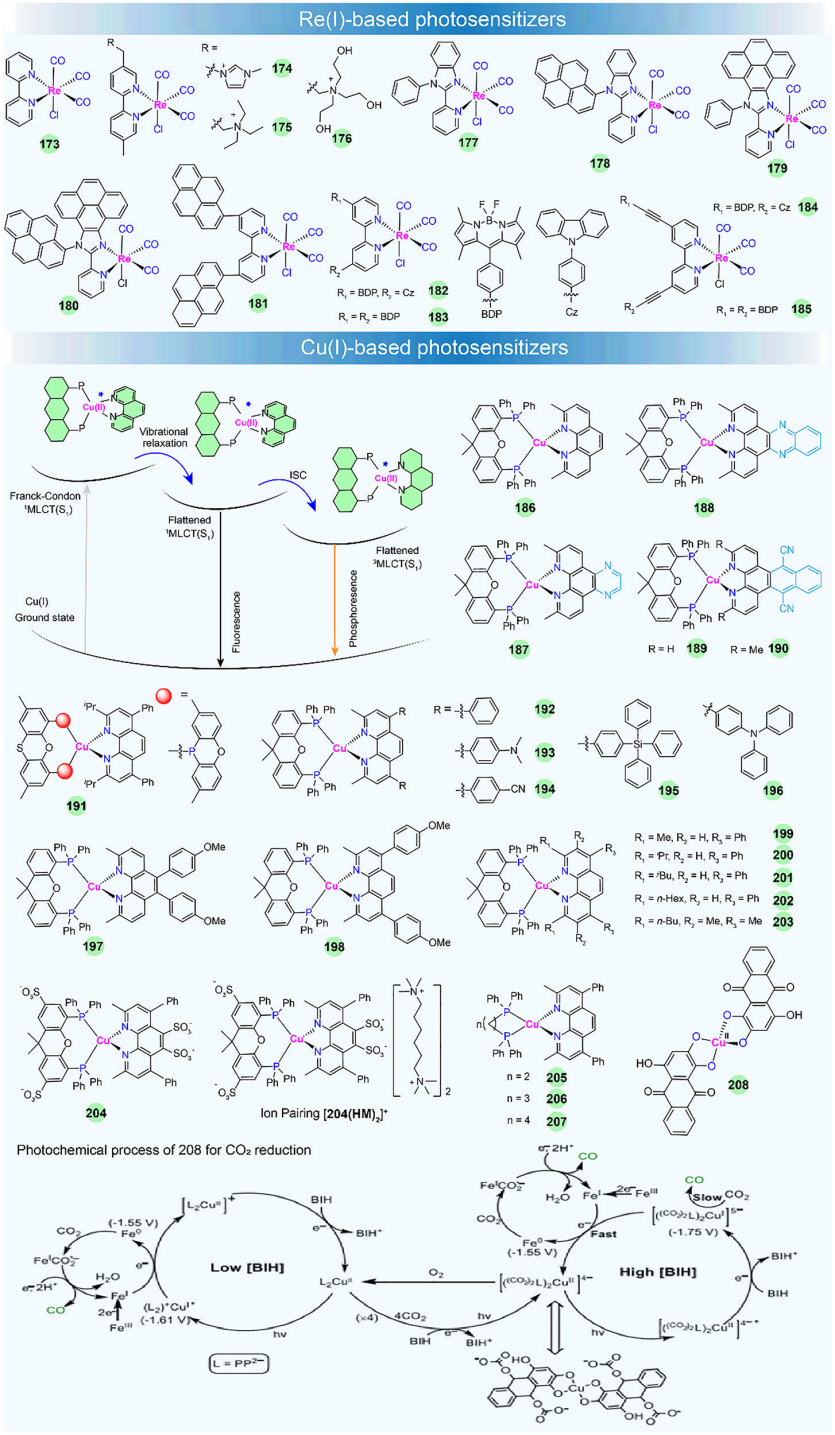
Chemical structures of Re(I)‐ and Cu(I)‐based photosensitizers. The photochemical process of **208** for CO_2_ reduction is reproduced under the terms of the CC‐BY Creative Commons Attribution 4.0 International license [195]. Copyright 2021, Yuan et al., published by Springer Nature.

Re(I) PSs are commonly found in the family of [Re(bpy)(CO)_3_Cl] (**173**) complexes and widely used for solar‐energy‐driven CO_2_ reduction due to their excellent selectivity toward CO. Moreover, the Re(I) complexes can function as both PSs and catalysts in this reaction [219, 220]. This section focuses on the structural modifications of Re(I)‐based PSs in selective CO_2_ photoreduction. He et al. developed a series of highly efficient Re(I) complexes for photocatalytic CO_2_ reduction by fine‐tuning their structures (Figure 8). Consequently, the visible light absorption, triplet excited state lifetime, and electron transfer efficiency were enhanced, leading to improved photocatalytic performance.

For example, three Re catalysts (**174**–**176**) modified with an ionic secondary coordination sphere were designed for photocatalytic CO_2_ reduction [191]. By tuning the structures of ionic groups, **176** exhibited the best activity (TON_CO_ = 44), outperforming **173** (TON_CO_ = 24), **174** (TON_CO_ = 8) and **175** (TON_CO_ = 26). Compared to the pendant imidazolium group, the triethylamine group showing relatively weak electron‐deficient properties, enhanced the visible‐light absorption ability of Re catalyst. The hydroxy group, acting as both chromophore and local proton source, enhanced the reaction. DFT calculations and experimental outcomes suggested that the tris(2‐hydroxyethyl)‐amino group on **176** enhanced visible‐light absorption, stabilized reaction intermediates, and suppressed the formation of Re–Re dimer, thereby improving the catalytic performance.

Based on the imidazole‐pyridine skeleton, He et al. designed a new set of Re(I) complexes (**177**–**180**), focusing on the influence of substitutions on their photocatalytic properties [221]. The visible light‐driven CO_2_ reduction experiments were performed in DMF solutions containing TEOA as proton source and BIH as SED. The photoreduction ability of **177**–**180** followed the order of **180** (TON_CO_ = 124) > **178** (TON_CO_ = 81) > **179** (TON_CO_ = 57) > **177** (TON_CO_ = 22). The superior catalytic performance (AQY_CO_ = 24%, > 99.9% selectivity) of **180** was attributed to its strong visible light absorption, long‐lived triplet lifetime (164.2 µs) and large reductive quenching constant (224000 M^−1^).

Through the introduction of bifunctional pyrene groups into the ligand, He et al. found that **181** exhibited excellent CO_2_ photoreduction performance (TON_CO_ up to 350 ± 36, AQY_CO_ up to 46.6 ± 3%), which was 40‐fold higher than that for **173** (TON_CO_ = 22 ± 2)). This was the first report of a single‐molecule photocatalyst for natural light‐powered CO_2_ reduction. By combining the experimental and calculation results, the appended pyrene groups have been proven to enhance the visible‐light absorption of the catalyst and facilitate electron transfer through intermolecular π–π interactions, leading to efficient photocatalytic performance in solar‐energy‐driven CO_2_ reduction [222].

By appending *N*‐phenylcarbazole and BF_2_‐chelated dipyrromethene (BDP) groups on Re(bpy) skeletons, He et al. designed four novel Re‐based photocatalysts **182**–**185** [223]. Among them, the complexes with two BDP units exhibited a longer‐lived excited state (182.4 µs) and twice the molar absorptivity (*ɛ* = 157 000 M^−1^ cm^−1^) compared to that containing one BDP. The photocatalytic reactivity followed the order of **183** > **182** > **185** > **184** > **173**. Under optimized conditions, **183** achieved superior photocatalytic reactivity and stability with a TON_CO_ of 1323 and AQY_CO_ of 55% after 30 h of light irradiation. Consequently, the boron substituents not only served as PSs but also tuned the redox potential, ET efficiency, and excited state lifetime of BDP‐modified Re(bpy) systems, offering a promising strategy for solar‐to‐fuel conversion schemes.

### 3.2 Noble‐Metal‐Free Small‐Molecule Photosensitizers

Using earth‐abundant metals to replace noble metals in photocatalysis holds significant research importance. Notably, the non‐noble transition metal PSs are gaining increasing attention for various photocatalytic applications and energy utilization, due to their excellent photophysical properties and low cost [224, 225, 226, 227, 228, 229]. This section systematically reviews the recent advances in Cu(I)‐, Zn(II)‐, and Fe‐based PSs, highlighting their design strategies and catalytic applications.

#### 3.2.1 Cu(I)‐Based Photosensitizers

Luminescent Cu(I) complexes have emerged as promising noble‐metal‐free PSs. In contrast to other 3*d* transition noble‐metal complexes, their filled *d* ^10^ electronic configuration inhibits the Franck‐Condon population of metal‐centered states while maintaining prominent MLCT bands, endowing them with excellent visible‐light harvesting capabilities [141, 144]. Under light irradiation, the ground‐state Cu(I) complex is excited into a ^1^MLCT state, which undergoes vibrational relaxation to ^1^MLCT flattened geometry and then to triplet ^3^MLCT flattened state via spin‐forbidden intersystem crossing (ISC). Due to the flattening distortion, the ^3^MLCT state of Cu(I) complex could or could not bind the solvent molecule, depending on the coordination ability of the solvent. Finally, the flattened excited ^1^MLCT or ^3^MLCT state returns to the ground state through radiative relaxation, producing fluorescence or phosphorescence emission (Figure 8) [230]. Homoleptic [Cu(N^N)_2_]^+^ and heteroleptic [Cu(P^P)(N^N)]^+^ complexes, where N^N and P^P represent chelating diamine and diphosphine ligands respectively, have been reviewed for their use as PSs in photocatalytic applications [231, 232]. Compared to their homoleptic counterparts, heteroleptic Cu‐based PSs have garnered increasing attention due to their enhanced emission, longer‐lived and highly‐oxidized excited states, as well as their facile in‐situ generation within the photocatalytic systems using Cu(I) salts. As a result, they often exhibit superior photocatalytic performance. In this section, we will primarily review the advances and improvements in the structural design of heteroleptic Cu‐based PSs by modifying the N^N or P^P ligands, aiming to optimize their promising photophysical properties, e.g., visible‐light absorption, luminescence, excited‐state lifetime, and reducing ability, and to highlight their most recent applications in PHP and CO_2_ reduction.

For PHP applications, Karnahl et al. investigated the impact of an extended π‐system in the backbone of N^N ligand of **186**–**188** PSs on the PHP activity in the presence of TEA and [Fe_3_(CO)_12_] [177]. The π‐extension had little impact on their structural properties, but decreased the emission lifetimes (64–14.5 ns) and catalytic activities. **186** showed a maximum TON_H,Cu_ of 305, whereas **187** (TON_H,Cu_ of 25) and **188** (TON_H,Cu_ of 29) possessed very low activity. This could be attributed to the shortened excited state lifetimes resulting from the rapid depopulation of diamine‐localized excited states. To achieve a longer excited state lifetime, Karnahl et al. further prepared **189**–**190** by replacing the phenazine with an anthracene moiety [233]. Compared to **186**, **189**–**190** demonstrated improved light harvesting and excited state properties with the lifetime up to 4 µs (for **176**) in acetonitrile. The TONs followed the order of **188** (29) < **189** (39) < **190** (51) < **186** (64). By analyzing their electrochemical behaviors, the much less negative first reduction potentials of **188**–**190** (about −1.1 V) compared to **186** (−2.1 V) might prevent effective reduction of the primary active species of the iron catalyst during catalysis. Therefore, the strategy of modification on the N^N ligand for improving PHP was buried.

Beller et al. explored the structural effects of the N^N ligands on the PHP activity, focusing on the steric and electronic effects of substituents at the 2,9‐ and 4,7‐positions of phenanthroline [178]. Thixantphos featuring large planar structure of phosphorus resulted in higher or comparable H_2_ production compared to those with Xantphos. The steric factors at the 2,9‐positions of the N^N ligand and the use of electron‐withdrawing groups were found to be beneficial for PHP. Through fine‐tuning the N^N ligands, the designed PS **191** achieved improved TONs of up to 1388 in 11 h. Park et al. investigated the structure–property–catalytic activity correlation of 2,9‐dimethyl‐1,10‐phenanthroline ligands with different substituents at the 4,7‐positions [179]. Among **192**–**196**, the tertiary amine substitution on the N^N ligand produced intra‐ligand charge transfer (ILCT) excited states that effectively prevented the distortion of coordination axes, leading to higher luminescence QY and longer lifetimes. Thus, **193** and **196** showed outstanding absorption (*ε* = 29 400 and 34 700 M^−1^ cm^−1^, respectively) and long excited‐state lifetime (120 and 88 µs, respectively) compared to other complexes showing MLCT behavior, suggesting their superior performance as PSs. Notably, **196** with TPA‐substituted phenanthroline ligands demonstrated an unprecedentedly high TON of 19 000 and a TOF of 1800 h^−1^ in combination with TEA as SED and colloidal Pt as catalyst.

Tschierlei et al. discovered that placing 4‐methoxyphenyl substituents at the 4,7‐position of the N^N ligand (**198**) rather than the 5,6‐position (**197**) led to a more than twofold increase in absorption across the 300–500 nm range, and the *τ*_em_ value was increased by a factor of 60 (628 ns with *Φ*_em_ = 2.1% for **198** vs 11 ns with *Φ*_em_ = 0.16% for **197**) [180]. In comparison with **197**, the 4,7‐substituent induced a more in‐plane orientation for **198**, resulting in more pronounced MLCT and ligand‐centered charge transfer (LCCT) excitations. **197** and **198** displayed similar reduction potentials *E*_1/2_^red^ of −2.11 and −2.08 V in acetonitrile, and their performance in photocatalytic water splitting and CO_2_ reduction were studied. In the system with [Fe_3_(CO)_12_] as catalyst and TEA as SED, **198** (TON = 613) yielded a seven‐fold higher H_2_ generation activity than **197** (TON = 87). Moreover, in the presence of **[Re_2_Cl_2_]** as catalyst and a combination of BIH and TEA as SED, **198** (TON_CO_ = 175) achieved three‐fold higher CO_2_ reduction activity than **197** (TON_CO_ = 51). This work underlined that the position of substituent can exert a great influence on both the photophysical and photocatalytic performance.

By coupling **199**–**203** as PSs and iron cyclopentadienone as CO_2_‐reduction catalyst in a mixture of *N*‐methyl‐2‐pyrrolidone (NMP)‐TEOA‐BIH, Beller et al. demonstrated the substituent effects of the N^N ligands on the photocatalytic CO_2_ reduction activity [192]. **199** achieved a maximum TON_CO_ of 487 (5 h) with a selectivity of 99%. The reductive quenching mechanism dominated the CO_2_ photoreduction reaction. Recently, in a system developed by Fontecave [234], the PS **192**, combined with Fe‐salophen as the catalyst and BIH as the SED, exhibited high photocatalytic CO_2_ reduction performances with a maximum TON_CO_ of 1600, a maximum initial TOF of 1700 h^−1^ and 96% CO/H_2_ selectivity.

However, most research on CO_2_ reduction with Cu(I) PSs is conducted in organic solvents (like DMF, THF or acetonitrile). For practical applications, water‐based photocatalytic systems are superior due to water's abundance, environmental safety, and ability to promote proton/electron transfer. Recently, Sakai et al. designed a water‐soluble trianionic heteroleptic Cu(I) PS (**204**) preserving both strong luminescence and a long‐lived excited state in aqueous solutions [193]. The absorption spectrum of **204** featured a ligand‐centered π–π* absorption band in the UV region and a typical broad absorption band centered at 390 nm (*ε* = 3.7 × 10^3^ M^−1^ cm^−1^), attributed to ^1^MLCT transition with a significant contribution of ILCT transitions. **204** showed long‐lived excited state emission in both organic solvent and solid state, and even in aqueous media (λ_em_ = 565 nm, *Φ*_em_ = 2%, *τ* = 1.1 µs; in CO_2_‐saturated 0.1 M NaHCO_3_ buffer, pH = 6.7). This was attributed to the hydrophilic sulfonate groups in **204** that contributed to the formation of hydrogen bonding, blocking solvent water molecules that would promote the non‐radiative decay process. The **204**‐driven CO_2_ reduction, in the presence of a water‐soluble Co‐porphyrin (CoTMPyP) catalyst and ascorbate (AscHNa) SED, achieved the highest catalytic activity with TON_CO_ = 2680 (4 h) and TOF_CO_ = 2600 h^−1^ with Sel_CO2_ = 77%. The outstanding catalytic performance was attributed to the multielectron charge transfer character of the CO_2_ reduction catalyst, permitting CO rapid release via reducing Co^II^ to Co^I^ by intermolecular electron transfer. Furthermore, by shifting the *N*‐methyl group of CoTMPyP from the *para*‐position to the *ortho*‐position, a substantial improvement in Sel_CO2_ (90%) and TON_CO_ (4000, 12 h) was achieved, accompanied with a decreased TOF_CO_ = 1170 h^−1^ [235]. Benefiting from the attractive long‐lived ^3^MLCT feature of **204** ^3−^ in aqueous media (*τ*_ave_ = 0.89 µs in H_2_O) and its sufficient driving force for CO_2_ photoreduction, the authors further constructed a 1:2 ion‐pair adduct [**204(HM)_2_**]^+^ based on the anionic **204** ^3−^ and cationic hexamethonium (HM^2+^) via ionic interactions [236]. [**204(HM)_2_**]^+^ effectively suppressed the ligand dissociation, resulting in excellent photophysical properties and photocatalytic performance through the inhibition of water‐derived non‐radiative decay of the photoexcited state. [**204(HM)_2_**]^+^ showed enhanced MLCT absorption and emission (9‐fold enhancement) in water, and the lifetime was 15 µs. The ion‐paired [**204(HM)_2_**]^+^ showed a maximum catalytic rate (TOF_CO_ = 150 h^−1^) more than twice higher than that of free **204** ^3−^ (TOF_CO_ = 63 h^−1^). This demonstrates that suppressing non‐radiative decay significantly boosts photocatalytic efficiency. As the first successful example of ion‐pair to enhance photocatalysis, this work provides a promising new approach for advanced photocatalytic reaction control.

Tsubomura et al. discovered that elongating the carbon chains between the two phosphine donors can increase the P‐Cu‐P chelation angles, which in turn improves both the photosensitizing abilities and emission lifetimes of Cu(I) complexes [194]. Increasing the bite angles could eliminate the steric hindrance of phenyl groups on the phosphine ligands, block solvent access to the Cu(I) center and stabilize the one‐electron reduced species, thereby promoting the electron transfer process. As a result, **207** showed a better CO_2_ photoreduction performance than **205** and **206**. The TON_CO_ values were in the order of **205** (230, TOF of 7.3 min^−1^) < **206** (560, TOF of 27 min^−1^) < **207** (580, TOF of 65 min^−1^).

In addition to the aforementioned Cu(I) PSs, through direct coordination of Cu^2+^ to organic dyes purpurin to form Cu(II)‐purpurin **208**, this approach significantly promoted the photocatalytic activity. When coupled with a Fe‐porphyrin as the catalyst and BIH as the SED, **208** dramatically boosted the CO_2_ reduction activity (>50‐fold) compared to its organic dye component, achieving a TON_CO_ of 16109 and a TOF_max_ of 7650 h^−1^ with a 95% selectivity (CO vs H_2_) at 100 mM concentration of BIH [195]. Compared to purpurin (*ε* = 9.94 × 10^3^ M^−1^ cm^−1^), **208** (*ε* = 3.85 × 10^4^ M^−1^ cm^−1^) displayed much stronger visible absorption and enhanced charge transfer character in DMF solution. Additionally, **208** provided a much more negative reduction potential (−1.75 V) than purpurin (−1.21 V), indicating that **208** had a larger driving force for reduction reaction. The CO_2_ reduction pathways are shown in Figure 8. Upon excitation, **208** underwent a reductive quenching pathway with a photochemical process of [((CO_2_)_2_L)_2_Cu^II^]^4–^ → [((CO_2_)_2_L)_2_Cu^II^]^4–^* → [((CO_2_)_2_L)_2_Cu^II^]^5–^. This reduced [((CO_2_)_2_L)_2_CuI]^5–^ (−1.75 V) then transferred an electron to the Fe catalyst and subsequently mediated the CO_2_ reduction reaction. Notably, at a low concentration of BIH (0.1 mM), CO_2_ reduction proceeded through oxidative quenching of (L_2_)^+^CuI* (−1.61 V) due to the higher reduction potential of [L_2_Cu^II^]^–^ (L = PP^2–^) (−1.05 V) than that of Fe(I) catalyst (*E*_1/2_ = −1.55 V). With the highly active **208**, Han et al. further explored its activity for H_2_ production in the presence of TEA and BIH as SED and a molecular Ni catalyst [237]. Under optimal conditions, **208** achieved TON_H2_ of 1180 and TOF of 9.8 in 120 h. The activity was comparable to that of the classic heteroleptic Cu(I) PSs.

#### 3.2.2 Zn(II)‐Based Photosensitizers

**FIGURE 9:**
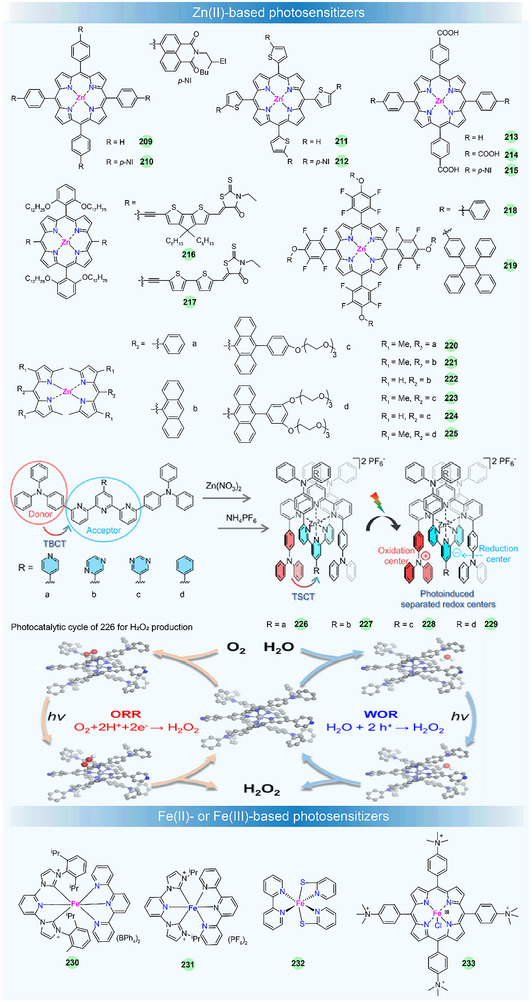
Chemical structures of Zn(II)‐, Fe(II)‐, and Fe(III)‐based photosensitizers. The photocatalytic cycle of **226** for H_2_O_2_ production is reproduced with permission [240]. Copyright 2025, American Chemical Society.

In contrast to the MLCT‐driven photoinduced electron transfer of the above tetrahedral Cu(I) complexes, their isoelectronic Zn(II) counterparts display ligand‐centered excited states. This is because Zn(II) has a higher oxidation state energy than Cu(I), which shifts the MLCT absorption to the blue (higher energy) and lowers the energy of ligand‐based states [238]. Small‐molecule Zn(II) porphyrins are commonly used as PSs due to their strong light absorption and ability to facilitate photoinduced electron transfer [239]. Over the past few years, our group has embarked on developing novel Zn(II)‐porphyrins and studying their PHP behaviors. A series of Zn‐porphyrin‐based photocatalysts were synthesized and their high photocatalytic efficiencies were recorded (Figure 9). Moreover, the integration of various chromophores at the *meso*‐positions of the porphyrin ring greatly fine‐tuned their PHP activities.

For instance, our research reveals that the linear substitution of dinaphthalimide (NI) moieties at the *meso*‐position of the porphyrin core leads to a significant improvement in the light absorption, photoexcited state stability, photoinduced charge separation efficiency, and photostability of porphyrin PSs, thereby enhancing PHP [181, 241]. This is because an efficient energy transfer occurs between the NI donor and porphyrin acceptor, driven by the spectral overlap between the emission profile of NI and the absorption of porphyrin moiety. **210** and **212** containing NI groups showed much higher catalytic activity than **209** and **211**. Compared with benzene, the thiophene linkage enhanced both energy transfer efficiency and well‐ordered π‐stacking between porphyrin molecules, further facilitating electron transport from the excited porphyrins to the proton reduction catalyst, leading to a 2.9‐fold higher HER of **212** (4.28 mmol g^−1^ h^−1^) than **210** (1.50 mmol g^−1^ h^−1^). Additionally, **212** was much more photostable than **210** and sustained hydrogen evolution for up to 50 h. Furthermore, in a homogeneous phosphate buffer/THF system containing chloropyridinecobaloxime (CoPyCl) as cocatalyst and AA as SED, **215** bearing two NI chromophores and two –COOH groups, exhibited a significantly higher HER of 35.70 mmol g^−1^ h^−1^ (with a TON of 5958 and AQY of 10.01%), which was 8‐fold higher than that of **213** which lacked the NI groups (4.64 mmol g^−1^ h^−1^, TON of 1397 and AQY of 1.30%) and 15‐fold higher than that of **214** (2.43 mmol g^−1^ h^−1^, TON of 562 and AQY of 1.00%) [182]. At the same time, the –COOH substituents accelerated electron transfer from the porphyrin group to the cationic Co(III) through electrostatic interactions.

Although the Zn‐porphyrins discussed above demonstrated significant improvements in PHP efficiency, their absorption remains confined to the UV‐visible range. To broaden the light‐harvesting range in the visible light region, we further designed **216** and **217** with variable π‐linkers to enhance PHP [183]. These Zn‐porphyrins exhibited solid absorption across the 300–1100 nm range, confirming their visible‐to‐near‐infrared light‐harvesting capability. Compared to **209**, **216** showed more redshifted absorption and better photoinduced charge separation than **217** owing to the enhanced intramolecular ET from the porphyrin donor to the 3‐ethylrhodanine acceptor moieties. As a result, **216** yielded a HER rate of 1.80 mmol g^−1^ h^−1^, which was 4.5‐fold higher than that of **217** (0.40 mmol g^−1^ h^−1^).

Photoluminescence aggregation‐caused quenching (ACQ) is commonly observed in porphyrins in the solid state, resulting from the strong π‐π stacking interactions of their planar porphyrin cores. The ACQ activates the nonradiative decay channels, with the consequence of further shortening the electron lifetimes of photoexcited states, thus resulting in low PLQY and consequently inferior PHP performance. To address this issue, we recently developed a strategy to conjugate a propeller‐shaped tetraphenylene (TPE) chromophore to the Zn‐porphyrin ring [184]. The incorporation of TPE molecules, which exhibited aggregation‐induced emission, effectively abolished the strong π‐π stacking, thus preventing ACQ. Furthermore, FRET from TPE to porphyrin in **219** enhanced the light‐harvesting ability, extended the electron lifetime by stabilizing the photoexcited singlet state, and enabled efficient charge separation and fast migration in the photocatalytic system. As a result, an excellent photocatalytic HER of 56.20 mmol g^−1^ h^−1^ was recorded for **219**, which was 94‐fold higher than that of **218** (0.60 mmol g^−1^ h^−1^) without the TPE groups.

However, very few Zn(II) porphyrins have been explored as PSs for CO_2_ photoreduction due to limited light‐harvesting ability or poor photochemical stability [242, 243]. Conventional Zn–dipyrrin complexes exhibit energy dissipation through phenyl rotation and excitonic coupling, inherent to their flexible structures. These pathways can be blocked by increasing the torsional resistance and maintaining orthogonal configuration [244]. Recently, Zhang et al. designed a library of symmetric Zn–dipyrrin complexes (**220**–**226**) with identical chromophores and used them for CO_2_ photoreduction for the first time [196]. Introducing anthryl derivatives in **220**, **223,** and **225** increased the torsional resistance, kept an orthogonal molecular configuration, and improved their excited state properties. **223**–**225** were also modified with polyethylene glycol (PEG) to improve water solubility. As a result, these symmetric Zn–dipyrrin complexes exhibited the structural characteristics for an efficient symmetry‐breaking charge transfer (SBCT) process, showing strong sensitizing ability for solar energy utilization. All these Zn‐based PSs showed strong absorption peak at ∼500 nm with *ε* > 120 000 M^−1^ cm^−1^. The complexes (**220**–**225**) exhibited locally excited (^1^LE) state emission at ∼515 nm and the PLQY of < 0.1% in acetonitrile, providing the possibility for efficient ICT. Photocatalytic experiments were carried out in a mixed solvent of H_2_O/CH_3_CN in the presence of BIH as SED and [Fe(qpy)(H_2_O)_2_]^2+^ as catalyst. The sensitizing ability of these Zn–dipyrrin followed the order of **225** *>* **223** *>* **221** *>* **222** ≈ **224** *>* **220**. Remarkably, the catalytic system with **225** exhibited outstanding performance with a TON_CO_ of > 20000, AQY_CO_ of 29.7% at 488 nm, and nearly 100% selectivity for CO, highly superior to that of typical noble‐metal PSs **122** and **130** under similar conditions. The longest triplet excited state lifetime (164.5 µs) and high triplet state quantum yield (54%) afforded **225** with high performance for CO_2_ reduction. The experimental results confirmed that the special geometry of Zn–dipyrrin facilitated ICT upon visible‐light excitation, producing long‐lived reduced PS. These species drove consecutive intermolecular electron transfers, efficiently sensitizing Fe‐catalyst and improving CO_2_‐to‐CO conversion. This work demonstrates a new strategy for enhancing CO_2_ photoreduction by rationally designing low‐cost PSs using more abundant metals.

In 2025, Liu et al. reported a class of novel Zn‐terpyridine complexes **226**–**229** with head‐to‐tail geometry and first demonstrated the single‐molecule photocatalytic system for H_2_O_2_ generation [240]. As expected, the pseudo‐octahedral geometry and head‐to‐tail dimeric structure of the complexes promote through‐space charge‐transfer transition under photoexcitation, creating both long‐lived excited states and separate redox centers. Moreover, the particularly low exciton binding energies render these complexes efficient single molecular photocatalysts for the overall photosynthetic production of H_2_O_2_, achieving a high H_2_O_2_ evolution rate (8862 µmol g^−1^ h^−1^) and 0.43% solar‐to‐chemical conversion efficiency for **226** in pure H_2_O under an air atmosphere. The experimental and theoretical results demonstrated the co‐existence of four‐electron oxygen reduction reaction (ORR, O_2_ + 2H^+^ + 2e^–^ → H_2_O_2_)) and two‐electron water oxidation reaction (WOR, 2H_2_O → H_2_O_2_ + 2H^+^ + 2e^–^) pathways in the single‐component catalyst. The reaction mechanism of the overall photosynthesis of H_2_O_2_ is shown in Figure 9. Upon light irradiation, **226** exhibited ultrafast photoinduced charge separation, enabling the immediate binding of water and oxygen at the catalytic sites. The photogenerated electrons (e^–^) on the triphenylamine units drove the ORR to produce H_2_O_2_ through the *OOH intermediate, while the photogenerated holes located on the terpyridine moieties triggered the WOR to generate H_2_O_2_ through the *OH intermediate. This work establishes a new framework for photocatalytic energy conversion and offers novel perspective for advancing molecular photocatalyst design.

#### 3.2.3 Fe(II)‐ or Fe(III)‐Based Photosensitizers

Iron(II) containing 3*d* ^6^ valence electron configuration is isoelectronic with typical noble metals such as Ru(II), Ir(III), Re(I), etc., and exhibits excellent abilities to excite electrons upon absorbing visible light [245]. However, it remains challenging to develop iron‐based complexes with long excited state lifetimes due to the intrinsic differences in the nature of the 3d versus 4d and 5d orbitals. Specifically, iron complexes experience very fast deactivation of the photoactive ^1,3^MLCT states to inactive metal‐centered states [246]. This contrasts sharply with Ru(II) and Ir(III) complexes, where a large energy gap between the ^1,3^MLCT and metal‐centered states results in significantly longer‐lived active MLCT states. Therefore, choosing suitable ligands with σ‐donation properties to modulate the ligand‐field strength of iron(II) centers and thus lowering the energy of MLCT excited states is an effective strategy to prolong the excited state lifetime.

Iron(II) N‐heterocyclic carbene (NHC) complexes are promising PSs [247, 248]. Their strong metal‐carbene bond increases ligand‐field splitting, destabilizing the MC states. Thus, Fe‐NHC type PSs exhibit long excited‐state lifetimes and high charge transfer abilities. For the first time, Bauer et al. demonstrated the potential of using homo‐ and heteroleptic Fe‐NHC complexes as PSs in PHP (Figure 9) [249]. The bis‐NHC ligand stabilized the photoactive MLCT states and ensured photostability, while the terpyridine ligand provided intense visible light absorption. The PHP activity was evaluated in the system of CH_3_CN/H_2_O containing Fe PSs, K_2_PtCl_4,_ and Et_3_N. The observed activities of **230** (TON = 3) and **231** (TON = 4) were about 1:17 compared to the established **130** (TON = 61), making these Fe PSs appear uncompetitive [250]. However, the significant cost advantage (1:45 price ratio) and superior stability could offset the reduced activity in practical applications.

He et al. reported a mononuclear iron pyridine‐thiolate complex **232** that functioned as both PS and catalyst for visible‐light‐driven CO_2_ reduction, generating CO and formate (HCOO^−^) as products, with a total TON_CO_ of 46, TOF of 11.5 h^−1,^ and AQY of 8.4% after 4 h of light irradiation [197], which were much higher than the benchmark Re photocatalyst **173** (TON_CO_ of 2, 4 h) under identical experimental conditions. The strong *σ*‐donation properties of the pyridine‐thiolate ligand increased the electron density and ligand‐field strength of the Fe(II) center, facilitating the construction of Fe‐based complexes with long excited state lifetimes. Quenching experiments indicated that high photocatalytic activity stemmed from rapid intramolecular quenching. Meanwhile, DFT calculations and electrochemical results demonstrated that protonation of **209** was an indispensable step for photocatalytic CO_2_ reduction. Robert et al. showed that a substituted tetraphenyl Fe(III)‐porphyrin (**233**) with *para*‐positioned trimethylammonio groups served as a homogeneous molecular catalyst for visible‐light‐driven CO_2_ reduction in organic media without a sensitizer [251]. **233** achieved a TON_CO_ of 101 with 100% selectivity after 102 h of continuous irradiation, demonstrating its robustness.

### 3.3 Bi‐Metal‐ or Multi‐Metal‐Based Photosensitizers

**FIGURE 10:**
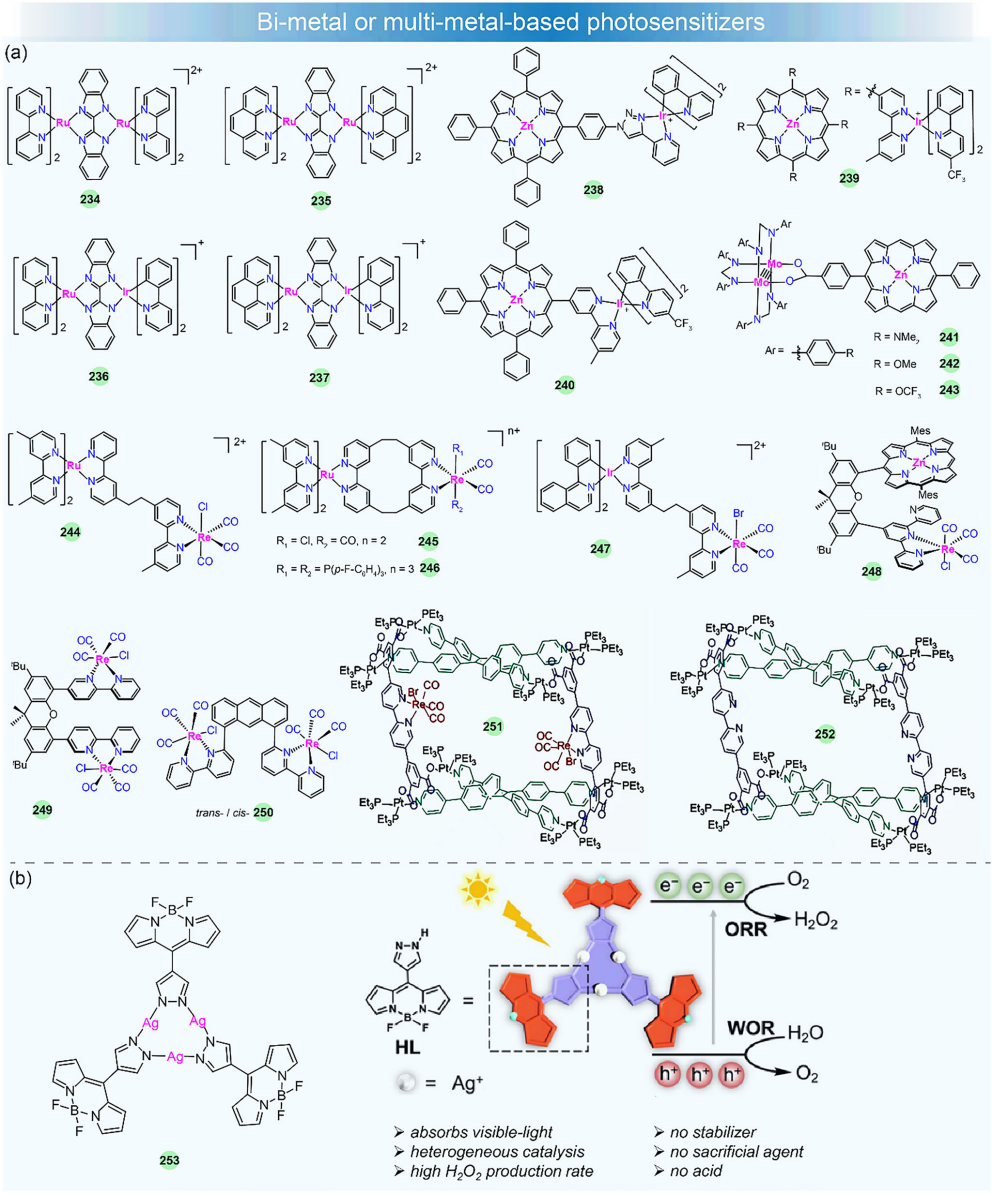
(a) Chemical structures of bi‐metal or multi‐metal‐based photosensitizers. (b) Chemical structure of **251** and its visible light‐driven photosynthesis of H_2_O_2_. (ORR and WOR mean the oxygen reduction reaction and water oxidation reaction). (b) Reproduced with permission [254]. Copyright 2024, Royal Society of Chemistry.

In photocatalysis, besides mononuclear systems, exploring the synergistic effects of bi‐metal or multi‐metal complexes as PSs is interesting. Integrating multiple metal centers within a single molecular or supramolecular framework enables more efficient charge separation, electron accumulation, thus improving catalytic performance. In dinuclear metal complexes, one complex serving as an efficient PS is covalently linked to the second complex acting as a photocatalyst, to construct a PS‐catalyst dyad capable of performing proton or CO_2_ reduction. Ishitani's group has developed dyad catalysts for the photochemical CO_2_ reduction [252]. Luo et al. has reviewed the recent advances of all‐abundant‐element PS‐catalyst dyads for PHP via intramolecular processes [253]. Therefore, this section will primarily center on the latest developments of novel bi‐metal or multi‐metal‐based photocatalysts and their performance in PHP and CO_2_ reduction (Figure 10).

Su et al. prepared four butterfly‐like dyads **234**–**237** bearing different ligands for PHP [185]. The absorption and luminescence of **234**–**235** were dominated by MLCT transitions. While in **236**–**237**, energy transfer occurred from the higher‐energy level MLCT state of Ir(III) chromophores to the lower‐energy level MLCT state of Ru(II) chromophores, thus resulting in similar deep red emission which originated from Ru(II) units with prolonged decay lifetimes. The PHP activities of these complexes were assessed in DMSO/H_2_O system containing Pt as catalyst and TEA as SED. Heteronuclear **236** showed the highest H_2_ production efficiency after 80 h of continuous production with a TON of 1088.

Conjugating a heavy metal complex to the porphyrin macrocycle significantly extended the light‐harvesting ability across the entire UV‐visible region while improving the photoexcited state lifetime. For example, in **Ir**–**Zn** dyads featuring a long‐lived Ir(III) complex covalently linked to a Zn‐porphyrin, the triplet state of the Zn‐porphyrin could be stabilized by intramolecular energy transfer, which promoted electron transfer from the photo‐excited triplet Ir(III) and porphyrin moieties to the catalyst, thus improving the catalytic efficiency. Zhu et al. designed **238** and demonstrated efficient FRET from Ir to porphyrin [186]. As expected, **238** achieved a cocatalyst‐free photocatalytic HER of 1.42 mmol g^−1^ h^−1^, which was ca. 71‐fold higher than that of the control Zn‐porphyrin (0.02 mmol g^−1^ h^−1^) and Ir complex (0.03 mmol g^−1^ h^−1^). Our group has reported **239**–**240** for PHP [187]. In the presence of CoPyCl as co‐catalyst, **239** displayed the highest HER of 16.12 mmol g^−1^ h^−1^ after 5 h of light irradiation, which was 2.73 times higher than that of **240** (5.90 mmol g^−1^ h^−1^).

Recently, Liu et al. developed three photocatalysts **241**–**243** by integrating Zn‐porphyrin with a quadruply‐bonded Mo_2_ unit for PHP [188]. By varying the *para‐*substituents on the aryl groups of the N,N′‐diarylformamidinate (DArF) ancillary ligands: from electron‐donating N(CH_3_)_2_ to electron‐withdrawing OCF_3_ group, the HER followed the order of **241** (50 mmol g^−1^ h^−1^ with TON of 640) > **242** (48 mmol g^−1^ h^−1^ with TON of 586) > **243** (31 mmol g^−1^ h^−1^ with TON of 459). Mechanistic investigations revealed that both the Mo_2_ unit and Zn‐porphyrin were excited under visible‐light irradiation, creating multiple pathways for electron transfer from the donor (Mo_2_) to the porphyrin acceptor. The photoreduced Zn‐porphyrin was likely the active intermediate responsible for proton reduction. The electron‐donating substituents raised the *δ* orbital energy, increasing the photoinduced electron transfer driving force, thus affording higher catalytic performance.

Re complexes based on the [Re(N^N)(CO)_3_Cl] motif are well‐known homogeneous catalysts for light‐driven CO_2_ to CO reduction. Recently, many research efforts have been devoted to designing novel intramolecular catalysts with one or more visible‐light‐absorbing fragments covalently attached to a Re^I^ unit. For example, in the presence of 2‐(1,3‐dimethyl‐2,3‐dihydro‐1H‐benzimidazol‐2‐yl)benzoic acid (BI(CO_2_H)H) as SED, Ishitani et al. reported that dyad (**244**) served as an efficient and durable catalyst for CO_2_ reduction in aqueous solution under visible‐light irradiation, with AQY of 13% and TON of 130 [255]. Compared to **244** with only one ethylene chain in the bridging ligand, the authors proved that **245**–**246** with ethylene‐bridged Ru–Re pair exhibited stronger electronic interactions, which increased the oxidation power of the excited Ru moiety, achieving an improvement in the photocatalytic ability (AQY_CO_ = 16%, TON_CO_ = 204 for **245**; AQY_CO_ = 15%, TON_CO_ = 212 for **246**) [198]. Using Ir with a relatively intense absorption band and longer lifetime than Ru units, the authors further constructed dyad **247** that showed a dramatically enhanced photocatalytic activity for reducing CO_2_ to CO with 99.9% selectivity (TON_CO_ = 1700) by utilization of BIH as the SED [199].

In contrast to the flexible connections discussed above, Schwalbe et al. prepared a rigid **248** dyad, which provided two different metal coordination sites in close proximity. This dyad consisted of a Zn‐porphyrin unit as the PS and a Re unit as the catalyst, which could effectively disrupt the conjugation between the two functional units while preserving efficient visible‐light absorption and facilitating energy/electron transfer between the PS and the catalyst [200]. As a result, **248** showed a TON_CO_ of 195 after 24 h of light illumination using TEOA/BIH as SED. Recently, the authors explored a new xanthene‐bridged **Re‐Re** dyad **249** showing two isomers, *cis* and *trans* [201]. But the *cis* isomer could not be obtained. The photocatalytic investigations indicated a significant increase of the catalytic performance for *trans*‐**249** (TON_CO_ of ∼300 and TOF of 20 h^−1^) compared to the mononuclear parent compound, which could be rationalized by the suppression of deactivation pathways and a cooperative mechanism where one metal center functioned as a PS to support the second one as a catalytically active metal.

In a similar fashion, Jurss et al. tethered two bpy ligands to an anthracene backbone [202]. They successfully isolated the two isomers and demonstrated their equal catalytic performance. At a low concentration (0.05 mM of catalyst), *cis*‐ and *trans*‐**250** had comparable reactivity (158–128 TOF h^−1^, 105–95 TON), substantially outperforming the mononuclear complexes (43–17 TOF h^−1^, 34–16 TON) where intermolecular collisions were fewer. With increasing concentration, TOF and TON values diminished rapidly for the *trans*‐conformer compared to the *cis‐*conformer, suggesting *trans*‐conformer with greater vulnerability to intermolecular catalyst–catalyst deactivation. At a very low concentration (1 nM), *trans*‐**250** gave a remarkable TON of 40 000 for CO.

Based on metal‐coordination‐driven self‐assembly, Zhang et al. designed two isoreticular TPE‐based metallacages **251**–**252** for PHP, where TPE, tetracarboxylic, and *cis*‐Pt(PEt_3_)(OTf)_2_ acted as the faces, pillars, and vertices, respectively [189]. Incorporation of TPE and Re complex into the metallacage offered a high HER of 1707 µmol g^−1^ h^−1^ for metallacage **251** (TON of 157 and AQY_CO_ of 6.53 × 10^−3^) upon 12 h of light irradiation, which was about 3‐fold higher than that for metallacage **252** without Re complex. Femtosecond transient absorption and DFT calculations revealed that the efficient and directional electron transfer from the TPE faces to the Re catalytic centers contributed to the highly efficient photocatalytic performance. Notably, the metal nodes (Pt) in metallacage **252** served as the key catalytic center for PHP. This work provides an approach to combine multiple photosensitive and catalytic ligands within a single multicomponent metallacage, improving the electron transfer efficiency to achieve efficient PHP and guiding the future design of metallacages for photocatalytic applications.

Developing metal complexes for solar‐driven hydrogen peroxide (H_2_O_2_) production from pure water and oxygen without any additives (e.g., acids, co‐catalysts, and sacrificial agents), remains highly challenging. Recently, Li et al. designed a boron dipyrromethene (BODIPY)‐based cyclic trinuclear silver complex (**253**), and explored its photocatalytic performance in H_2_O_2_ production (Figure 10b) [254]. The BODIPY motif greatly improved the visible‐light absorption (*ε* = ∼7.4 × 10^4^ M^−1^ cm^−1^) and photo‐induced charge‐separation in **253**. Under illumination, BODIPY served as oxygen reduction sites while silver ions acted as water oxidation sites that allowed **253** to photosynthesize H_2_O_2_ from pure water or sea water without additives at a rate of 183.7 and 192.3 µM h^−1^, respectively.

### 3.4 Metal‐Based Polymer Photosensitizers

In photocatalysis, noble metal or metal nanoparticles (like Ir, Pt, Pd) as a cocatalyst can enhance the photocatalytic efficiency, which are usually mixed directly with another photocatalyst [256, 257]. While efficiency is enhanced, this approach struggles with uncontrolled metal‐photocatalyst interactions and weak charge transfer characters. Moreover, metal dispersion in organic/water solvents creates toxicity issues [258]. To address these challenges, introducing a metal complex comonomer into the polymer skeleton to form metallopolymers can be a promising strategy. This approach not only improves the photocatalytic efficiency but also addresses the corrosion issue of the metal complex. Furthermore, fine structural modifications of metallopolymers can efficiently enhance the photocatalytic activity and stability. The metallopolymers can be constructed by incorporating monomer building blocks with metal ions via covalent or noncovalent bonding approaches. Among various noncovalent interactions, metal–ligand coordination bonds exhibit high directionality and strength, comparable to covalent bonds. In this part, we will describe in detail the two types of metallopolymers which act as both PSs and photocatalysts in photocatalysis.

#### 3.4.1 Covalently Linked Metal‐Based Polymers

**FIGURE 11:**
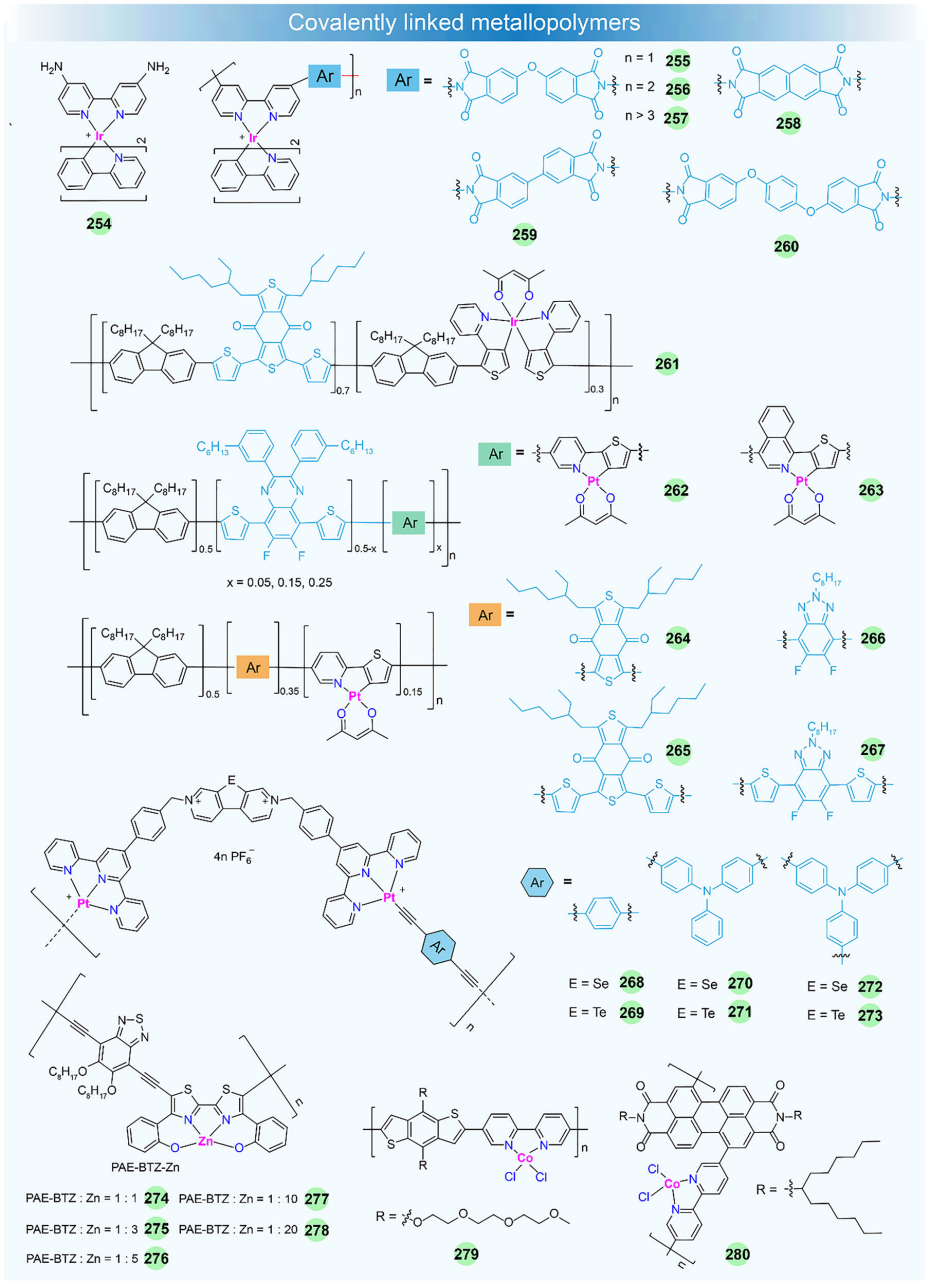
Chemical structures of covalently linked metallopolymers.

Covalently linked metallopolymers offer improved durability, tunable electronic properties, and enhanced photocatalytic activity, compared to small‐molecule metal complexes. The fine structural modifications of Ir PSs could efficiently improve the photocatalytic activity and stability. However, these PSs with high photocatalytic activity are prone to degradation due to the rupture of Ir‐N (N^N) bonds during photocatalysis [259]. To achieve a simultaneous increase in stability and activity of classical Ir complex for PHP, Deng et al. designed and synthesized a series of oligomers **255**–**257** by copolymerization of **254** with 4,4′‐oxydiphthalic anhydride (Figure 11) [260]. Increasing polymerization degree narrowed the energy gap, reducing the electron excitation energy and improving the utilization of visible light, which ultimately enhances photocatalysis. The photocatalytic tests were carried out under visible light in a DMF/H_2_O system with K_2_PtCl_4_ as the catalyst and TEA as the SED. Among these oligomers, **255** and **256** showed the best photocatalytic activity of 162 055.1 µmol·g^−1^ (TON of 365) with a lifetime up to 676 h, which were about 1.3 times higher and 38 times longer than that of the classical Ir complex **254**. Further increasing the degree of polymerization led to decreased photocatalytic lifetime and activity. The hydrogen production of **257** (n > 3) was 86 568.1 µmol·g^−1^ with a lifetime of 290 h. This result indicated that an oligomer chain with suitable length provided a large steric hindrance, endowing **255** and **256** with a significantly prolonged lifetime. The decreased lifetime of **257** resulted from the complicated macromolecular decomposition mechanism and reduced structural integrity. This work demonstrates a simple approach for constructing oligomeric Ir PSs with both long lifetime and high activity, laying the foundation for future design of efficient PSs.

To further enhance the oligomeric structural effect on photocatalysis, oligomeric Ir PSs **258**–**260** were designed through structural modifications of the anhydride linkers [261]. Experiment and theoretical calculations showed that the oligomeric PS molecules aggregated, enabling neighboring oligomer coupling through conformational changes. This significantly affected the electron transfer pathways, resulting in reduced fluorescence emission, broader and stronger light absorption, and enhanced photocurrent response. All oligomers demonstrated higher photocatalytic activity and longer photocatalytic lifetime than **254**, following the order of **257** (1727.4 µmol with a lifetime of 696 h) > **260** (1470.3 µmol with a lifetime of 520 h) > **259** (1426.0 µmol with a lifetime of 172 h) > **258** (1112.9 µmol with a lifetime of 192 h) > **254** (967.4 µmol with a lifetime of 21 h). Compared to rigid linkers, flexible linkers better facilitated intramolecular and intermolecular electron transfer, boosting photocatalytic activity. The findings show that moderate steric hindrance enhances PSs stability while maintaining activity. This work is the first to reveal the aggregation effect of oligomeric Ir PSs on their photocatalytic performance, providing a theoretical basis for developing high‐performance photocatalysts.

Generally, conjugated polymers are hydrophobic and poorly dispersed in water, so organic solvents are needed to boost their dispersibility for the photocatalytic reaction. Recently, studies have focused on developing hydrophilic polymers by using polymer dots (Pdots) or polyelectrolytes, which allow conjugated polymers to work in photocatalytic systems without organic solvents [262]. Constructing conjugated polymer‐based Pdots has been a promising way to improve their H_2_ evolution efficiency as photocatalysts [263]. For example, Chou et al. designed an Ir‐based polymer **261** by covalently incorporating Ir complex into the polymer backbone, and then further converting it into Pdots for PHP for the first time [264]. In the structure, dioctyl‐9H‐fluorene was the donor, thienyl‐benzodithiophene‐dione was the acceptor, and Ir complex acted as the cocatalyst. Compared to the pristine conjugated polymers, Ir‐based polymer exhibited similar absorption but quenched emission properties, indicating the suppressed recombination of electron‐hole pairs in **261** due to the effective charge carrier separation between the Ir complex and donor group. Under the visible light, the resulting Pdot **261** showed a 22‐fold enhancement in catalytic activity (2666 µmol g^−1^) than the pure polymer Pdots (123 µmol g^−1^) without Ir complex. The result strongly implies that inserting Ir(III)‐complex into the conjugated polymers is beneficial for enhancing PHP, which can inspire more improvements and new ways to design high‐performance photocatalysts for producing clean and renewable energy.

Chou et al. also developed two kinds of Pt‐based Pdots **262**–**263** as promising photocatalysts for PHP [265]. The introduction of the planar Pt complex did not significantly alter the LUMO/HOMO positions and optical bandgaps but caused a slight redshift of the emission peak and a decrease in emission lifetime from 1.57 to 0.6 ns. The PHP tests were carried out in water with diethylamine as the SED, and **262_15_** Pdots exhibited the highest HER of 12.7 ± 0.6 mmol g^−1^ h^−1^ at 15 mol% Pt content, which was approximately 12 times higher than that of pure Pdots (1.3 ± 0.1 mmol g^−1^ h^−1^). Notably, when the amount of Pt complex reached 25 mol%, the HER dropped to 7.7 ± 0.2 mmol h^−1^ g^−1^, likely because the metal cocatalyst became saturated. The HER of **263_15_** Pdots was 11.1 ± 0.3 mmol g^−1^ h^−1^. The higher HER of **262_15_** Pdots than **263_15_** may be attributed to the more planarized structure of **262** with smaller dihedral angle, which will increase charge carrier mobility and decrease Coulomb binding energy for separating electron/hole pairs and, thereby increasing the exciton dissociation yield.

Based on the results, the group further examined the importance of selecting acceptor comonomers in the Pt‐based Pdots **264**–**267** on their HER [266]. The varied acceptor comonomers resulted in tunable photocatalytic properties of the polymers. The HER was in the order of **267** (7.34 ± 0.82 mmol g^−1^ h^−1^) > **265** (2.26 ± 0.35 mmol g^−1^ h^−1^) > **264** (0.96 ± 0.09 mmol g^−1^ h^−1^) > **266** (0.84 ± 0.05 mmol g^−1^ h^−1^). These values were much higher than that of the pristine Pdots that did not contain the Pt complex. Notably, the higher HER of **267** than **265** was attributed to the lower hydrogen binding free energy of *sp* ^2^ nitrogen in triazole than the sulfur in thiophene. In addition, **267** also showed the longest photocatalytic reaction time, up to 9 h, and produced a total of 31.54 ± 1.39 mmol g^−1^ of H_2_, while **265** showed no obvious photocatalytic activity after 6 h despite its good initial HER, lasting up to 9 h, and produced a total of 31.54 ± 1.39 mmol g^−1^ of H_2_.

Recently, we have rationally designed and synthesized a series of “all‐in‐one” Pt(II) polymers **268**–**273** that integrated the PS, electron transfer mediator, and catalyst in one structure [267]. The introduction of Pt into the polymer skeleton not only enhanced the light absorption but also afforded a highly efficient intramolecular ET process. By introducing different types of donor linkers, the Pt polymers exhibited tunable optical, physical, and photocatalytic properties. Among them, the Se‐containing polymers showed better H_2_ production performance than the Te‐containing polymers. Under visible light irradiation and without the need of a cocatalyst, the HERs of these polymers were 0.22 (for **268**), 0.11 (for **269**), 0.37 (for **270**), 0.16 (for **271**), 1.76 (for **272**) and 0.56 mmol g^−1^ h^−1^ (for **273**), respectively. With optimized catalytic conditions, **272** achieved an HER of 3.09 mmol g^−1^ h^−1^ with an AQY of 0.43% at 430 nm. This work demonstrated efficient material design for photocatalysis.

Poly(arylene ethynylene) (PAE) is extraordinarily attractive owing to its conjugated planar structure, which favors charge separation and transport within the material. In 2019, Huo et al. designed a series of bithiazole‐based Zn polymers **274**–**278** containing PAE and evaluated their PHP activities [268]. Under visible‐light irradiation, the HERs followed the order of **276** (14.32 mmol g^−1^ h^−1^) > **277** (12.56 mmol g^−1^ h^−1^) > **275** (11.63 mmol g^−1^ h^−1^) > **278** (9.97 mmol g^−1^ h^−1^) > **274** (7.18 mmol g^−1^ h^−1^) > PAE (0.05 mmol g^−1^ h^−1^), indicating that the ratio between the ligand and Zn had a significant effect on the HER activity. By increasing the content of Zn, the H_2_ generation activity of the polymer was first enhanced and then decreased.

Yu et al. constructed two bpy‐based Co polymers **279**–**280** by copolymerizing bpy with electron‐donating benzodithiophene block and electron‐accepting perylenediimide unit for PHP [269]. In these functionalized polymers, the conjugated backbone acted as the light‐harvesting antenna, and Co(bpy)Cl_2_ served as the electron‐transfer conduits and catalytic active sites. Upon Co(II) binding, **279** with stronger conjugation skeleton displayed significant redshift of the CT absorption band, while little change was observed in **280** with twisted linkage between the bpy and perylenediimide units. Interestingly, the highest HER (0.28 µmol h^−1^) was achieved for **279** with 10%–20% Co loading, and further increasing the content of Co(II) resulted in significantly decreased HER. **280** presented a different rate dependence on the Co(II) concentration compared to **279**, with an optimal HER of 0.71 µmol h^−1^ at significantly higher Co(II) loadings (maximum at ∼60%). This work demonstrated the influence of the cobalt site density on the PHP activity. Table 3 summarizes the catalytic performance of representative metal polymer‐based photocatalytic systems.

**TABLE 3:** Representative metal polymer‐based photocatalytic systems.

	Photocatalytic properties							Refs.
	PS/catalyst	SED	solvent	Light source	Main product	Rate (Selectivity)	TON	
Ir	**257/**Pt	TEA	DMF/H_2_O	300 W Xe lamp, λ > 420 nm	H_2_	261.2 µmol g^−1^ h^−1^		[260]
	**261**	AA	H_2_O/Pdots	20 W white LED lamp λ > 420 nm	H_2_	444.3 µmol g^−1^ h^−1^		[264]
Pt	**262**	DEA	H_2_O/Pdots	20 W white LED lamp, λ >420 nm	H_2_	12.7 ± 0.6 mmol g^−1^ h^−1^		[265]
	**267**	TEA	H_2_O/Pdots	20 W white LED lamp, λ >420 nm	H_2_	7.34 ± 0.82 mmol g^−1^ h^−1^		[266]
	**272**	TEA	MeOH/H_2_O	300 W Xe lamp, λ > 420 nm	H_2_	3.09 mmol g^−1^ h^−1^		[267]
Zn	**276**	TEOA	lactic acid/H_2_O	300 W Xe lamp, λ > 420 nm	H_2_	14.32 mmol g^−1^ h^−1^		[268]
	**283**	TEA	H_2_O	300 W Xe lamp, λ: 400–750 nm	H_2_	530 µmol g^−1^ h^−1^	23.5 (20 h)	[270]
				sunlight	H_2_	857 µmol g^−1^ h^−1^	11.9 (6 h)	
	**283/**Pt	TEA	H_2_O	300 W Xe lamp, λ: 400–750 nm	H_2_	14727 µmol g^−1^ h^−1^	1176.9 (11 h)	
				sunlight	H_2_	12000 µmol g^−1^ h^−1^	521.8 (6 h)	
	**283**	TEA	CH_3_CN/H_2_O	300 W Xe lamp, λ: 400–750 nm	CO	438 µmol g^−1^ h^−1^ (> 99%)	7.8 (8 h)	
				sunlight	CO	298 µmol g^−1^ h^−1^	3.9 (6 h)	
	**283/**Pt	TEA	CH_3_CN/H_2_O	300 W Xe lamp, λ: 400–750 nm	CH_4_	292 µmol g^−1^ h^−1^ (> 97%)	63.4 (30 h)	
				sunlight	CH_4_	160 µmol g^−1^ h^−1^	6.9 (6 h)	
	**CA‐287**/Pt	AA	H_2_O/Pdots (pH = 4.3)	AM 1.5G, 100 mW cm^−2^	H_2_	534 µmol g^−1^ h^−1^		[271]
Ru	**284**	TEA	CH_3_CN/H_2_O	300 W Xe lamp, λ > 400 nm	CO	3.5 mmol g^−1^ h^−1^ (> 99%)	92.7 (12 h)	[272]
				sunlight	CO	3.4 mmol g^−1^ h^−1^ (> 99%)	44.9 (6 h)	
		BNAH‐TEA	CH_3_CN/H_2_O	300 W Xe lamp, λ > 400 nm	CH_4_	6.7 mmol g^−1^ h^−1^ (> 95%)	208.3 (14 h)	
				sunlight	CH_4_	6.4 mmol g^−1^ h^−1^ (> 95%)	84.7 (6 h)	
Fe	**281/**PVP‐Pt	EDTANa	acetate buffer	100 mW Xe lamp, λ > 400 nm	H_2_	0.87 mmol g^−1^ h^−1^	46.7 (24 h)	[273]
	**SA‐285**	BIH	CH_3_CN/TFE	300 W Xe lamp, 400 ≤ λ ≤ 750 nm	CO	29100 µmol g^−1^ h^−1^ (99.9%)		[274]

#### 3.4.2 Metal Coordination Polymers

Metal coordination polymers, constructed via noncovalent metal–ligand interactions, integrate the directionality and strength of coordination bonds with the structural versatility of polymeric frameworks, thereby facilitating efficient charge transport and enhanced catalytic activity.

**FIGURE 12:**
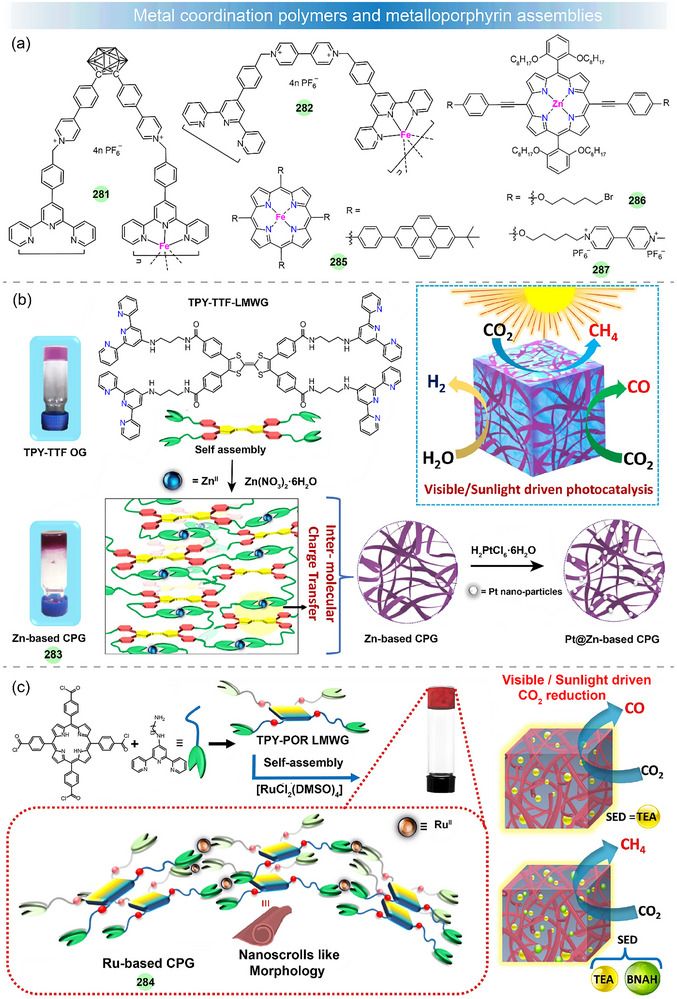
(a) Chemical structures of metal coordination polymers and metalloporphyrin assemblies. (b) The self‐assembly of Zn‐based coordination polymer gel (Zn‐based CPG **283**) and its visible light/sunlight driven photocatalytic activity toward H_2_ evolution and CO_2_ reduction. (c) The self‐assembly of Ru‐based coordination polymer gel (Ru‐based CPG **284**) and its visible light/sunlight‐driven photocatalytic activity toward CO_2_ reduction. (b) Reproduced under the terms of the CC‐BY Creative Commons Attribution 4.0 International license [270]. Copyright 2021, Verma et al., published by Springer Nature. (c) Reproduced with permission [272]. Copyright 2022, Wiley‐VCH.

*o*‐Carborane is an electron‐deficient icosahedral boron cluster that possesses highly polarizable σ‐aromatic character, good chemical and thermal stabilities. Carborane–viologens, combining the advantages of viologens and carborane, show improved electron‐accepting and electron transfer abilities. With the highly electron‐accepting *o*‐carborane viologens (CbV^2+^), He et al. synthesized an iron‐based polymer **281** (Figure 12a) for PHP [273]. Compared with **282** (TON: 24.5, HER: 0.46 mmol g^−1^ h^−1^, AQY: 1.5 × 10^−3^), **281** exhibited enhanced catalytic performance (TON: 46.7, HER: 0.87 mmol g^−1^ h^−1^, AQY: 2.03 × 10^−3^) due to its multiple redox centers, narrow optical bandgap, broad visible light absorption (∼580 nm) and rapid electron transfer. This work proved that introducing CbV^2+^ into the polymer is a feasible strategy for improving the PHP efficiency.

Coordination polymer gels (CPG), formed by the low molecular weight gelator (LMWG) linkers with suitable metals, represent a class of hierarchical soft nanofibrous photocatalytic materials. They allow reactants to easily reach active sites and promote efficient electron transfer between different components. To this end, Maji et al. innovatively designed a tetrathiafulvalene (TTF)‐based LMWG containing a terpyridine (TPY) metal‐binding moiety, and constructed a Zn‐based CPG (**283**) as both PS and photocatalyst for PHP and CO_2_ reduction (Figure 12b) [270]. The integration of electron acceptor unit (TPY) and the electron donation moiety TTF resulted in excellent intermolecular CT, improving the photocatalytic performance. Compared to TPY‐TTF organogel (OG), **283** exhibited similar but enhanced absorption in the visible region, attributed to the increased intermolecular CT from TTF to [Zn(TPY)_2_]^2+^ triggered by the planarization of TPY and facilitated by the increased electron‐accepting tendency of TPY after complexation with Zn^II^ ion in CPG. Using 300 W xenon lamp as the light and TEA as the SED, **283** showed a photocatalytic activity toward H_2_ production (530 µmol g^−1^ h^−1^, TON_H2_ = 23.5, AQY_H2_ of 0.76% at 550 ± 10 nm) and CO_2_ reduction to CO (438 µmol g^−1^ h^−1^, TON_CO_ = 7.8, selectivity > 99%, AQY_CO_ of 0.96%) regulated by the CT interactions. Furthermore, with Pt as a cocatalyst, the HER for **283** increased to 14727 µmol g^−1^ h^−1^ with a TON_H2_ of 1176.9. Interestingly, with the Pt cocatalyst for photocatalytic CO_2_ reduction reaction, CH_4_ was produced instead of CO for **283**, with an activity of 292 µmol g^−1^ h^−1^ and a TON_CH4_ of 63.4, selectivity > 97%, and the highest AQY for the CH_4_ formation being 0.81% at 550 ± 10 nm. Also, upon direct sunlight irradiation at ambient conditions, **283** showed a maximum HER of 857 µmol g^−1^ h^−1^ with a TON_H2_ of 11.9 in 6 h, while a higher activity (12000 µmol g^−1^ h^−1^, TON_H2_ of 521.8) was obtained with a Pt cocatalyst. The highest CO activity was 298 µmol g^−1^ h^−1^ (TON_CO_ = 3.9), and the highest CH_4_ activity with Pt cocatalyst was 160 µmol g^−1^ h^−1^ (TON_CH4_ of 6.9) observed for the sunlight‐driven CO_2_ reduction with **283**. The CPG materials provide a promising platform for solar‐energy‐driven fuel production.

In the following year, the same authors developed Ru‐based CPG **284** by self‐assembling a tetrapodal terpyridyl porphyrin‐based LMWG (**TPY‐POR**) with RuCl_2_ for photocatalytic CO_2_ reduction (Figure 12c) [272]. **284** exhibited a new shoulder absorption peak at 476 nm, corresponding to the MLCT band from Ru^II^ (*dπ*) to terpyridine (π*) transitions, and enhanced visible light absorption compared to **TPY‐POR** OG. Interestingly, after Ru^II^ coordination with **TPY‐POR** LMWG, the morphology of OG changed from sheets to nanoscrolls. Under visible light irradiation, **284** produced CO with > 99% selectivity (3.5 mmol g^−1^ h^−1^, TON of 92.7, AQY of 3.7%) with TEA as SED. By utilizing a combination of 1‐benzyl‐1,4‐dihydronicotinamide (BNAH) and TEA as SED, **284** achieved an eight‐electron reduction of CO_2_ to CH_4_ with over 95% selectivity compared to CO (6.7 mmol g^−1^ h^−1^ with a TON_CH4_ of 208.3, AQY of 7.67%) under visible light. This was because the TEA helped reduce BNA^+^ to BNAH or BNA_2_s, thereby enhancing the photocatalytic activity of CH_4_ production. In the sunlight‐driven photocatalytic CO_2_ reduction, **284** produced CO with the highest TON of 44.9 (rate: 3.4 mmol g^−1^ h^−1^ and selectivity *>* 99%) in the presence of TEA, and catalyzed CO_2_ reduction to CH_4_ with the highest TON of 84.7 (rate: 6.4 mmol g^−1^ h^−1^ and selectivity: *>* 95%) in the presence of BNAH and TEA. Thus, the efficient photocatalytic activity of **284** highlights the practical applicability of this soft hybrid material for solar‐energy‐driven fuel production.

### 3.5 Metalloporphyrin Assemblies

Metalloporphyrins, possessing broad absorption of visible light in the range of 400–600 nm, a more positive LUMO energy level, and long‐lived triplet exciton, are excellent candidates as PSs or direct catalysts for solar‐to‐fuel conversion. Benefiting from their rigid and planar geometry, metalloporphyrin monomers easily self‐assemble into supramolecular structures by noncovalent interactions, including hydrogen bonds, π–π stacking, electrostatic interactions, coordination interactions, and hydrophobic interactions [275, 276, 277]. Compared with individual metalloporphyrin molecules, the self‐assembled nanostructure can exhibit broadened and redshifted absorption, varied luminescent properties, enhanced photoelectronic response, and improved photostability. Meanwhile, the photocatalytic activity of the metalloporphyrin is also highly dependent on its morphology, size, and orderly stacking [278, 279]. Up to date, various strategies have been developed to modify the self‐assembly behaviors and catalytic activities of metalloporphyrins, such as chemical structural modification, the use of surfactants with different electronegativity, wet chemical reaction, tuning of pH, addition of chloride ions, and so on [280, 281]. Recently, we have summarized the progress of self‐assembled (SA) metalloporphyrins in PHP [282]. In this part, we will mainly focus on the latest developments on the photocatalytic performance of assembled metalloporphyrins to supplement the recent review [282].

In 2023, Kosugi et al. reported a SA Fe(III)‐porphyrin **(SA‐285)** with pyrene moieties for CO_2_ reduction with high activity (Figure 12a) [274]. In terms of the molecular design, Fe(III)‐porphyrin functionalized as the catalytic site for CO_2_ reduction, and the pyrene moieties served as light‐harvesting units. The supramolecular catalyst (**SA‐285**) was achieved through self‐assembly of monomers via noncovalent interaction sites using a simple recrystallization technique. **SA‐285** showed excellent light‐harvesting ability in the visible light region. Photocatalytic CO_2_ reduction was carried out under visible light (400 ≤ λ ≤ 750 nm) in a CO_2_‐saturated acetonitrile solution containing a suspension of the photocatalyst **SA‐285**, trifluoroethanol as a proton source, and BIH as SED. A CO production rate of 29 100 µmol g^−1^ h^−1^ (TON_CO_ of 1220, 24 h, 99.9% selectivity) and a TOF of 50.8 h^−1^ were achieved for **SA‐285**.

In addition, Cai et al. directly utilized a hydrophilic polymer (PEG‐PS‐COOH) to encapsulate Zn‐porphyrin, forming co‐assemblies (**CA‐287**) that improved the stability and aqueous solubility of metalloporphyrin [271]. In the molecular design, Zn‐porphyrin was covalently linked with positively charged viologen to form a distinct D–A structure, promoting the photoinduced charge separation (CS). The assembled nanoparticles (**CA‐287**) in aqueous solution not only exhibited rapid charge separation but also displayed a long‐lived CS state with a lifetime of 4.3 ms. This surprisingly long‐lived lifetime could be attributed to the charge hopping within the nanoparticles, which was enabled by the close packing of molecules. Additionally, the increased distance between the reduced viologen and Zn‐porphyrin, caused by Coulomb repulsion, slowed down charge recombination. Based on this long‐lived CS state, **CA‐287** nanoparticles exhibited an optimal HER of 534 µmol g^−1^ h^−1^ with 6 wt% Pt as the cocatalyst and AA as the SED, which was 2‐fold higher than that of **CA‐286** nanoparticles, indicating that the tunable CS state lifetime of molecular aggregates controlled by nanoparticles greatly contributed to advancements in solar energy conversion applications.

Based on the above discussions, we have concluded that the comprehensive exploration of reliable strategies for designing and fabricating metal complex‐based PSs in photocatalysis has important significance for the intensive investigation of their fascinating properties and improving photocatalytic performances. First, rational ligand engineering can modulate the absorption, excited‐state lifetimes, and redox potentials of metal complexes, such as incorporation of electron‐donating/withdrawing groups, functional chromophores, and extending the π‐conjugation of the ligand. Second, the coordination geometry and stacking patterns (such as orthogonal, head‐to‐tail) of metal complexes facilitate long‐lived charge‐separated states and efficient electron transfer, which is beneficial for improving photocatalytic efficiency. Additionally, strong *σ*‐donors (such as N‐heterocyclic carbene ligands) stabilize the MLCT states, prolonging the excited lifetimes and improving the photocatalytic activity. Third, the multicomponent systems integrating photosensitizer and catalyst units within a single structure (such as dyads and metallacages) facilitate efficient intramolecular energy/electron transfer and synergistic catalytic effect. The oligomeric/metallopolymer structures enhance stability and steric hindrance, and facilitate charge separation and migration. Lastly, hydrophilic modifications (such as PEGylation) and encapsulation strategies enable reactions to occur in aqueous media and improve photostability, which will promote interfacial reactions and be beneficial for the enhancement of photocatalytic efficiency.

Therefore, the structure of metal complex‐based PSs—especially ligand type, substituent position, coordination geometry, stacking pattern, and integration with other functional units—directly determines their photophysical/electrochemical properties and photocatalytic activity. Rational design and modification of these features enable control over light absorption, excited‐state lifetimes, electron transfer rates, stability, and ultimately, the efficiency and selectivity of solar‐driven chemical energy conversion such as H_2_ evolution, CO_2_ reduction, and H_2_O_2_ production.

In summary, in this section, we have summarized the recent advances of metal complex‐based PSs for photocatalytic solar‐to‐chemical energy conversion (e.g., H_2_ evolution, CO_2_ reduction, and H_2_O_2_ production), covering catalytic mechanisms, design strategies, and structure–activity relationships. This field has advanced from noble‐metal complexes to earth‐abundant metal complexes, metallopolymers, and supramolecular assemblies. A key feature of photocatalysis is light absorption by PSs, which generates a long‐lived electronic excited state via ISC to facilitate charge separation and electron transfer for driving the catalytic reactions. Thus, broad and strong absorption across the solar spectrum—especially the visible light region—is critical for efficient solar energy utilization. Additionally, ligand modification and metal center selection enable precise tuning of photophysical/electrochemical properties (absorption spectrum, excited‐state lifetime, redox potential) to match diverse catalytic reactions. Noble‐metal PSs leverage heavy‐atom‐induced spin‐orbit coupling (SOC) for long‐lived triplet states and high product selectivity but suffer from high cost. In contrast, non‐noble‐metal PSs achieve comparable performance via ligand field optimization or aggregation control. While noble‐metal PSs remain efficiency benchmarks, future development lies in sustainable, robust, and scalable systems based on abundant elements and advanced materials engineering. Bi‐metal/multi‐metal and metallopolymer PSs integrate light harvesting, charge transfer, and catalysis into one system, simplifying reaction processes and boosting efficiency. Designing PSs and systems that operate efficiently in water is essential for practical solar fuel production and aligns with green chemistry principles.

Metal complex PSs have demonstrated remarkable versatility and efficiency in photocatalytic solar‐to‐chemical energy conversion. However, their practical application is hindered by noble‐metal dependence, solubility limitation, and scalability challenge.

## 4 Metal Complexes for Solar–to–Thermal Energy Conversion

The photothermal effect pertains to the inherent capability of materials to convert light energy into heat upon exposure to sunlight or laser irradiation, enabling a diverse array of applications such as water evaporation, seawater desalination, cancer therapy, pollutant remediation, biosensing, imaging, and more [283, 284, 285, 286, 287]. Photothermal materials occupy a pivotal role in these endeavors, encompassing both single‐component organic and inorganic materials as well as multi‐component composites [288, 289]. Small‐molecule materials and conjugated polymers have garnered significant attention as effective organic photothermal materials, with representative examples including CR‐TPE‐T [290], polypyrrole [291], and others [292, 293, 294]. Inorganic materials, including metal nanoparticles (Au, Ag, Pt, Cu) [295, 296, 297], semiconductors (TiO_2_, MoS_2_, WO_3_) [298, 299], and carbon‐based materials (carbon nanotubes, graphene, carbon black) [300, 301], are also highly efficient in this regard. Recently, 2D framework materials like metal‐organic frameworks (MOFs) and covalent‐organic frameworks (COFs) have demonstrated as promising candidates for photothermal applications [302, 303]. Among these, our interest primarily rests on photothermal metal complexes, which showcase their superiority in the photo‐to‐thermal conversion process. Specifically, metal complexes refer to those in which at least one metal atom is chemically connected with organic compounds through coordinated bonds, thereby enabling the leveraging of synergistic benefits from both metal and organic components. This unique combination imparts exceptional properties and immense potential for diverse applications. Herein, we mainly summarize the recent advancements in molecular structures of photothermal metal complexes and their advancing applications in solar evaporation and seawater desalination, offering valuable guidelines for the future development of solar–to–thermal conversion research.

**FIGURE 13:**
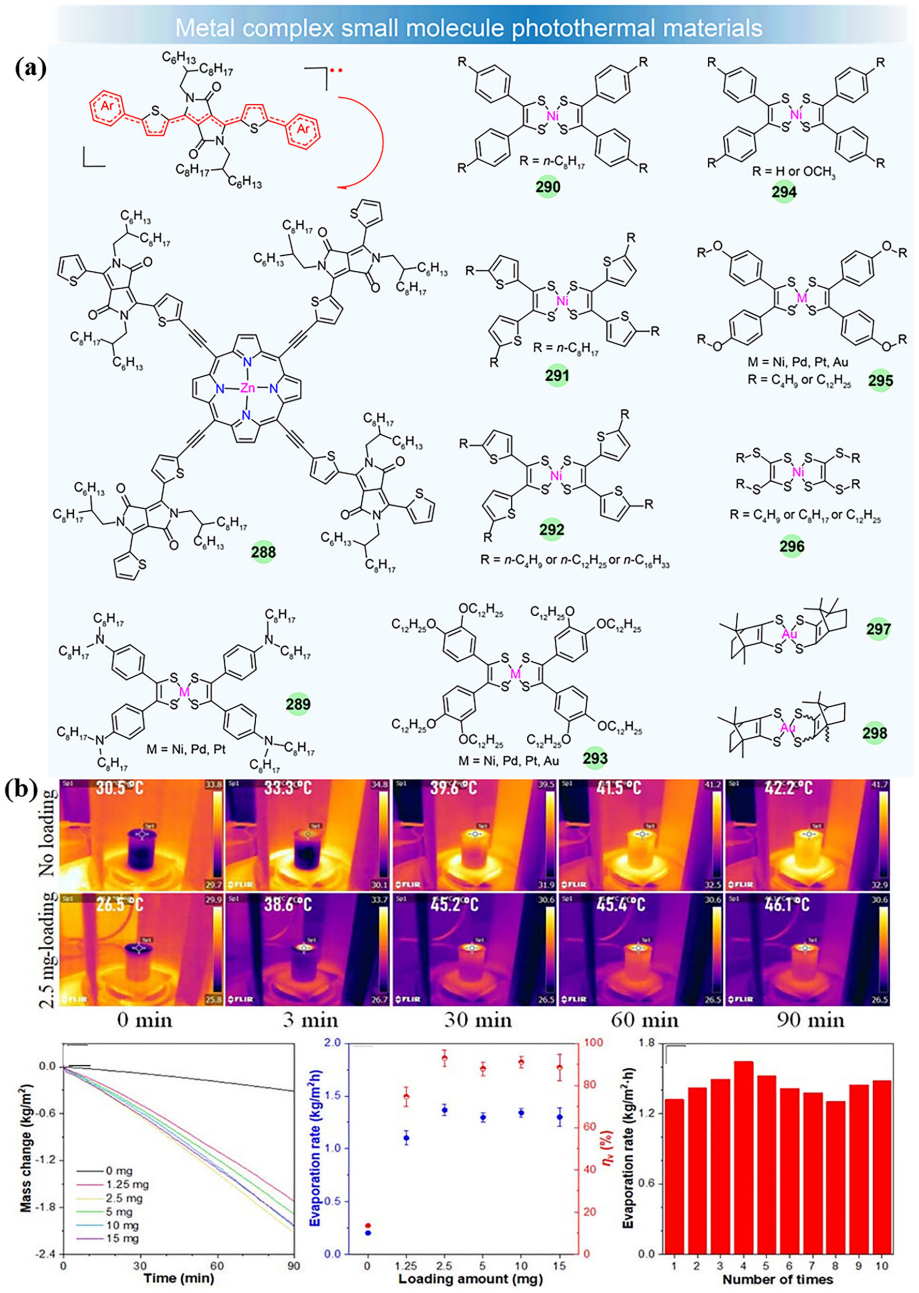
(a) Chemical structures of metallated small molecules as photothermal materials **288**–**298**. (b) Photothermal characterizations and device performance of **289** under one sun irradiation: infrared images of the evaporation layers; water‐mass change curves; the evaporation rate and the corresponding evaporation efficiency of solar evaporator with different loading amounts of **289**; the device stability test. (b) Reproduced with permission [314]. Copyright 2023, Royal Society of Chemistry.

As we all know, highly efficient photothermal materials heavily depend on their sunlight absorption capability, particularly in the near‐infrared region. Developing full‐solar‐spectrum‐absorption molecules can significantly enhance photothermal conversion efficiency. Organic materials offer several advantages, including flexible structural modification, good reproducibility, and adjustable physical and chemical properties, which make them ideal for achieving strong absorption [304, 305, 306]. Common strategies for molecular design involve enhancing intermolecular packing, promoting ICT, and increasing the degree of molecular conjugation to achieve broader and high absorption [307, 308, 309]. Among the various organic materials, metallated materials have garnered considerable attention due to their unique charge transfer mechanisms, such as MLCT, LLCT, ILCT and ligand‐to‐metal charge transfer (LMCT) [310, 311, 312]. These charge transfer pathways present a novel approach for obtaining materials with strong absorption across the entire wavelength range. In 2021, Peng et al. reported a Zn‐porphyrin based small molecule **288** (Figure 13a) with high extinction coefficients and broader absorption wavelength range up to 1500 nm for powder sample [313]. It shows an open‐shell quinoidal diradical structure in aggregated state due to aggregation‐induced radical, leading to strong nonradiative decay and thus highly efficient photothermal conversion properties. The molecule exhibits an obvious electron spin resonance signal, further supporting the mechanism of aggregation‐induced radicals. It was utilized as a photothermal material to fabricate interfacial solar steam generation system. As a result, the highest value of photothermal conversion efficiency and solar‐energy‐to‐vapor efficiency reach 73.2% and 90.6%, respectively, under irradiation of 808 nm laser. Additionally, **288** displays good desalination performance of real seawater. The concentration of four primary ions (K^+^, Ca^2+^, Na^+^, Mg^2+^) reduced significantly after desalination from 2 × 10^2^ mg L^−1^ to 0.76 µg L^−1^, from 4 × 10^2^ mg L^−1^ to 2.07 µg L^−1^, from 8 × 10^3^ mg L^−1^ to 8.6 µg L^−1^, from 1 × 10^3^ mg L^−1^ to 1.25 µg L^−1^, respectively. Each of the desalinated ion concentrations is below 1 mg L^−1^, which is in excellent accordance with the drinking water standard set by the World Health Organization (WHO). This indicates that the evaporation system is indeed viable for seawater desalination and the production of clean water.

In 2023, Ni et al. developed three neutral *d* ^8^ transition‐metal (nickel, palladium, and platinum) complexes **289** [314]. They exhibit excellent sunlight harvesting capability with UV‐vis‐NIR absorption region spanning from 300 to 1700 nm, which results from both intervalence charge‐transfer transition and strong intermolecular interactions induced by the square‐planar geometries. These complexes were deposited on the surface of filter papers to evaluate their photothermal properties. Under irradiation of 1064 nm laser and the corresponding 1 W cm^−2^ power for 16 s, the temperature of filter papers with complexes can sharply rise to 185.5°C (Ni center), 154.8°C (Pd center), and 166.5°C (Pt center), respectively, which is much higher than 33.8°C of blank filter‐paper under the same condition. Therefore, nickel complex delivers the best photothermal properties, and it was used to fabricate solar‐energy‐to‐vapor interfacial layer to evaluate the device performance. As depicted in Figure 13b, after solar irradiation over 3 min under one sun irradiation, the equilibrium temperature of evaporator surfaces arrives to 38.6°C for the device at 2.5 mg of Ni complex loading, which is higher than that of 33.3°C for the control device without loading. The deionized water evaporation rate for the corresponding solar evaporator can reach as high as 1.37 ± 0.05 kg m^−2^ h^−1^ for the corresponding solar evaporator. After device optimization, the best water mass change and solar‐energy‐to‐vapor conversion efficiency can be achieved at 1.406 kg m^−2^ h^−1^ and 95.64%, respectively, for 2.5 mg Ni‐based solar evaporator system. Moreover, the device also exhibits excellent performance in seawater desalination, and the freshwater obtained meets the standards set by WHO. In 2025, they further synthesized two organonickel bis(dithiolene) complexes: compound **290**, substituted with phenyl groups and compound **291**, substituted with thienyl groups. Then they conducted a systematic comparison of their photothermal properties [315]. Both molecules present intense NIR‐II (1000–1700 nm) absorption, attributed to their delocalized ligand's intervalence charge transfer (IVCT) transitions. DFT calculations indicate that introducing electron‐donating thienyl rings in **291** can reduce the dihedral angle of the whole molecule, as well as the transition and adiabatic energies between the ground and excited states. Consequently, compared to **290** with an NIR‐II absorption peak at 1022.2 nm, **291** exhibits a bathochromic shift of the absorption peak to 1083.6, and demonstrates greater nonradiative decay due to a the higher internal conversion rate. It is well known that nonradiative decay in these complexes is a key mechanism for photothermal energy generation. After irradiation with 1064 nm of laser (1 W/cm^2^) for 15 min, the temperatures of their aggregates in deionized water are up to 48.9°C for **291** and 38.7°C for **290**, corresponding to photothermal conversion efficiencies of 48.6% and 29.2%, respectively. The complexes were further deposited onto the surface of melamine foam to fabricate solar steam generation systems. Owing to its superior photothermal property, the **291**@foam evaporator shows a higher water evaporation rate of 1.99 ± 0.10 kg m^−2^ h^−1^ and a solar‐energy‐to‐vapor efficiency of 135.6 ± 6.63% under one‐sun irradiation, compared to **290**@foam evaporator with a water evaporation rate of 1.83 ± 0.06 kg m^−2^ h^−1^ and an efficiency of 112.0 ± 3.95%. The 3D structure of the evaporators is likely a major factor leading to efficiencies exceeding the theoretical value of 100%. Recently, they reported another study on the photothermal performance of organonickel complexes, specifically compounds **292** [316]. This work investigated how alkyl chain engineering in organonickel bis(dithiolene) complexes affects their photothermal properties and evaporation performance. Extending the alkyl chain length significantly enhances thermal stability, absorption intensity, photothermal temperature and the salt rejection capability of the evaporators owing to the inherent hydrophobicity of the alkyl chains. Under one‐sun radiation, **292** with *n*‐hexadecyl substituents exhibits the highest photothermal conversion efficiency of 30.1%, evaporation rates of 1.72 ± 0.09 and 1.67 ± 0.06 kg m^−2^ h^−1^ for deionized water and seawater, respectively. The evaporator also demonstrates excellent durability and seawater desalination performance. Ni's research provides valuable insights and guidance for the molecular design of highly efficient organometallic photothermal materials and their applications.

In 2024, Camerel's team also conducted a photothermal study using metal complexes with molecular structures similar to those reported by Ni's group. They characterized a chemical library of nineteen metal‐bis(dithiolene) complexes **293**–**298** to investigate their photophysical and photothermal properties [317]. They can absorb the low‐energy photons in the near‐infrared region from 650 to 950 nm, including all the nickel, palladium and platinum complexes. Specifically, the gold complexes exhibit strong absorption in the longer wavelength range from 1500 to 1700 nm, thereby demonstrating themselves to be among the most efficient photothermal molecules. They further investigate the photothermal index *I*_PT_ defined by themselves, which combines both molar absorptivity *ε* and photothermal efficiency *η*, to assess the photothermal activity of all these molecules. The majority of nickel complexes show lower photothermal activity compared with palladium and platinum complexes. Only nickel complexes featuring diphenylethylenedithiolate ligands with long carbon chains exhibit comparable photothermal activity. Gold complexes are not the best photothermal agents among all molecules, even though they exhibit much higher infrared absorption. Additionally, it is evident that all dithiolene complexes outperform the perylene derivative as photothermal agents. The linear correlation appears more pronounced when considering a single metal center. These studies indicate the significant potential of metallated materials in photothermal applications, and the selection of the metal center can have a substantial impact on their absorption window, thereby influencing the photothermal conversion efficiency.

**FIGURE 14:**
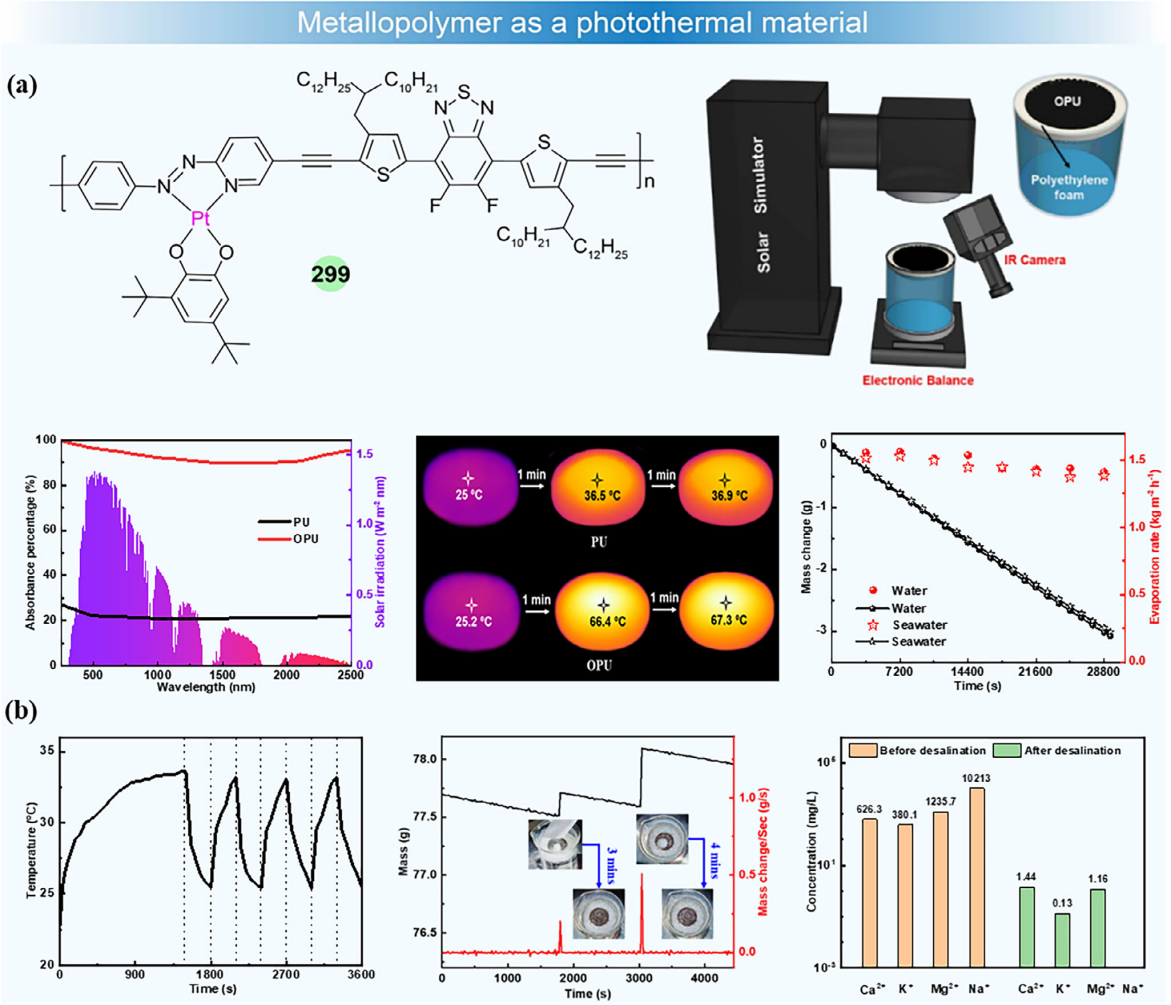
(a) Chemical structure of metallated photothermal polymer **299**; photothermal equipment schematic diagram; absorption spectra of the dry evaporation layers; infrared images of the dry evaporation layers under one sun irradiation; the water‐mass change curve; evaporation rate of evaporator for water and seawater under one sun irradiation. (b) Light on‐off cycle test; salt rejection performance and ICP‐OES results of the evaporator. (b) Reproduced with permission [318]. Copyright 2024, Royal Society of Chemistry.

Recently, Wong et al. developed a novel metallopolymer polymer **299** through Sonogashira coupling reaction and then fabricated an interfacial solar steam generation system (OPU) by depositing **299** particles onto a polyurethane foam (PU) substrate, as depicted in Figure 14a [318]. From the perspective of material design, the **299** incorporates the platinum coordination structure and D–A building block into one polymer to enhance the infrared photon capture properties for a higher photothermal conversion effect. The **299** particles were obtained through several cycles of ultrasonication treatment, with a size of about 256.6 nm and a polydispersity index (PDI) of 0.261. The dry evaporation layer incorporating the polymer shows a markedly enhanced absorption intensity across nearly the entire solar spectrum compared to the blank evaporation layer. The temperature variation on the **299**‐attached PU surface, referred to as OPU, is measured under 1 sun irradiation to investigate photon‐to‐heat conversion behavior. The highest value of 66.4°C can be observed after 1‐min irradiation, significantly exceeding the 36.5°C of the blank PU substrate. This indicates that **299** exhibits good photon‐to‐thermal conversion ability, which is attributed to its full‐solar‐spectrum‐absorption as well as the efficient energy release through non‐radiative relaxation mechanism. DFT calculations suggest that the excellent sunlight absorption capability of **299** results from the synergistic effects of intermolecular and ligand‐to‐ligand charge transfer mechanisms, which is an innovative finding in this research. Consequently, the OPU generated maximum evaporation rates of 1.57 kg m^−2^ h^−1^ and 1.52 kg m^−2^ h^−1^ for water and seawater samples under 1 sun, respectively, with the corresponding evaporation efficiencies of 85.6% and 83.1%. As solar irradiation intensity increased, the evaporation rate of OPU also grew, while its evaporation efficiency declined. The photothermal response of the OPU under one sun irradiation was further evaluated by several light on–off cycle tests during water evaporation, where the light‐on/off test was repeated at 5‐min intervals (Figure 14b). Initially, the top‐surface temperature of the wet OPU rose from 22.8°C and stabilized at 33.6°C after 25 min of continuous irradiation. Subsequently, the light was switched off for 5 min, causing the top‐surface temperature to gradually decline. When the light was on, the top‐surface temperature of the OPU exceeded 33°C within 5 min and did not fall below 25.4°C when the light was turned off for 5 min. The results indicated that the platinum‐based polymer exhibits stable photo‐to‐thermal conversion capabilities. Furthermore, the OPU displays good salt rejection ability and seawater desalination performance. 500 mg of NaCl crystal could be dissolved back into bulk seawater within 4 min as placed on the top surface of the evaporator. The concentrations of the four cations, Ca^2+^, K^+^, Mg^2+^, and Na^+^ cations were obviously reduced by three orders of magnitude after desalination. Notably, the OPU showed excellent salt rejection capability, as 500 mg of NaCl crystal could be dissolved back into bulk seawater within 4 min as placed on the top surface of the evaporator. This work introduces an innovative molecular design strategy to develop highly efficient photothermal materials by integrating a suitable metal complex with electron‐withdrawing groups into one polymer backbone, constructing multiple charge transfer mechanisms. **299** is also the first reported 1D metallated photothermal polymer material. This type of polymers is expected to reduce cost and enhance the performance of water evaporation and seawater desalination through continuous molecular modification in the future.

**FIGURE 15:**
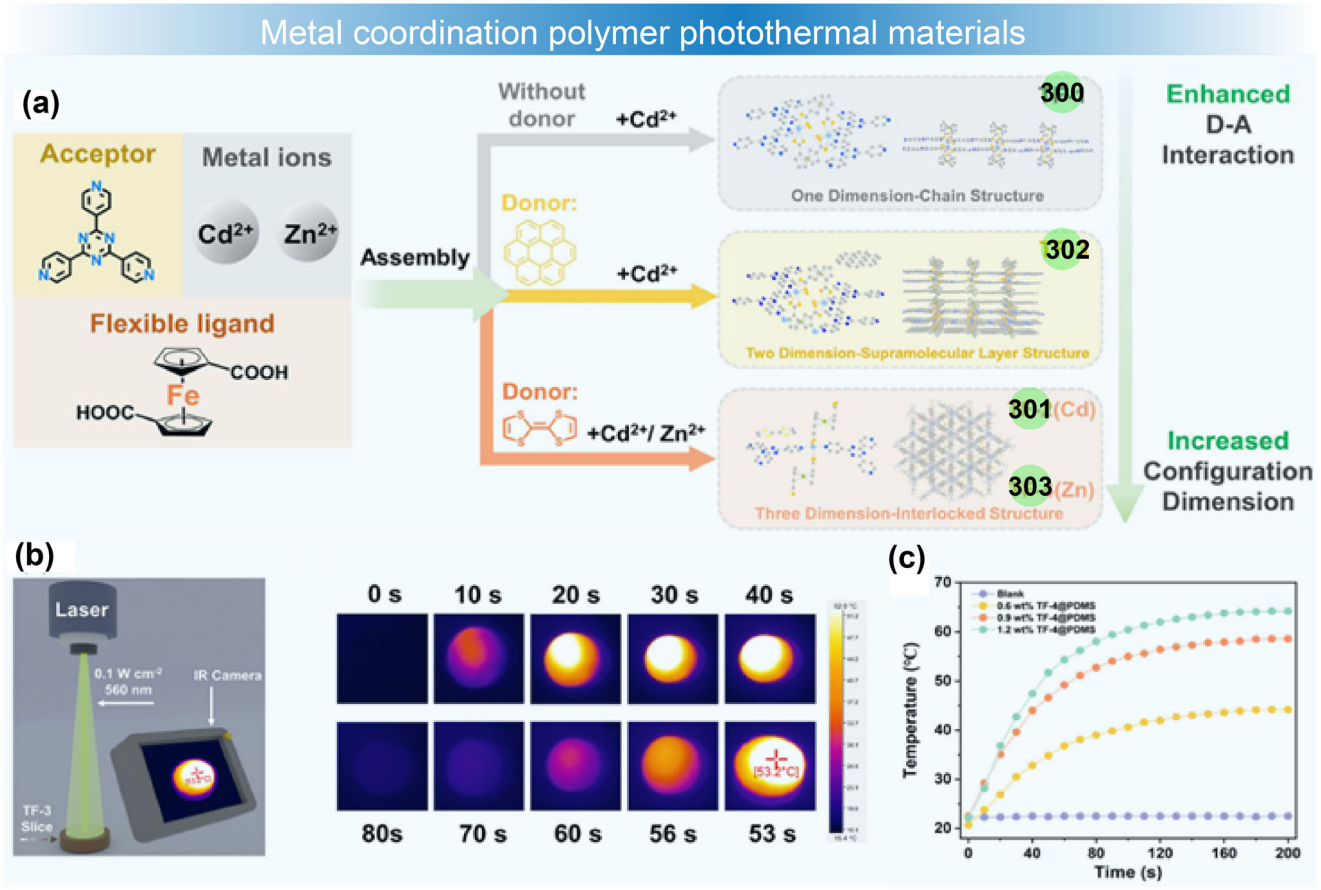
(a) Chemical structures and designed principles of the four coordination polymers, **300**–**303**. (b) Schematic diagram of the measurement system of photothermal conversion effect and infrared thermal images of **302** under laser irradiation at 560 nm with a power of 0.1 W cm^‒2^. (c) Temperature‐time curves of **303@PDMS** patches with varying weight loadings under laser irradiation at 808 nm with a power of 0.5 W cm^‒2^. (a–c) Reproduced with permission [319]. Copyright 2024, Science China Press.

Coordination polymers (CPs) featuring D–A structures have emerged as a promising class of photothermal polymers with diverse potential applications, including photothermal therapy (PTT) and highly efficient solar–to–thermal conversion. In 2024, Chang et al. constructed a new series of CPs based on the flexible linker 1,1′‐ferrocenedicarboxylic acid (FCA) and the incorporation of D–A interactions into the polymer design [319]. By carefully regulating metal ions and donor groups, four distinct CPs were successfully obtained, namely {[Cd(FCA)(TPT)H_2_O]·DMF}_n_ (**300**), {[Cd_3_(TPT)_2_(FCA)_3_ (H_2_O)_3_·TTF]}_n_ (**301**), {[Cd(FCA)(TPT)H_2_O]·coronene}_n_ (**302**), and {[Zn_3_(TPT)_2_‐(FCA)_3_(H_2_O)_3_·TTF]}_n_ (**303**), respectively. These CPs exhibit enhanced D–A interactions and increased configuration dimensions, as shown in Figure 15a. Compounds **300** and **302** show 1D structures, whereas **301** and **303** display 3D structures. This indicates that the advantages of structural diversity and highly tunable properties of these CPs. Compared with **300** without a donor group, it was found that **302** with the similar configuration shows a higher photothermal conversion behaviour after the introduction of a coronene donor group. As depicted in Figure 15b, under laser irradiation at 560 nm with power below 0.1 W cm^−2^, the temperature of **302** increases from room temperature to 53.2°C within 53 s. In contrast, under the same irradiation conditions, the temperature of **300** only reaches 48°C. This distinctive photothermal effect can be attributed to the stronger D–A interactions of **302**. Compared to the linear structure of **300** and **302**, the 3D structure of **301** shows a significantly higher absorption intensity in the longer wavelength range within 550–800 nm. This leads to enhanced photothermal pefermance of **301** under laser irradiation at 808 nm at a power of 0.1 W cm^−2^. The temperature values of **300**, **301** and **302** rise to 26°C, 33°C and 29°C, respectively, within 57 s. To reduce the toxicity risk associated with the heavy metal in **301**, the Zn(II) ion was selected to replace the Cd(II) ion, resulting in a new CP of **303** for the potential biological applications in PTT. Similar to compound **301**, **303** presents a sensitive response to a laser wavelength at 808 nm and the temperature promptly reaches nearly 34.5°C with a laser power of 0.1 W cm^‒2^. Several biological nonpoisonous patches **(303@PDMS**) were developed through blending transparent PDMS gel with different weight ratios of **303** and the phothermal performance of these patches was then investigated. As shown in Figure 15c, under the laser irradiation at 808 nm with a power of 0.5 W cm^‒2^ for nearly 120 s, the temperatures of the **303@PDMS** patches increased by 20°C, 34°C and 42°C for doping ratios of 0.6 wt%, 0.9 wt% and 1.2 wt%, respectively. The maximum temperature reached 61°C with an increased power density of 0.9 W cm^‒2^, suggesting that **303** has a decent infrared‐to‐heat conversion response. This research developed a new library of D–A CPs as effective photothermal candidate materials. Given the excellent photothermal conversion effect of the CP/PDMS patches, these materials show great potential for applications in flexible wearable medical devices in the future. All the photothermal properties and performance of the metallated materials discussed in this study are summarized and presented in Table 4.

**TABLE 4:** Summary of the photothermal performance of metal complex materials. (*λ*_max_, *λ*_laser_, P_laser_, T_max_, *η_thermal_* and *η_vapor_* represent the absorption peak wavelength, laser wavelength, laser power, the maximum temperature under laser irradiation, photothermal conversion efficiency and solar‐to‐vapor efficiency in water, respectively).

Material	*λ*_max_ (nm)	*λ*_laser_ (nm)	P_laser_ (W/cm^2^)	T_max_ (°C)	*η_thermal_* (%)	*η_vapor_* (%)	Refs.
**288**	589, 776	808	1	140	73.2	90.6	[313]
**289**	1054 (Ni)1067 (Pd)984 (Pt)	1064	1	41.234.538.7	42.3	95.6(Ni)	[314]
					17.1		
					18.4		
**290**	1083.6	1064	1	48.9	48.6	135.6	[315]
**291**	1022.2	1064	1	38.7	29.2	112.0	
**292**	1040(NiTh16)	1064	1	64.7 (NiTh16)	30.1 (NiTh16)	98.48	[316]
**293**	961 (Ni)983 (Pd)897 (Pt)1614 (Au)	9409408801600	3	91.189.2102.669.8	48.056.439.341.1	—	[317]
**294**	929 (Ni)950 (Pd)866 (Pt)1568 (Au)	9409408801600	3	88.194.693.379.8	45.544.432.339.4	—	
**295**	858 (H)924 (OCH_3_)	880940	3	67.075.2	30.639.5	—	
**296**	1067 (C_4_H_9_)984 (C_8_H_17_)984 (C_12_H_25_)	940	3	69.371.470.1	56.564.257.3	—	
**297**	1500	1600	3	67.5	66.4	—	
**298**	1500	1600	3	67.6	63.5	—	
**299**	400–1400	—	—	67.3	—	86.6	[318]
**300**	500	560	0.1	48	—	—	[319]
**301**	400, 750			43	—	—	
**302**	500			53.2	—	—	
**303**	500, 750	560	0.1	35.1	—	—	
**303@PDMS**		808	0.9	61	—	—	

For photothermal applications of metal complexes, the main basis for molecular design is to enhance their sunlight absorption across the entire solar spectrum. Near‐infrared absorption beyond 780 nm is particularly important for achieving high photothermal conversion efficiency, as photogenerated excitons in this region predominantly undergo nonradiative decay to produce heat energy. The released heat energy accumulates within the material and must be transferred to the material's surface or the surrounding medium through thermal conduction, convection, or radiation before it can be effectively utilized. To enhance the heat transfer process, photothermal metal complexes should exhibit good solubility or dispersion in commonly used organic solvents, such as methanol, isopropanol, chlorobenzene, or chloroform, enabling the formation of uniform nanoparticles or particles on the scale of hundreds of nanometers. Increasing molecular interactions, such as π‐π stacking, can further enhance both nonradiative decay and heat transfer efficiency. For photothermal metal complexes, several molecular design principles have been proposed based on recent studies. Introducing one or more electron‐withdrawing groups in conjunction with an electron‐donating metal group is an effective strategy for creating small‐molecule or polymeric metal complexes with extended absorption into the near‐infrared region and enhanced photothermal properties. Incorporating long alkyl chains into metal complexes can improve the hydrophobicity and thermal stability, which is beneficial for seawater desalination performance. Unlike other photothermal materials, metal complexes uniquely utilize multiple charge transfer processes between the metal center and organic ligands, enabling near‐infrared absorption often exceeding 1000 nm even in small molecules. Organonickel bis(dithiolene) complexes and organoplatinum phenylazopyridine complexes have been demonstrated to be successful examples that employ LLCT mechanisms for efficient photothermal conversion. Developing additional structures with these features offers a great potential for metal complexes to compete with other photothermal materials in future commercial applications.

In this section, we primarily summarize the molecular structures, photothermal properties, and solar evaporation performance of currently reported metal complexes. Effective molecular design principles are presented to elucidate the structure–activity relationships and guide further advancements in metallated photothermal materials. Metallated porphyrin moieties, as well as nickel, gold, palladium, and platinum complexes, have been demonstrated as promising photothermal candidates. The choice of ligand structure is a decisive factor in achieving strong near‐infrared absorption and, consequently, high photothermal conversion efficiency in metal complexes. Additionally, side‐chain modifications can further enhance their photothermal properties. Notably, small‐molecule metal complexes exhibit superior sunlight absorption beyond 800 nm—a feature rarely achieved by other small molecules without metal incorporation. This advantage is attributed to the multiple interactions and rapid charge transfer between the metal center and organic ligands.

Conjugated polymers or coordination polymers, formed by combining metal complexes with electron‐withdrawing groups, show great potential for achieving a full solar absorption spectrum. In these polymers, intermolecular interactions, the degree of molecular conjugation, and material stability are significantly enhanced, resulting in a broad absorption window and excellent photothermal conversion efficiency. However, a comprehensive understanding and precise regulation of the complex charge transfer and nonradiative transition processes in these metal complexes remain elusive. The development of complexes based on inexpensive metals is necessary to further reduce material cost, potentially making them even more economical than current carbon‐based materials such as graphene. Scalable production technologies for metal complexes beyond laboratory conditions should be explored, focusing on simple synthetic routes and high product yields. Finally, the integration of metal complexes into photothermal devices remains a major challenge that must be addressed for the future commercialization of photothermal metal complexes.

## 5 Conclusions and Prospects

This review highlights the significant potential of tunable metal complexes in solar‐light‐driven energy conversion fields which comprise photovoltaics (such as OSCs, PSCs, DSSCs), photocatalysis (such as H_2_ production, CO_2_ reduction and H_2_O_2_ production) and photothermal (such as water treatment and seawater desalination) conversion applications. Structural modification of metal complexes can effectively regulate their optoelectronic properties, including energy levels, absorption, emission, thermal stability, charge mobility, exciton lifetime and charge transfer mechanisms. These tunable features make metal complexes promising candidates for emerging photovoltaic applications. Metal complexes with broad and intense absorption in the visible light region, long excited‐state lifetime, rich redox properties and high photochemical stability are particularly attractive for photocatalysis. They have demonstrated excellent activity and selectivity in various photocatalytic systems, including single‐component bifunctional systems and two‐component systems. Additionally, metal complexes with absorption edges extending beyond 1500 nm show significant potential as highly efficient photothermal materials. The ligand structures and metal centers can significantly influence their photo‐to‐thermal conversion efficiency. This review provides the first comprehensive introduction and summary of photothermal metal complexes, representing a significant material innovation in the ongoing advancement of photothermal materials.

### 5.1 Functional Metal Complexes for Photovoltaics

Metal complexes have already shown great potential in photovoltaic energy conversion, and ongoing exploration and research by scientists are essential. First, the inherent nature of metal coordination bonds leads to a diverse range of structures in metal complexes, which adds a degree of uncertainty. Second, metals like Pt and Ir are precious, making the material cost uncontrollable. Third, most metals have poor compatibility with organic materials, making it difficult to form appropriate mixed morphologies. To address these challenges, several strategies can be adopted: for organic photovoltaics, introducing a trace amount of Pt or Ir complexes in the copolymerization can help enhance the molecular crystallinity and reduce aggregation, respectively, thereby optimizing the polymer properties. Synthesizing luminescent metal complexes can improve the light‐emitting efficiency of the active layer, thus reducing non‐radiative recombination losses. For perovskite photovoltaics, molecular modifications of CuPc‐based transport layers can still be made to increase the mobility and fine‐tune the energy levels for better alignment. Furthermore, the functional groups on the ferrocene derivatives can passivate the perovskite, and thus these materials can also be used as effective interface layers. For dye‐sensitized solar cells, metal‐containing dye molecules are highly absorbing and stable, making them the preferred choice for dye sensitizers. Future work can involve selecting other metal species to form complexes, but attention must be paid to the complexity of molecular design. All in all, metal complexes have enormous potential in photovoltaic energy conversion. Deeply exploring the relationship between the structure and properties of these materials and continuously optimizing molecular design requires the joint efforts of researchers from various fields.

### 5.2 Functional Metal Complexes for Photocatalysis

The metallated PS is responsible for initiating photo‐induced charge separation within the photocatalytic system and is crucial to the photocatalytic efficiency. While numerous noble‐metal and noble‐metal‐free metal‐complex‐based PSs have been extensively developed, key challenges persist. First, strong and broad visible‐light absorption (achievable by integrating strong light‐absorbing units) is critical to enhancing photon‐to‐electron conversion, which dictates the upper limit of photocatalytic activity. Second, long charge carrier lifetimes, optimized diffusion (short distance, low resistance), and efficient carrier transport between PSs and catalysts are crucial for improving photocatalytic performance. Third, reducing cost and improving stability, excited state lifetime, and catalytic activity of earth‐abundant metal complexes‐based PSs are necessary for practical applications. Additionally, overall solar water‐splitting systems based on metal complexes are still less explored, since studying photocatalytic H_2_ production using only the water reduction half‐reaction is meaningless for practical applications. Therefore, designing catalytic systems that integrate the thermodynamics and kinetics of both half‐reactions to maximize photogenerated carrier surface utilization is highly desirable.

### 5.3 Functional Metal Complexes for Photothermal Conversion

Extending the absorption window into the infrared region of organic/inorganic materials has been a crucial challenge in solar‐energy‐related photothermal applications. Metal complexes offer an effective approach to achieving full‐solar‐spectrum absorption through their unique charge transfer mechanisms, making them promising candidates for photothermal materials. Here, we propose some suggestions for the future development of photothermal metal complexes: (1) To reduce fabrication cost for future industrial production, metal complexes should be designed with cheaper metal centers, such as iron, cobalt, zinc, and nickel. The synthetic methods should be explored and simplified as much as possible to facilitate better large‐scale accumulations via matching suitable ligand structures. (2) Incorporating metal complexes as monomers to construct polymer materials could be a more effective strategy for achieving robust absorption capabilities. Organic functional groups with strong electron‐donating or electron‐withdrawing properties could be further introduced into these polymer backbones to form ICT mechanisms at the same time to regulate the degree of conjugation. (3) The relationships among ligand structure, metal center, and physical/chemical property should be comprehensively investigated through experimental measurements and theoretical calculations. The photo‐to‐thermal conversion mechanisms of metal complexes require an in‐depth study. (4) Device fabrication and optimization based on metal complexes are crucial for their multifunctional photothermal applications. Material treatments, substrate coupling, operating conditions, and stability significantly impact the device performance. Metal complexes represent a significant advancement in the field of photothermal materials. Their unique properties and the ability to tailor their absorption characteristics through molecular design make them highly promising for solar energy conversion and utilization. As research continues to progress, we can expect to see even more innovative applications of these materials in the future.

## Funding

Shenzhen Science and Technology Program (JCYJ20241202130532041), National Natural Science Foundation of China (62205276, 22509176), Hong Kong Research Grants Council (PolyU 15308324), PolyU Research Center for Organic Electronics (1‐CE32), Guangdong Basic and Applied Basic Research Foundation (2023A1515110160), Hong Kong Research Grants Council (PolyU 15301922), RGC Senior Research Fellowship Scheme (SRFS2021‐5S01), Research Institute for Smart Energy (CDAQ), Research Centre for Carbon‐Strategic Catalysis (CE41) and Ms. Clarea Au for the Endowed Professorship in Energy (847S).

## Conflicts of Interest

The authors declare no conflicts of interest.

## Data Availability

The authors have nothing to report.
